# Chromosome‐level genome assembly of the black widow spider *Latrodectus elegans* illuminates composition and evolution of venom and silk proteins

**DOI:** 10.1093/gigascience/giac049

**Published:** 2022-05-25

**Authors:** Zhongkai Wang, Kesen Zhu, Haorong Li, Lei Gao, Huanying Huang, Yandong Ren, Hui Xiang

**Affiliations:** Guangdong Provincial Key Laboratory of Insect Developmental Biology and Applied Technology, Institute of Insect Science and Technology, School of Life Sciences, South China Normal University, Guangzhou, 510631, PR China; School of Ecology and Environment, Northwestern Polytechnical University, Xian, 710072, PR China; Guangdong Provincial Key Laboratory of Insect Developmental Biology and Applied Technology, Institute of Insect Science and Technology, School of Life Sciences, South China Normal University, Guangzhou, 510631, PR China; School of Ecology and Environment, Northwestern Polytechnical University, Xian, 710072, PR China; Guangdong Provincial Key Laboratory of Insect Developmental Biology and Applied Technology, Institute of Insect Science and Technology, School of Life Sciences, South China Normal University, Guangzhou, 510631, PR China; Guangdong Provincial Key Laboratory of Insect Developmental Biology and Applied Technology, Institute of Insect Science and Technology, School of Life Sciences, South China Normal University, Guangzhou, 510631, PR China; School of Ecology and Environment, Northwestern Polytechnical University, Xian, 710072, PR China; Guangdong Provincial Key Laboratory of Insect Developmental Biology and Applied Technology, Institute of Insect Science and Technology, School of Life Sciences, South China Normal University, Guangzhou, 510631, PR China

**Keywords:** chromosome-level genome, Latrodectus elegans, evolutionary rate, venom, spidroin

## Abstract

**Background:**

The black widow spider has both extraordinarily neurotoxic venom and three-dimensional cobwebs composed of diverse types of silk. However, a high-quality reference genome for the black widow spider was still unavailable, which hindered deep understanding and application of the valuable biomass.

**Findings:**

We assembled the *Latrodectus elegans* genome, including a genome size of 1.57 Gb with contig N50 of 4.34 Mb and scaffold N50 of 114.31 Mb. Hi-C scaffolding assigned 98.08% of the genome to 14 pseudo-chromosomes, and with BUSCO, completeness analysis revealed that 98.4% of the core eukaryotic genes were completely present in this genome. Annotation of this genome identified that repetitive sequences account for 506.09 Mb (32.30%) and 20,167 protein-coding genes, and specifically, we identified 55 toxin genes and 26 spidroins and provide preliminary analysis of their composition and evolution.

**Conclusions:**

We present the first chromosome-level genome assembly of a black widow spider and provide substantial toxin and spidroin gene resources. These high-qualified genomic data add valuable resources from a representative spider group and contribute to deep exploration of spider genome evolution, especially in terms of the important issues on the diversification of venom and web-weaving pattern. The sequence data are also firsthand templates for further application of the spider biomass.

## Data Description

### Background

Spiders are a highly diverse and abundant group of predatory arthropods, and more than 49,000 spider species have been described to date [1, 2]. They are found in a wide range of habitats such as underground caves, tropical rainforests, deserts, and glaciers [3–5]. Spiders are of special interest due to their distinctive characteristics, such as spider silk and venom. Spider silk has unique mechanical properties and can potentially be used by the military industry, in medicine, and in other fields [6–8]. Spider venom has a complex composition and is rich in many biologically active substances. This makes it valuable for possible applications in pharmacologic tools, reagents, drug precursors, biological pesticides, and other biologically active substances [9, 10]. In-depth studies of the biochemical and physical properties of spider silk and venom may require the identification of the complete sequence of spider genes, but the lack of high-quality genomic data hinders these studies.

*Latrodectus* spp. are known as black widow spiders. They are featured and of great interest for their extraordinarily neurotoxic venom [11, 12]. Spider venom is a complex mixture of toxins with different biological activities, from small molecular weight compounds to protein and peptide substances. More than 100 different chemical components have been identified in spider venom [13, 14]. Compared with most other venomous animals, black widow spiders contain toxins not only in the venom glands but also in their entire body, including their legs and abdomen. Toxins are also found in spider eggs and newborn offspring. This unique feature makes black widow spider venom components more diverse [15–17]. However, studies on black widow toxins are relatively fragmented, and information on the components of black widow venom still remains limited. Systematic identification and analysis of all the spider toxin genes with full-length sequences is now a top priority [18]. In addition, the black widow spider is distinctive from those spiders that construct classic two-dimensional aerial capture webs. The spider webs of black widow spiders are three-dimensional and are called cobwebs [19–21]. Therefore, genetic deciphering of black widow spider silk provides data and clues for the diversification of spiders. The full lengths of some major silk proteins (spidroins) have been identified, but the systematic analysis on spidrions of cob-weaving spider is still lacking [22–25].

High-quality chromosome-level genomes of *Latrodectus* spp. will provide important resources for deciphering the spider toxin and spidroin genes. Only two spider species (*Trichonephila antipodiana* and *Argiope bruennichi*) have previously been sequenced based on long sequencing reads and assembled to the chromosome level [2, 25]. High-quality chromosome-level spider genome resources remain scarce. In this study, we combined Oxford Nanopore technologies and high-throughput chromosome conformation capture sequencing [26, 27] to generate a high-quality chromosome-level reference genome for *Latrodectus elegans* (NCBI: txid2857379) and systematically analyzed venom proteins and spidroins. These data are a reference for future studies on the range of spider gene diversity.

## Methods

### Biological material, genome DNA extraction, and sequencing

Two female adult *L. elegans* spiders were obtained from Binzhou county, Dali City, Yunnan province, China in 2021. The live samples were sent to Beijing Biomarker Technologies for sample handling, DNA and RNA extraction, and sequencing. Briefly, the spiders were cleansed and grinded in liquid nitrogen, respectively. One spider was used for Hi-C sequencing and RNA sequencing (RNA-seq). The other was for genome sequencing, including next-generation and Nanopore sequencing. Genomic DNA was extracted using a Blood and Cell Culture DNA Mini Kit (Qiagen, Germany) according to the protocol. The short paired‐end insert libraries, including Hi-C and genome sequencing, were constructed using the Illumina platform protocol, and 150-bp paired-end reads were generated using the Illumina NovaSeq platform (Illumina NovaSeq 6000 Sequencing System, U.S America RRID:SCR_020150). A genome long read library was constructed and sequenced on the Nanopore Oxford platform (Oxford Nanopore Technologies, England, RRID: SCR_003756). Total RNAs of the whole body were extracted using TRIzol (Invitrogen, U.S America) according to manufacturer instructions and RNA-seq was generated on the Illumina NovaSeq platform.

### Quality control and genome characteristics evaluation

For the Nanopore long reads, reads with mean quality >7 were retained using an in-house Perl script for further assembly. For the Illumina short reads, the duplicated reads and the adapters were removed. Reads with more than 10% unknown bases or read pairs with more than 30% low-quality bases were also excluded. Each read was removed 5 bp at both head and tail.

To investigate the genome characteristics of *L. elegans*, all the filtered short-insert reads were used for k-mer analysis. The genome size was estimated by using the following formula: G = K_number_/K_depth_, where K_number_ and K_depth_ represent the total number and peak depth of 21-mer, respectively. The genome size was calculated by GenomeScope (GenomeScope, RRID:SCR_017014) v2.0 [28], with the k value set as 21 and other parameters set as default.

### Genome assembly and evaluation

To obtain a high-quality genome, all of the filtered Nanopore long reads were assembled into contigs using Nextdenovo software (v2.4) [29] with core parameters: -d 40 -g 1.74 g. The single-base errors in the genome assembly were corrected using all the filtered Illumina short reads by NextPolish (v1.3.1) with the following parameters: rerun = 3, -max_depth = 100. The Hi-C sequencing reads were mapped to the polished contig assembly to anchor the contigs into chromosomes using the three-dimensional *de novo* assembly software (v170123) [30].

To evaluate the quality and accuracy of the assembled genome, the following three strategies were used. First, the quality of the assembled genome and gene completeness were assessed using BUSCO software (RRID:SCR_015008) v5.2.2 [31] with the core gene set of the eukaryote and metazoan databases, respectively. Second, all the filtered short reads sequenced using the Illumina platform were mapped to the assembled genome by BWA software (RRID:SCR_010910) v0.7.12-r1039 [32] to evaluate the genome integrity. Third, the transcripts of *L. elegans* were assembled using Bridger (RRID: SCR_017039) (version: r2014-12-01) [33] and then mapped to the assembled genome using BLAT software. To perform the synteny analysis, we implemented the Last software (RRID:SCR_006119) (v1066) [34] to achieve the whole-genome alignment using *L. elegans* assembly as the database, in which the “lastal” command was first used to obtain MAF format alignment files and the “maf-swap” command was then used to sort the alignment and select the best one-to-one blocks. After that, Circos (RRID:SCR_011798) v0.69-6 [35] was used to plot the syntenic relationship graph.

### Repetitive sequence annotation

Tandem repeats and transposable elements (TEs) in the assembled genome were both annotated. Tandem Repeat Finder software (v4.09) [36] was used for tandem repeats prediction. The TEs were identified on both protein and DNA levels. On the protein level, the RepeatProteinMask (RM-BLASTX) [37] was used to search TEs using the known protein database. On the DNA level, both *de novo* libraries and Repbase libraries in RepeatMasker (RRID: SCR_012954) (open-4.0.7) were used. *De novo* libraries were built by RepeatModeler (RRID:SCR_015027) [38], and the consensus sequences were used as RepeatMasker input files. The insertion time of each TE sequence was estimated using a Kimura distance-based analysis [39] using parseRM [40].

### Protein-coding gene annotation

To obtain proper gene annotation results, all the TEs were masked before gene annotation. *De novo* annotation, homology-based annotation, and RNA-seq–based annotation were used in this study. First, Augustus software (RRID:SCR_008417) v2.5.5 [41] was used for *de novo* annotation using default parameters. Second, the protein sequences of the gene sets of *Acanthoscurria geniculata* (GCA_000661875.1) [42], *Araneus ventricosus* (GCA_013235015.1) [43], *A. bruennichi* (GCA_015342795.1) [25], *Parasteatoda tepidariorum* (GCF_000365465.2) [44], *Stegodyphus dumicola* (GCF_010614865.1) [45], *Stegodyphus mimosarum* (GCA_000611955.2) [42], *T. antipodiana* (GigaDB) [2], and *Trichonephila clavipes* (GCA_002102615.1) [46] were downloaded from NCBI or GigaDB and used for homology-based predictions, one species at a time. We chose the longest isoform of each gene to obtain the nonredundant protein sequences of each gene set. The protein sequences were used as query to search for orthologous regions in the *L. elegans* genome by tblastn (RRID: SCR_011822) with an e-value of 1e-5. The results were subjected to GeneWise software (RRID:SCR_015054) v2.4.1 [47] to predict gene structure. Third, all the filtered RNA-seq reads were assembled into transcripts using Bridger (version: r2014-12-01) [33] and then aligned to the assembled genome using BLAT (RRID:SCR_011919) (v34, identity >90%, coverage >90%) [48], and PASA (RRID:SCR_014656) [49] was then used to link the spliced alignment. For the results generated from the three methods, EVidenceModeler (RRID: SCR_014659) version 1.1.1 [50] was used to integrate them into the final protein-coding gene set.

These genes were functionally annotated by homology-based searches against InterProScan/GO, KEGG, Swissprot, TrEMBL, and Cog. InterProScan (InterProScan, RRID:SCR_005829) v4.8 [51] was used to screen proteins against five databases (Pfam, release 27.0; prints, release 42.0; prosite, release 20.97; ProDom, 2006.1; and smart, release 6.2). In addition, KEGG [52], SwissProt, TrEMBL, and Cog were used for annotation by BLAST software (NCBI BLAST, RRID:SCR_004870) v2.3.0 [53].

### Orthologous gene identification

The annotated gene sequences of *L. elegans* along with other nine different species, including *A. geniculata* (GCA_000661875.1), *A. bruennichi* (GCA_015342795.1), *P. tepidariorum* (GCF_000365465.2), *T. antipodiana* (GigaDB), *T. clavipes* (GCA_002102615.1), *Centruroides sculpturatus* (GCF_000671375.1), *S. dumicola* (GCF_010614865.1), *S. mimosarum* (GCA_000611955.2), and *Ixodes scapularis* (GCF_016920785.1), were used to identify the orthologous genes using OrthoMCL software (RRID:SCR_007839) v2.0.9 [54] and default parameters.

### Phylogenetic analysis and divergence time estimation

The phylogenetic relationships and divergence time between the 10 test species (*L. elegans, A. geniculata, A. bruennichi, P. tepidariorum, T. antipodiana, T. clavipes, C. sculpturatus, S. dumicola, S. mimosarum*, and *I. scapularis*) were analyzed using the previously identified single-copy genes. All the well-aligned single-copy orthologous genes in each species were concatenated to one super gene for each species. Maximum likelihood–based phylogenetic analysis was conducted using RAxML (RRID:SCR_006086) v8.2.10 [55], and the parameters were set as follows: raxmlHPC-PTHREADS -m PROTGAMMAAUTO -N 100 -p 12 345 -o *I. scapularis* -# 100. Then, the MCMCtree program in the PAML package (RRID:SCR_014932) v4.8 [56] was used for divergence time calculation. All of the fossil records were downloaded from the TimeTree database (RRID: SCR_021162) [57] for result calibration.

### Analysis on relative evolution rate

The well-aligned concatenated protein sequences of single-copy orthologs of the 10 species were used to generate relative evolution rate. Two methods were used: Tajima's relative rate test and two cluster analyses. Tajima's relative rate test was generated by MEGA Software (RRID:SCR_000667) v10 [58]. We specified *I. scapularis* as the outgroup species and tested the relative evolution rate between *L. elegans* and other species. The chi-square test was used to test which species has a faster evolution rate compared to the other one. Two-cluster analysis was generated by LINTRE software via the tpcv model [59]. We also specified *I. scapularis* as the outgroup species and tested the relative evolution rate between *L. elegans* and other species.

### Positive selection analysis

Single-copy orthologs from the five relatively closely related species of orb-web weaving spiders (i.e., *L. elegans, P. tepidariorum, A. bruennichi, T. clavipes*, and *T. antipodiana*) as well as their species tree were used to identify the potential positively selected genes and the genes with positively selected sites in *L. elegans*, using the branch model and branch site model in the Codeml tool of the PAML package (v4.8) [56], respectively. Briefly, the rate ratio (ω) of nonsynonymous to synonymous nucleotide substitutions was estimated. One ratio model was used to detect average ω across the species tree (ω0). For each gene, a two ratio branch model was used to detect the ω of the appointed branch (*L. elegans*) to test the (ω1) and ω of all other branches (ω_background). A likelihood ratio test was performed to compare the fit of the two ratio models with the one ratio model to determine whether the gene was positively selected in the appointed branch (ω1 > ω0; ω1 > ω_background; ω1 > 1; *P* < 0.05). If the gene was not positively selected, then the branch site model was used to detect amino acid sites likely to be positively selected in the appointed branch using Bayes empirical Bayes analysis [56].

### Toxin gene analysis

To locate the toxin genes in the genome of both *L. elegans* and all other related species, we investigated and downloaded toxin sequences from the ArachnoServer database (v3.0) [60], including four families, namely, the CRISP family, the ICK family, the latrodectin family, and the latrotoxin family. For the CRISP family and latrodectin family, blastp (BLASTP, RRID: SCR_001010) was applied to search the candidate toxin genes against protein sequences of all associated species using “-outfmt 7 -evalue 1e-5” as the key parameters. Clustalw2 was used to perform the multiple sequence alignment to align all the latrotoxin gene sets. Because the ICK family is a group of short peptides with the ICK motif, we identified the ICK toxins referring to Haney et al.’s approach [61]. First, we retrieved the peptides less than 200 amino acids and with at least six cysteines constituting the NCNCNCCNCNC (N refers to any other amino acid) motif. Second, we used the online tool ClanTox [62] to evaluate the toxin potential of the above candidate ICK toxins. Third, we used SignalP (RRID: SCR_015644) v5.0 to predict the signal peptide of them, and those without the signal peptide were removed. After further manual check, we finally identified the ICK toxins. As for latrodectin family genes, since there are rather few genes in the ArachnoServer v3.0 database, we then retrieved the known sequences of latrodectin from NCBI. We applied the Blastp with an e-value cutoff of 10-5 to search the candidate homolog proteins. Then the homolog was further verified by HMMER (RRID:SCR_005305) v3.3 [63]. In brief, all above-found sequences were merged together, with Clustalw2 applied to perform the multiple-sequence alignment, and were then transformed into the stockholm format using an online toolset [64]. The aligned sequences were used to construct the HMM profile using hmmbuild, a binary software of HMMER v3.3. Finally, hmmsearch was applied for the peptide of each species to find the potential domains of all species by using key parameters of “–domtblout” and an e-value of 10-5.

The toxin gene distribution of *L. elegans* was plotted by MG2C software [65]. We then constructed the phylogeny of the latrotoxin gene set using maximum likelihood methods in RAxML software (v8.2.10) [55], in which raxmlHPC-PTHREADS-AVX was used as the main model with 100 bootstrap replicates, the latrotoxin gene of *I. scapularis* was specified as the outgroup, and the key parameters were set as “-n orthology -m PROTGAMMAAUTO -f a -x 12 345 -N 100 -p 12 345 -o *I. scapularis*_Latrotoxins_XP_040064028.1.” Finally, the phylogenetic tree of the latrotoxin genes was visualized in iTOL software (RRID: SCR_018174) [66, 67]. ParaAT [68] was applied to achieve multiple protein-coding DNA alignments using “-m muscle -p proc -f axt” as key parameters. Then we calculated the ratio of nonsynonymous substitutions over synonymous substitutions (Ka/Ks) for each pair of genes separately using KaKs Calculator software (v2.0) [69]. All Ka/Ks values were plotted and grouped by species.

### Analysis of spidroins

To annotate the various types of spidroins (MaSp, MiSp, TuSp, AcSp, Flag, AgSp, and PySp) of *L. elegans*, we initially downloaded the amino acid sequences of spidroins of *Latrodectus hesperus, T. clavipes, A. bruennichi*, and *A. ventricosus* from the NCBI database (ABR68855.1, ABR68856.1, ARA91152.1, ARA91182.1, AWK58725.1, AFP57565.1, AFX83557.1, AAY28931.1, ACV41934.1, PRD24320.1, PRD20448.1, PRD23654.1, PRD24510.1, PRD30268.1, PRD24772.1, GFY36469.1, PRD26655.1, PRD23989.1, GFY34959.1, PRD26201.1, PRD35275.1, GFY35027.1, ADK92884.1, AFN54363.1, AGB35874.1, GBM54680.1, GBN00528.1, GBN00527.1, GBN25680.1, AFV31615.1, GBM96188.1, GBN20389.1, GBN20387.1, GBL96802.1, GBL96803.1, GBN70256.1, AUH99620.1, QKE59598.1, QKE59599.1, QBA85221.1, GBN88500.1). Using the seven different types of spider silk genes collected above as queries, we searched the annotated proteins of *L. elegans* by blastp with the cutoff e-value of less than 10^−5^. The retrieved sequences of *L. elegans* were then manually filtered to remove missense mutation sequences according to the conserved domains of the spidroins. These spidroin sequences of *L. elegans* were further verified by blastp against the NCBI nonredundant protein database [70]. The chromosome distribution of the spidroin genes was then plotted by MG2C software (v2.1). Sequences were aligned using MEGA software (v10) and further reorganized as categories for further research: complete, internal gap, N-terminal, C-terminal, and repetitive sequence. To identify the spidroins in the other two spider species (i.e., *P. tepidariorum* and *T. antipodiana*), we searched the annotated nonredundant protein sequences of the related species by blastp with a cutoff e-value of 1e-5, an identity of 0.3, and an alignment ratio of 0.5.

## Results and Discussion

### Genome assembly

**Figure 1:**
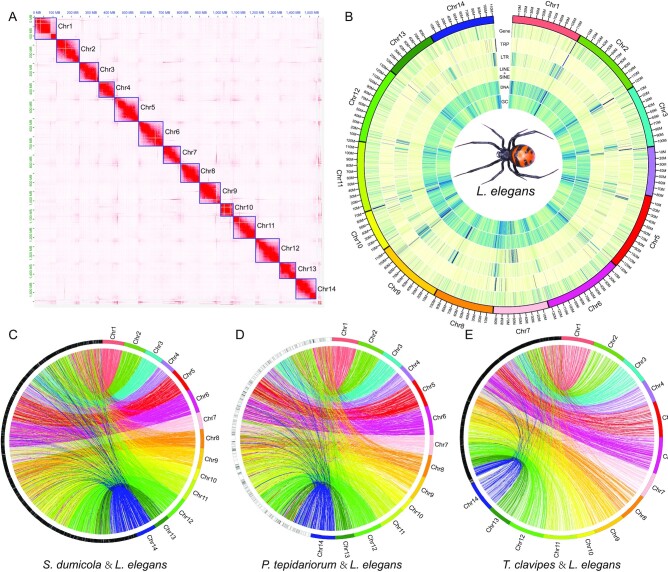
Genome assembly and comparative analysis of *L. elegans*. **A**. Heatmap of chromosome interactions in *L. elegans*. **B**. Circos plot of distribution of the genomic elements in *L. elegans*. From the outer ring to the inner ring are the distributions of protein-coding genes, tandem repeats (TRs), long tandem repeats (LTRs), short/long interspersed nuclear elements (SINEs/LINEs), DNA elements, and GC content, respectively. **C**. Genomic synteny between *S. dumicola* and *L. elegans*. **D**. Genomic synteny between *P. tepidariorum* and *L. elegans*. **E**. Genomic synteny between *T. clavipes* and *L. elegans*.

First, we generated 66.49 Gb Illumina short-insert-size reads with almost 38.20× depth (Supplementary Table 1). The 21-mer analysis showed that the genome size of *L. elegans* is ∼1.74 Gb (Supplementary Fig. 1). Then, 106.80 G filtered Nanopore reads (N50 is 24.01 Kb, ∼61.37-fold of the genome) were obtained (Supplementary Table 2). The Nanopore filtered reads were assembled into contigs and further assembled into chromosomes using Hi-C reads (77.58 Gb, ∼44.57-fold of the genome) (Supplementary Table 3). We obtained a 1.57-Gb genome assembly with contig N50 of 4.34 Mb and scaffold N50 of 114.31 Mb (Supplementary Table 4). A total of 14 chromosomes were assembled with lengths ranging from 70.40 to 133.92 Mb (Fig. 1A, Supplementary Table 5). The number of chromosomes is consistent with a previous report on female window spiders [71]. To validate the completeness and accuracy of the genome of *L. elegans*, assembled transcript mapping ratio, short read mapping ratio, and BUSCO (v5.2.2) were used in the analysis. All the assembled transcripts were aligned to the genome, and 77,191 of 85,772 (90.00%) transcripts can be found in the assembled genome (Supplementary Tables 6–8). We aligned all the filtered short reads to the assembled genome, and more than 357.88 million reads (99.28%) could be mapped to the genome (Supplementary Table 9). We also found that 251 of 255 (98.4%) and 930 of 954 (97.5%) core eukaryote and metazoan genes were successfully identified in the genome, respectively (Supplementary Table 10), and this assembly quality was comparable with that of the close-related species (Supplementary Table 11). We also identified the Hox genes in all 10 species (*A. geniculata, S. mimosarum, T. clavipes, A. bruennichi, P. tepidariorum, C. sculpturatus, S. dumicola, I. scapularus, T. antipodiana*, and *L. elegans*). These results showed that *L. elegans* has two Hox gene clusters. Both of these two clusters are complete and continuous, which is comparable to other related species (Supplementary Fig. 2) [2, 25, 44]. These results indicate that the integrity and accuracy of the assembled genome are good.

### Genome annotation

Both tandem repeats and TEs were annotated in the assembled genome, and a total of ∼506.09 Mb repeat sequences were identified that accounted for 32.30% of the assembled genome (Supplementary Table 12). For TEs, there were 9.69% of DNA (151.78 Mb), 4.50% of long interspersed nuclear elements (70.53 Mb), 2.48% of long tandem repeats (38.89 Mb), and 1.09% of short interspersed nuclear elements (17.15 Mb) in this genome (Supplementary Table 13). For protein-coding genes, 20,167 genes were annotated, with 81.03% of the genes having homologous hits in the public databases (Supplementary Table 14). These genes showed high similarity to related species in gene length distribution, CDS (coding sequence) length distribution, exon length distribution, and exon number distribution (Supplementary Fig. 3). The basic genome statistics of this genome, including gene density, tandem repeats, long tandem repeats, long interspersed nuclear elements, short interspersed nuclear elements, DNA TEs, and GC content, are shown in Fig. 1B. We checked the synteny block between *L. elegans* and other species of Arachnida (*S. dumicola,P. tepidariorum*, and *T. clavipes*, Fig. 1C–E). The results showed that the assembled genome has a good genome synteny relationship with these species.

### Phylogenetic relationship of *L. elegans* and other related species

**Figure 2:**
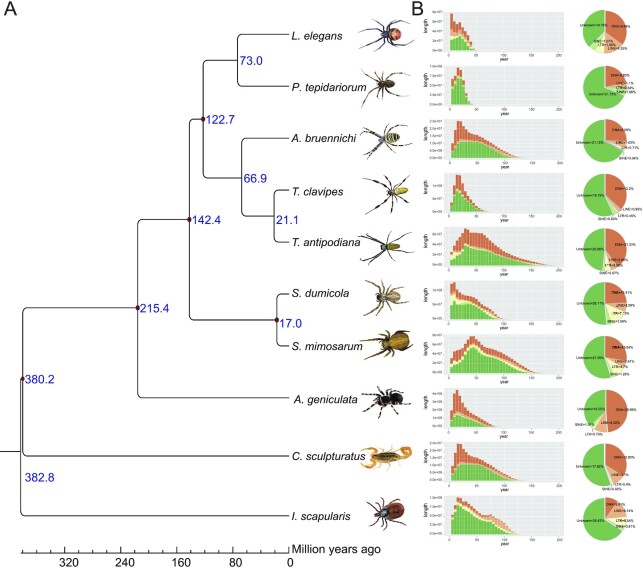
Comparative genomics of *L. elegans* and related species. **A**. Phylogenetic relationships among these species. The red dot at the node represents a fossil record that was used for the calibration of the divergence time. The blue number in each node represents its divergence time for species. The red and green numbers in each node/species represent the expanded/contracted gene families, respectively. **B**. Comparison of the insertion history of transposable elements among species. The x-axis represents the inferred insertion time (unit: million years ago) of transposable elements in the genome. The y-axis represents the total/each length of the transposable element in each species.

To compare the genomics of *L. elegans* with other spider species, we identified the orthologous/paralogous genes among these species. A total of 28,587 gene families were clustered in these eight spiders and two outgroup species, and 156 single-copy genes were identified. The phylogenetic relationship of these 10 species was determined using the amino acid and nucleotide acid sequences of CDS, and the fourfold degenerate synonymous site (4dTV) [72] of the single-copy genes was concatenated into a super-gene in each species. Each method showed the same phylogenetic relationship with high bootstrap values (Supplementary Figs. 4–6). This relationship is consistent with the well-documented spider tree of life [73]. *L. elegans* is closely related to *P. tepidariorum*, both of which are of Theridiids. A calculation of the estimated divergence time suggested that the two species diverged ∼73.0 million years ago (Fig. 2A).

### TE insertion history of Arachnida

We checked the types of TEs and the TE insertion time of all 10 species and found that the TE contents are significantly different. In the Theridiidae, including *L. elegans* and *P. tepidariorum*, the TE insertion times are concentrated at 20 to 35 million years ago. However, the TE insertion times of other species are much older than that of the Theridiidae. Besides, the insertion times of *L. elegans* (∼35 million years) and *P. tepidariorum* (∼20 million years) are much more recent than the divergence between two species, which suggests that the TE insertion event may have happened after their divergence (Fig. 2B).

### Relative evolution rate of species

Species in different environments may face different selection pressures, and the relative rate of evolution can reflect this. The relative evolution rate results showed that *L. elegans* had the fastest evolution rate among these species and suggested that it has experienced strong selection pressure. *A. geniculata* had the slowest evolution rate (Supplementary Fig. 7; Supplementary Tables 15–16). It is the only species from the relatively ancient Mygalomorphae. The result suggests that a relatively ancient spider group may have relatively less selection pressure in their habitats. However, due to its low genome quality, this result is pending further verification.

### Positively selected genes

Using five relatively closely related species of orb-web weaving spiders belonging to the Araneoidea (i.e., *L. elegans, P. tepidariorum, A. bruennichi, T. clavipes*, and *T. antipodiana*), we identified 8 positively selected genes and 348 genes with positively selected sites in *L. elegans* (Supplementary Table 17). In these genes, *lhx9* was the only gene related to gonadal development. The structures of this gene in these species were constructed, and *L. elegans* was the most unique of all species (Supplementary Fig. 8). These results indicated that the gonadal development of *L. elegans* may differ from that of the other species. The *CaMKI* gene belongs to the calcium/calmodulin-dependent protein kinase family, and the other gene, *CaMKII*, is associated with OA (osteoarthritis) signaling, which may affect Ca^2+^ signaling or adjust intracellular cAMP levels in vivo. In the spider *Cupiennius salei,CaMKII* may also be a downstream modulator of OA signaling in spider VS-3 neurons [74], which is related to cell excitability.

### Venom gene analysis

**Figure 3:**
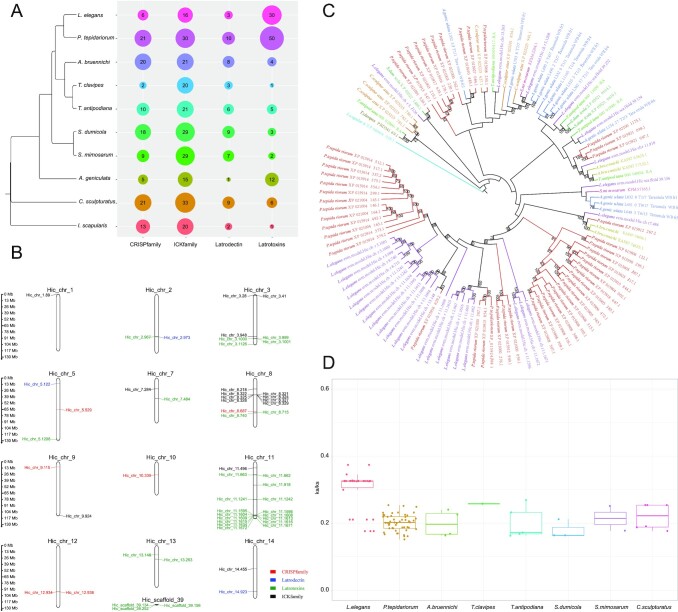
Toxin genes in the Arachnida species. **A**. Different toxin gene numbers in these species. **B**. The distribution of toxin genes in *L. elegans*. **C**. The phylogenetic relationship of latrotoxin genes in these species. **D**. Latrotoxin gene Ka/Ks value of these species. The latrotoxin gene in *I. scapularis* was used as the reference.

The venoms of *Latrodectus* spp. are famous for their potency and ability to cause extreme and long-lasting pain. Based on genome-wide comparative analysis, we identified members of the four major venom gene families: CRISP family, ICK family, latrodectin family, and latrotoxin family. The severe symptoms of *Latrodectus* envenomation are largely attributed to latrotoxins [75]. Consistently, we found that, compared to other spiders, the number of genes in the latrotoxin family of *L. elegans* and the house spider *P. tepidariorum* has undergone lineage-specific expansion. The number of latrotoxin genes found in *P. tepidariorum* and *L. elegans* was 30 and 50, respectively, which is more than that found in other spiders (1–12) (Fig. 3A). We also identified six toxins from the CRISP family and two from the ICK family in *L. elegans*, respectively. The latrotoxin and CRISP families are both ancient and relatively conserved toxins that exist in all the test species of Arachnida. Toxins from the ICK family seem unique to the Araneoidea (Fig. 3A). Genic loci of all these venom toxins were mapped on 11 chromosome scaffolds (chromosomes 2, 3, 5, 7, 8, 9, 10, 11, 12, 13 and scaffold 39). The majority of latrotoxin genes were located on chromosome 11, and there was a remarkable tandem duplication of latrotoxin genes on chromosome 11 (Fig. 3B). Phylogenetic analysis showed that the latrotoxins experienced substantial gene duplication and diversification in the two Theridiidae spiders, including *L. elegans* and *P. tepidariorum*, and that latrotoxins of *L. elegans* in the clade of latest expansion were mostly located on chromosome 11 (Fig. 3C). We analyzed the ratio of nonsynonymous to synonymous nucleotide substitution rate (Ka/Ks ratio) of each latrotoxin gene pair and found that *L. elegans* latrotoxin genes had generally higher Ka/Ks ratios compared to those in other species, suggesting their rapid evolution (Fig. 3D).

### Spidroin gene analysis

**Figure 4:**
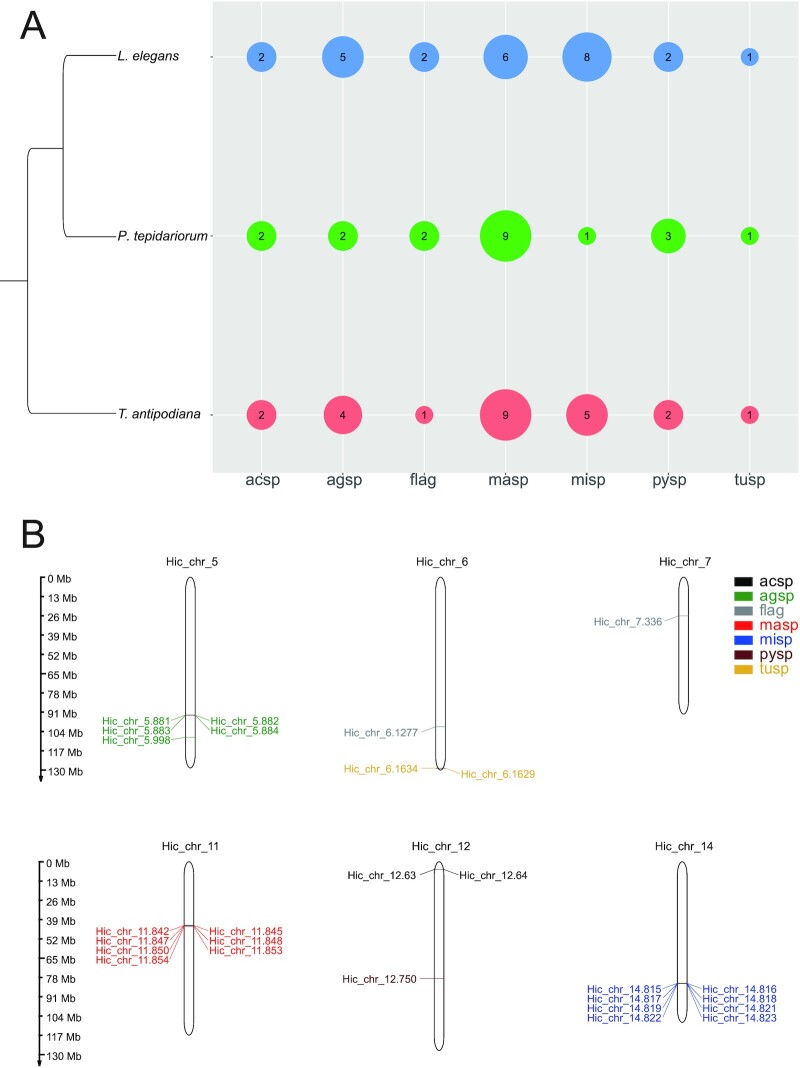
Spidroin genes in the Arachnida species. **A**. Different spidroin gene numbers in three spider species. **B**. The chromosome distribution of spidroin genes in *L. elegans*.

A female cob-weaving spider can have up to seven morphologically differentiated types of silk glands, each of which can produce a silk protein, namely, spidroin. The classes of spidroins include major ampullate spidroin (*MaSp*), minor ampullate spidroin (*MiSp*), flagelliform spidroin (*Flag*), aggregate spidroin (*AgSp*), aciniform spidroin (*AcSp*), tubuliform spidroin (*TuSp*) and pyriform spidroin (*PySp*). We identified six unique annotated genes for *MaSp*, eight for *MiSp*, two for *Flag*, five for *AgSp*, two for *PySp*, two for *AcSp*, and one for *TuSp* in *L. elegans* (Fig. 4A). It is notable that *L. elegans* has relatively more *Misp* genes. MiSp is mainly used for inelastic temporary spirals during web building. However, in cobweb spiders, *Misp* contributes to prey-wrapping in cobweb weavers [76]. The more copies of *Misps* in the*L. elegans* genome might evolve to strength the function of prey-wrapping [77]. All *MiSps* were clustered on chromosome 14, suggesting that they may have diversified via tandem duplication (Fig. 4B). Other multicopy spidroin genes such as *MaSp, AgSp, PySp*, and *AcSp* were also distributed in clusters on chromosomes 11, 5, 6, and 12, respectively, suggesting that tandem duplication is the main type of duplication of spidroins. The two *Flag* genes, however, are located on chromosomes 6 and 7 (Fig. 4B).

## Conclusion

Using the approach combining data of Illumina short reads, Nanopore long reads, and Hi-C reads, we assembled and annotated the first chromosome-level 1.57-Gb large genome of a black widow spider, *L. elegans*. In this study, we confirmed phylogenetic position of this species in the spider tree of life. In addition, by analysis of the Hox gene family, we again verified it as high quality. Specifically, we focused on toxin and spidroin genes, which contribute to the distinctive features of black widow and cobweb-weaving spiders, and provided substantial information in terms of their composition and numbers and preliminarily demonstrate the evolution pattern of one important toxin gene family, latrotoxins. The important venom toxins contribute greatly to black widow spiders' toxicity, and they showed fast evolution. Generally, the genome resource data will help for deep exploration of spider genome evolution, especially on diversification of venom and web-forming. The sequence data are also firsthand templates for further application of the spider biomass.

## Data availability

All raw sequencing data and the genome assembly of *L. elegans* underlying this article are available at the NCBI and can be accessed with Bioproject ID PRJNA745004. All supporting data and materials are available in the *GigaScience* GigaDB database [78].

## Additional files

**Supplementary Table 1**. The statistics of sequencing reads on the Illumina platform.

**Supplementary Table 2**. The statistics of sequencing reads on the Nanopore platform.

**Supplementary Table 3**. The statistics of Hi-C sequencing reads.

**Supplementary Table 4**. The statistics of the polished genome and chromosome-level genome.

**Supplementary Table 5**. Statistics of the assembled chromosome-level genome via 3D *de novo* assembly software.

**Supplementary Table 6**. The statistics of RNA sequencing reads on the Illumina platform.

**Supplementary Table 7**. The statistics of the assembled transcripts by Bridger of five organs/tissues.

**Supplementary Table 8**. The statistics of the transcript mapping ratio on the assembled genome.

**Supplementary Table 9**. The statistics of the short read mapping ratio on the assembled genome.

**Supplementary Table 10**. The quality evaluation of the assembled genome by BUSCO software.

**Supplementary Table 11**. Comparison of the related genomes with our chromosome-level genome.

**Supplementary Table 12**. The statistics of the annotated repeat sequences in our assembled genome.

**Supplementary Table 13**. The statistics of the annotated repeat sequences in our assembled genome by *de novo* prediction.

**Supplementary Table 14**. The functional annotation of the predicted protein-coding genes.

**Supplementary Table 15**. Relative evolution rate among these species by LINTRE software.

**Supplementary Table 16**. Relative evolution rate among these species by MEGA software.

**Supplementary Table 17**. Statistics of positively selected genes of *L. elegans*.

**Supplementary Figure 1**. The 21-mer analysis of the *L. elegans* genome.

**Supplementary Figure 2**. Annotation and comparison of the Hox clusters among these 10 species.

**Supplementary Figure 3**. Distribution of gene parameters in various species.

**Supplementary Figure 4**. Phylogenetic relationship among the 10 species inferred by the amino acid sequences of the single-copy genes.

**Supplementary Figure 5**. Phylogenetic relationship among the 10 species inferred by the nucleotide acid sequences of the single-copy genes.

**Supplementary Figure 6**. Phylogenetic relationship among the 10 species inferred by the *4dTV* data of the single-copy genes.

**Supplementary Figure 7**. Relative evolutionary rate of species.

**Supplementary Figure 8**. Gene structure of *lhx9* in these species.

giac049_GIGA-D-21-00338_Original_Submission

giac049_GIGA-D-21-00338_Revision_1

giac049_Response_to_Reviewer_Comments_Original_Submission

giac049_Reviewer_1_Report_Original_Submission Zewen Liu, Ph.D. -- 11/24/2021 Reviewed

giac049_Reviewer_2_Report_Original_Submission Alejandro SÃ¡nchez-Gracia -- 12/1/2021 Reviewed

giac049_Reviewer_2_Report_Revision_1 Alejandro SÃ¡nchez-Gracia -- 2/28/2022 Reviewed

giac049_Reviewer_3_Report_Original_Submission Jesper Bechsgaard -- 12/7/2021 Reviewed

giac049_Reviewer_3_Report_Revision_1 Jesper Bechsgaard -- 3/14/2022 Reviewed

giac049_Supplemental_Tables_and_Figures

## Abbreviations

BUSCO: Benchmarking Universal Single-Copy Orthologs; RNA-seq: RNA sequencing; TE, transposable element.

## Competing interests

The authors declare that they have no competing interests.

## Authors’ contributions

HX and YR conceived and designed the investigation. KZ, LG, and HH performed field and laboratory work. ZW assembled the genome. HL performed the Hi-C scaffold. YR, KSZ, and LG analyzed the data. HX, KS, and LG contributed materials and reagents. YR and KSZ wrote the paper. HX and YR revised the manuscript. All the authors read and approved the final manuscript.
