# Supplementary material for: Chromosome‐level genome assembly of the black widow spider Latrodectus elegans illuminates composition and evolution of venom and silk proteins
Source: Gigascience. 2022 May 25;11:giac049. doi: 10.1093/gigascience/giac049 (PMC9154082; doi:10.1093/gigascience/giac049)
Supplement: giac049_GIGA-D-21-00338_Original_Submission [file giac049_giga-d-21-00338_original_submission.pdf]

GigaScience

Chromosome-level genome assembly of the black widow spider Latrodectus elegans illuminates composition and evolution of venom and silk proteins

--Manuscript Draft--

Manuscript Number:	GIGA-D-21-00338
Full Title:	Chromosome-level genome assembly of the black widow spider Latrodectus elegans illuminates composition and evolution of venom and silk proteins
Article Type:	Data Note
Funding Information:	National Natural Science Foundation of China (32070411 , 32000383) — Prof. Hui Xiang<br>Natural Science Foundation of Guangdong Province (2019A1515011012) — Prof. Hui Xiang<br>Laboratory of Lingnan Modern Agriculture Project (NZ2021019) — Prof. Hui Xiang
Abstract:	Background<br><br>Black widow spider has both extraordinarily neurotoxic venom and three-dimensional cob-webs composing of diverse types of silk. However, high-quality reference genome for black widow spider was unavailable, which hindered deep understanding and application of the valuable biomass.<br><br>Results<br><br>We assembled the L. elegans genome using Nanopore long-reads. The genome size is 1.57 Gb with contig N50 of 114.31 Mb. Hi-C scaffolding assigned 98.08% of the genome to 14 pseudo-chromosomes, and BUSCO (Benchmarking Universal Single-Copy Orthologs) completeness analysis revealed that 90.00% of the core eukaryotic genes were completely present in the genome assembly. Annotation of the L. elegans genome assembly identified that repetitive sequences account for 506.09 Mb (32.30%) and 20167 protein-coding genes, among which 81.03% have functional annotation terms. Phylogenetic analysis showed that L. elegans is closely related to the house spider Parasteatoda tepidariorum (Theridiidae) and that they diverged from a common ancestor ~73.0 million years ago. The relatively high evolution rate suggests that L. elegans evolved under strong selection pressure. Based on genome-wide comparative analysis, we identified and 39 toxin proteins and 26 spidroin. Among the toxins, latrotoxins experienced substantial gene duplication and diversification in the two Theridiidae spiders. L. elegans latrotoxin genes had higher Ka/Ks ratios compared to those in other species, suggesting rapid evolution of them. We found that L. elegans have remarkably more MiSp and tandem duplication is the main duplication event of the spidroin.<br><br>Conclusions<br><br>The high-quality L. elegans assembled genome illuminates composition and evolution of venom and silk proteins provides a resource for in-depth exploration and application of them.
Corresponding Author:	Hui Xiang, PH.D<br>South China Normal University<br>Guangzhou, CHINA
Corresponding Author Secondary Information:	
Corresponding Author's Institution:	South China Normal University
Corresponding Author's Secondary Institution:	

Powered by Editorial Manager® and ProduXion Manager® from Aries Systems Corporation

**First Author:**	Zhongkai Wang
**First Author Secondary Information:**	
**Order of Authors:**	Zhongkai Wang
	Kesen Zhu
	Haorong Li
	Lei Gao
	Huanying Huang
	Yandong Ren
	Hui Xiang
**Order of Authors Secondary Information:**	
**Additional Information:**	
**Question**	**Response**
Are you submitting this manuscript to a special series or article collection?	No
**Experimental design and statistics**<br><br>Full details of the experimental design and statistical methods used should be given in the Methods section, as detailed in our Minimum Standards Reporting Checklist. Information essential to interpreting the data presented should be made available in the figure legends.<br><br>Have you included all the information requested in your manuscript?	Yes
**Resources**<br><br>A description of all resources used, including antibodies, cell lines, animals and software tools, with enough information to allow them to be uniquely identified, should be included in the Methods section. Authors are strongly encouraged to cite Research Resource Identifiers (RRIDs) for antibodies, model organisms and tools, where possible.<br><br>Have you included the information requested as detailed in our Minimum Standards Reporting Checklist?	Yes

Powered by Editorial Manager® and ProduXion Manager® from Aries Systems Corporation

**Availability of data and materials**<br><br>All datasets and code on which the conclusions of the paper rely must be either included in your submission or deposited in publicly available repositories (where available and ethically appropriate), referencing such data using a unique identifier in the references and in the "Availability of Data and Materials" section of your manuscript.<br><br>Have you have met the above requirement as detailed in our Minimum Standards Reporting Checklist?	Yes

Powered by Editorial Manager® and ProduXion Manager® from Aries Systems Corporation

Manuscript

Click here to access/download;Manuscript;spiderGonomeMS_20201008sub

Chromosome- level genome assembly of the black widow spider *Latrodectus elegans* illuminates composition and evolution of venom and silk proteins

Zhongkai Wang[1, 2, †], Kesen Zhu[1, †], Haorong Li[2, †], Lei Gao[1], Huanying Huang[1], Yandong Ren[2, *], and Hui Xiang[1, *]

1. Guangdong Provincial Key Laboratory of Insect Developmental Biology and Applied Technology, Institute of Insect Science and Technology, School of Life Sciences, South China Normal University, Guangzhou, 510631, PR China.

2. School of Ecology and Environment, Northwestern Polytechnical University, Xian, 710072, PR China.

† These authors contributed equally to this work.

[*] These authors jointly directed this work. Correspondence should be addressed to H. X. (xiang_shine@foxmail.com), Y. R. (renyandong90@126.com).

Abstract

Background: Black widow spider has both extraordinarily neurotoxic venom and three-dimensional cob-webs composing of diverse types of silk. However, high-quality reference genome for black widow spider was unavailable, which hindered deep understanding and application of the valuable biomass. **Results:** We assembled the *L. elegans* genome using Nanopore long-reads. The genome size is 1.57 Gb with contig N50 of 4.34Mb and scaffold N50 of 114.31 Mb. Hi-C scaffolding assigned 98.08% of the genome to 14 pseudo-chromosomes, and BUSCO (Benchmarking Universal Single-Copy Orthologs) completeness analysis revealed that 98.4% of the core eukaryotic genes were completely present in the genome assembly. Annotation of the *L. elegans* genome assembly identified that repetitive sequences account for 506.09 Mb (32.30%) and 20167 protein-coding genes, among which 81.03% have functional annotation terms. Phylogenetic analysis showed that *L. elegans* is closely related to the house spider *Parasteatoda tepidariorum* (Theridiidae) and that they diverged from a common ancestor ∼73.0 million years ago. The relatively high evolution rate suggests that *L. elegans* evolved under strong selection pressure. Based on genome-wide comparative analysis, we identified and 39 toxin proteins and 26 spidroin. Among the toxins, latrotoxins experienced substantial gene duplication and diversification in the two Theridiidae spiders. *L. elegans* latrotoxin genes had higher Ka/Ks ratios compared to those in other species, suggesting rapid evolution of them. We found that *L. elegans* have remarkably more MiSp and tandem duplication is the main duplication event of the spidroin. **Conclusions:** The high-quality *L. elegans* assembled genome illuminates composition and

evolution of venom and silk proteins provides a resource for in-depth exploration and application of them.

Keywords： Chromosome-level genome, *Latrodectus elegans*, evolutionary rate, venom, spidroin

Data Description

Background

Spiders is a highly diverse and abundant group of predatory arthropods and more than 49,000 spider species have been described to date[1, 2]. Due to their adaptability and diverse behaviors, they are found in a wide range of habitats such as underground caves, tropical rainforests, deserts, and on glaciers [3-5]. Spider diversity is associated with their distinctive characteristics, especially spider silk and venom. Spider silk has unique mechanical properties and can potentially be used by the military industry, in medicine and in other fields [6-8]. Spider venom has a complex composition and is rich in many biologically active substances. This makes it valuable for possible applications in pharmacological tools, reagents, drug precursors, biological pesticides and other biologically active substances [9, 10]. In-depth studies of the biochemical and physical properties of spider silk and venom may require the identification of the complete sequence of spider genes, but the lack of high-quality genomic data hinders these studies.

Several *Latrodectus* spp. are known as black widow spiders. They are featured and of great interest for their extraordinarily neurotoxic venom neurotoxic venom [11, 12]. Spider

venom is a complex mixture of toxins with different biological activities, from small molecular weight compounds to protein and peptide substances. More than 100 different chemical components have been identified in spider venom [13, 14]. Compared with most other venomous animals, black widow spiders not only contain toxins in the venom glands but also in their entire body, including their legs and abdomen. Toxins are also found in spider eggs and newborn offspring. This unique feature makes black widow spider venom components more diverse [15-17]. However, studies on black widow toxins are relatively fragmented, and information on the components of black widow venom remains limited. Systematic identification of the complete spider toxin gene sequence is now a top priority [18]. In addition, the black widow spider is distinctive from those spiders that construct classic two-dimensional aerial capture webs. The spider webs of black widow spiders are three-dimensional and are called cob-webs [19-21]. Therefore, genetic deciphering of black widow spider silk provides data and clues for the diversification of spiders. The full lengths of some major silk proteins (spidroins) have been identified, but the systematic analysis on spidrion of cob-weaving spider is still lacking [22, 23].

High-quality chromosome-level genomes of *Latrodectus* spp. will aid the identification and understanding of the complete spider toxin and spider silk genes. Only two spider species (*Trichonephila antipodiana* and *Argiope bruennichi*) have previously been sequenced based on long sequencing reads and assembled to the chromosome level [2, 24]. High-quality chromosome-level spider genome resources remain scarce. In this study, we combined Oxford Nanopore technologies and high-throughput chromosome conformation capture sequencing [25, 26] to generate a high-quality chromosome-level reference genome for

Latrodectus elegans and systematically analyzed venom proteins and spidrons. These data are a reference for future studies on the range of spider gene diversity.

Methods

Genome DNA extraction and library construction

One female *L. elegans* was obtained from Yunnan province, China in 2021. It was cleansed, and genomic DNA was extracted using a Blood and Cell Culture DNA Mini Kit (Qiagen) according to the protocol. The short paired-end inserts libraries, including Hi-C and genome data, were constructed using the Illumina platform protocol, and 150-bp paired-end reads were generated using the Illumina NovaSeq platform. A genome long read library was constructed and sequenced on the Nanopore oxford platform. Total RNAs of the whole body were extracted using TRIzol (Invitrogen) according to manufacturer instructions and sequenced on the Illumina NovaSeq platform.

Quality control and genome characteristics evaluation

For the Nanopore long reads, reads with mean quality > 7 were retained using Perl scripts for further assembly. For the Illumina short reads, the duplicated reads and the adaptors were removed. Reads with more than 10% unknown bases or read pairs with more than 30% low-quality bases were also excluded. Each read was removed 5 bp at both head and tail.

To investigate the genome characteristics of *L. elegans*, all the filtered short-insert reads were used for k-mer analysis. The genome size was estimated by using the formula: $G = K_{number}/K_{depth}$, where K_{number} and K_{depth} represent the total number and peak depth of 21-mer, respectively. The genome size was calculated by GenomeScope (v2.0) [27], with the k value

set as 21 and other parameters set as default.

Genome assembly and evaluation

To obtain a high-quality genome, all of the filtered Nanopore long reads were assembled into contigs using Nextdenovo software (v2.4) [28] with core parameters: -d 40 -g 1.74 g. The single-base errors in the genome assembly were corrected using all the filtered Illumina short reads by NextPolish (v1.3.1) with parameters: rerun = 3, -max_depth = 100. The Hi-C sequencing reads were mapped to the polished contig assembly to anchor the contigs into chromosomes using the 3D *de novo* assembly software (v170123) [29].

To evaluate the quality and accuracy of the assembled genome, the following three strategies were used. First, the quality of the assembled genome and gene completeness were assessed using BUSCO software (v5.2.2) [30] with the core gene set of the eukaryote and metazoan databases, respectively. Second, all the filtered short reads sequenced using the Illumina platform were mapped to the assembled genome by BWA-MEM software (v0.7.12-r1039) [31] to detect the genome integrity. Third, the transcripts of *L. elegans* were assembled using Bridger (version: r2014-12-01) [32] and then mapped to the assembled genome using BLAT software.

Repetitive sequence annotation

Tandem repeats and Transposon elements (TEs) in the *L. elegans* genome were both annotated. Tandem Repeat Finder software (v4.09) [33] was used for tandem repeats prediction. The TEs were identified on both protein and DNA levels. On the protein level, the RepeatProteinMask (RM-BLASTX) [34] was used to search TEs using the known protein database. On the DNA level, both *de novo* libraries and Repbase libraries in RepeatMasker

(open-4.0.7) were used. *De novo* libraries were built by RepeatModeler [35] (http://www.repeatmasker.org/RepeatModeler/), and the consensus sequences were used as RepeatMasker input files. After obtaining the TE sequences, we calculated the insertion time of each TE sequence. The insertion time of each TE sequence was calculated using the following formula: Insertion time = Kimura value/2 × evolution rate. The Kimura value in this formula was extracted from the RepeatMasker results, and the evolution rate of each species was calculated using the r8s model in PAML package [36].

Protein-coding gene annotation

To obtain proper gene annotation results, all the TEs were masked before gene annotation. *De novo* annotation, homology-based annotation, and RNAseq-based annotation were used in this study. First, Augustus software (v2.5.5) [37] was used for *de novo* annotation using default parameters. Second, the protein sequences of *Acanthoscurria geniculata* (GCA_000661875.1), *Araneus ventricosus* (GCA_013235015.1), *Argiope bruennichi* (GCA_015342795.1), *Parasteatoda tepidariorum* (GCF_000365465.2), *Stegodyphus dumicola* (GCF_010614865.1), *Stegodyphus mimosarum* (GCA_000611955.2), *Trichonephila antipodiana* (GigaDB) and *Trichonephila clavipes* (GCA_002102615.1) were downloaded from NCBI or GigaDB, and the longest transcripts were chosen for further analysis. All the remaining genes were aligned using *tblasn* with an e-value of 1e-5. The *blast* result formats were changed and prepared for predicting gene structures using GeneWise (V2.4.1) [38]. Third, all the filtered RNA-seq reads were assembled into transcripts using Bridger (version: r2014-12-01) [32] and then aligned to the assembled genome using BLAT (v34, identity > 90%, coverage > 90%)[39], and the PASA [40] was then used to link the spliced alignment.

For the results generated from the three methods, EvidenceModeler (version 1.1.1) [41] was used to integrate them into the final protein-coding gene set.

These genes were functionally annotated by homologous searching against InterProScan/GO, KEGG, Swissprot, TrEMBL and Cog. InterProScan (v4.8) [42] was used to screen proteins against five databases (Pfam, release 27.0, prints, release 42.0, prosite, release 20.97, ProDom, 2006.1, and smart, release 6.2). In addition, the KEGG[43], SwissProt, TrEMBL and Cog were used for annotation by BLAST software (v2.3.0) [44].

Orthologous gene identification

The annotated gene sequences of *L. elegans* along with other nine different species, including *Acanthoscurria geniculata* (GCA_000661875.1), *Argiope bruennichi* (GCA_015342795.1), *Parasteatoda tepidariorum* (GCF_000365465.2), *Trichonephila antipodiana* (GigaDB), *Trichonephila clavipes* (GCA_002102615.1), *Centruroides sculpturatus* (GCF_000671375.1), *Stegodyphus dumicola* (GCF_010614865.1), *Stegodyphus mimosarum* (GCA_000611955.2) and *Ixodes scapularis* (GCF_016920785.1), were used to identify the orthologous genes using OrthoMCL software (v2.0.9) [45] and default parameters. The longest transcript of each gene in these species was chosen to run the reciprocal BLAST, and the pairwise relationship was calculated. Then, the single-copy genes in these species were identified for further analysis.

Phylogenetic analysis and divergence time estimation

The phylogenetic relationships and divergence time between the 10 test species (*L. elegans*, *A. geniculata*, *A. bruennichi*, *P. tepidariorum*, *T. antipodiana*, *T. clavipes*, *C. sculpturatus*, *S. dumicola*, *S. mimosarum* and *I. scapularis*) were analyzed using the previously identified single-copy genes. All the single-copy genes in each species were connected into one super

gene. Maximum likelihood-based phylogenetic analysis was conducted using RAxML (v8.2.10) [46], and the parameters were set as follows: raxmlHPC-PTHREADS -m PROTGAMMAAUTO -N 100 -p 12345 -o I. scapularus -# 100. Then, the MCMCtree program in the PAML package (v4.8) [36] was used for divergence time calculation. All of the fossil records were downloaded from the TIMETREE database (http://www.timetree.org) for result calibration.

Relative evolution rate calculation

The relative evolution rate of these 10 species was analyzed by LINTRE software [47] via the tpcv model. We used *L. elegans* as the reference and *I. scapularus* as the outgroup species to calculate the relative evolution rate of the other species. Tajima's test in MEGA software (V10) [48] was also used for relative evolution rate calculation, with *L. elegans* as the reference and *I. scapularus* as the outgroup species.

Gene family expansion and contraction

Using the results of the divergence time and the gene pairwise relationships, the gene family expansion and contraction were determined by CAFÉ (v3.1) [49]. The gene family expansion and contraction number of each node and that of each species were calculated, and the genes of the *L. elegans* expansion or contraction families were extracted for GO/KEGG enrichment analysis. The GO enrichment was performed by Enrich GO, and KEGG analysis was performed by using R scripts [50, 51].

Positive selection analysis

To identify the potential positively selected genes (PSGs) and the genes with positively selected sites, all of the single-copy genes in *L. elegans*, *A. bruennichi*, *P. tepidariorum*, *T.*

antipodiana and *T. clavipes* were used for this analysis using Codeml in the PAML package (v4.8) [36]. The P value was calculated using the chi square table (chi2) in PAML.

Toxin gene analysis

To locate the toxin genes in the genome of both *L. elegans* and all other related species, we investigated and downloaded toxin sequences from the ArachnoServer 3.0 database [52] including four families, namely the CRISP family, the ICK family, the Latrodectin family and the Latrotoxin family. Blastp was then applied to search the candidate Latrotoxin genes against protein sequences of all associated species using '-outfmt 7 -evalue 1e-5' as the key parameters. Clustalw2 was used to perform the multiple sequence alignment to align all the protein gene sets. We then constructed the phylogeny of the Latrotoxins gene set using maximum likelihood methods in the RAxML software (v8.2.10) [46], in which raxmlHPC-PTHREADS-AVX was used as the main model with 100 bootstrap replicates, the Latrotoxin gene of *I. scapularis* was specified as the outgroup, and the key parameters were set as '-n orthology -m PROTGAMMAAUTO -f a -x 12345 -N 100 -p 12345 -o *I. scapularis*_Latrotoxins_XP_040064028.1.' Finally, the phylogenetic tree was visualized in iTOL software (http://itol.embl.de). We calculated the Ka/Ks value for each pair of genes separately using KaKs Calculator software (v2.0) [53]. ParaAT [54] was applied to achieve multiple protein-coding DNA alignments using '-m muscle -p proc -f axt' as key parameters. All merged values were plotted and grouped by species. Specifically, the toxin gene distribution of *L. elegans* was plotted by MG2C software (http://mg2c.iask.in/mg2c_v2.1/).

Analysis of spidroins

To annotate the various types of spisilk protein spidroins (MaSp, MiSp, TuSp, AcSp, Flag,

AgSp and PySp) of *L. elegans*, we initially downloaded the amino acid sequences of spidroins predicted from the *L. hesperus*, *T. clavipes*, *A. bruennichi* and *A. ventricosus* sequences obtained from the NCBI database (ABR68855.1, ABR68856.1, ARA91152.1, ARA91182.1, AWK58725.1, AFP57565.1, AFX83557.1, AAY28931.1, ACV41934.1, PRD24320.1, PRD20448.1, PRD23654.1, PRD24510.1, PRD30268.1, PRD24772.1, GFY36469.1, PRD26655.1, PRD23989.1, GFY34959.1, PRD26201.1, PRD35275.1, GFY35027.1, ADK92884.1, AFN54363.1, AGB35874.1, GBM54680.1, GBN00528.1, GBN00527.1, GBN25680.1, AFV31615.1, GBM96188.1, GBN20389.1, GBN20387.1, GBL96802.1, GBL96803.1, GBN70256.1, AUH99620.1, QKE59598.1, QKE59599.1, QBA85221.1, GBN88500.1). Using the seven different types of spider silk genes collected above as queries, we searched the annotated proteins of *L. elegans* by using the blastp with the cut-off of e-value less than 10^{-5}. The retrieved sequences of *L. elegans* were then manually filtered to remove missense mutation sequences according to the conserved domains of the spidroin sequence. These spidroin sequences were further verified by blastp against the NCBI non-redundant protein database (ftp://ftp.ncbi.nih.gov/blast/db). The chromosome distributions of spidroin were plotted by MG2C software. Sequences were aligned using MEGA software (V10) and further reorganized as categories for further research: complete, internal gap, N-terminal, C-terminal, and repetitive sequence.

Results and Discussion

Genome assembly

We sequenced the genome of *L. elegans* with a combination of third-generation sequencing,

second-generation sequencing and Hi-C sequencing. We first generated 66.49 Gb Illumina short-insert-size reads with almost 38.20X depth (**Table S1**). The 21-mer analysis showed that the genome size of *L. elegans* is ~1,74 Gb (**Figure S1**). In total, 106.80 G filtered Nanopore reads (N50 is 24.01 Kb, ~61.37-fold of the genome) were obtained (**Table S2**). The Nanopore filtered reads were assembled into contigs and further assembled into chromosomes using Hi-C reads (77.58 Gb, ~44.57-fold of the genome) (**Table S3**). We obtained a 1.57 Gb genome assembly with contig N50 of 4.34Mb and scaffold N50 of 114.31 Mb (**Table S4**). A total of 14 chromosomes were assembled with lengths ranging from 70.40 Mb to 133.92 Mb (**Figure 1A**, **Table S5**). To validate the completeness and accuracy of the *L. elegans*' genome, assembled transcripts mapping ratio, short reads mapping ratio and Benchmarking Universal Single-Copy Orthologs (BUSCO, v5.2.2) were used in the analysis. All the assembled transcripts were aligned to the genome, and 77,191 of 85,772 (90.00%) transcripts can be found in the assembled genome (**Tables S6–S8**). We aligned all the filtered short reads to the assembled genome, and more than 357.88 million reads (99.28%) could be mapped to the genome (**Table S9**). We also found that 251 of 255 (98.4%) and 930 of 954 (97.5%) core eukaryote and metazoan genes were successfully identified in the genome, respectively (**Table S10**), and this assembly quality was comparable with that of the close-related species (**Table S11**). We also identified the Hox genes in all 10 species (*A. geniculata*, *S. mimosarum*, *T. clavipes*, *A. bruennichi*, *P. tepidariorum*, *C. sculpturatus*, *S. dumicola*, *I. scapularus*, *T. antipodiana* and *L. elegans*). These results showed that *L. elegans* has two hox gene clusters. These two clusters are complete and continuous, which is comparable to other related species (**Figure S2**). These results indicate that the integrity and accuracy of the assembled genome

are good.

Genome annotation

Both tandem repeats and TEs were annotated in the *L. elegans* genome, and a total of ~506.09 Mb repeat sequences were identified that accounted for 32.30% of the assembled genome (**Table S12**). For TEs, there were 9.69% of DNA (151.78 Mb), 4.50% of LINE (70.53 Mb), 2.48% of LTR (38.89 Mb) and 1.09% of SINE (17.15 Mb) (**Table S13**). For protein-coding genes, 20,167 genes were annotated with 81.03% of the genes that had homolog genes in the public database (**Table S14**). These genes showed high similarity with related species in gene length distribution, CDS length distribution, exon length distribution and exon number distribution (**Figure S3**). The basic genome statistics of the *L. elegans* genome, including gene density, tandem repeat, LTR, LINE, SINE, DNA TEs and GC content, are shown in **Figure 1B**. We checked the synteny block between *L. elegans* and other species of Arachnida (*S. dumicola*, *P. tepidariorum* and *T. clavipes*, **Figure 1C–1E**). The results showed that *L. elegans* has a good genome synteny relationship with these species.

Phylogenetic relationship of *L. elegans* and other related species

To compare the genomics of *L. elegans* with other species, we identified the orthologous/paralogous genes among these species. A total of 28,587 gene families were clustered in these 10 species, and 156 single-copy genes were identified. The phylogenetic relationship of these 10 species was determined using the amino acid and nucleotide acid sequences of CDS, and the fourfold degenerate synonymous site (4dTV) of the single-copy genes was concatenated into a super-gene in each species. Each method showed the same phylogenetic relationship with high bootstrap results (**Figures S4–S6**). *L. elegans* is closely

related to *P. tepidariorum*, both of which are Theridiids. A calculation of the estimated divergence time indicated that *L. elegans* and *P. tepidariorum* diverged ~73.0 million years ago (Mya) (**Figure 2A**).

TE insertion history of Arachnida

We checked the types of TEs and the TE insertion time of all 10 species and found that the TE contents are significantly different. In the Theridiidae, including *L. elegans* and *P. tepidariorum*, the TE insertion times are concentrated at 20–35 million years ago. However, the TE insertion times of other species are much older than that of the Theridiidae. Besides, the insertion times of *L. elegans* (~35 million years) and *P. tepidariorum* (~20 million years) are much more recent than the divergence between two species, which suggests that the TE insertion event may have happened after their divergence (**Figure 2B**).

Gene family expansion and contraction of *L. elegans*

The gene family expansion and contraction were both calculated in each species and nodes for *L. elegans*, we identified 193 expanded gene families and 290 contracted gene families (P < 0.05) (**Figure 2A**). To investigate the function of the expanded gene families, we employed GO and KEGG enrichment analyses (**Tables S15** and **S16**). Among the GO terms of biological process, the expanded gene families were mainly enriched in biological regulation (*P*-value = 2.5279E-10) and response to stimulus (*P*-value = 1.6236E-09), especially in the regulation of cellular process (GO: 0009987, GO: 0050794), regulation of biological process (GO: 0008150, GO: 0050789) and signal transduction (GO: 0023052, GO: 0007165, GO: 0 035556). For the molecular function terms, the expanded families were enriched in hydrolase activity (GO: 0016787, *P*-value = 2.4131E-03), hydrolase activity on ester bonds (GO:

0016788, P-value = 7.1497E-08), carboxylic ester hydrolase activity (GO: 0052689, P-value = 9.9185E-11) and cholinesterase activity (GO: 0004104, P-value = 2.9828E-15); the aforementioned terms are of the subordinative relationships, relatively. The results indicated that these ontologies may be related to the specific characteristics of *L. elegans* compared with other spiders, especially in the wide distribution of venom in the whole body and the strong adaptability to the environment.

Analysis of homologs

In all of the spiders studied, except for *A. geniculata*, the number of unique paralogs and unclustered genes was relatively small. This means that most of the genes have orthologous genes. *A. geniculata* is an ancient spider belonging to Theraphosidae. It has a large genome size (7.18 Gb) compared with the other spiders (1.45–2.74 Gb) and has relatively more unique paralogs and unclustered genes. The two outgroup arachnids, i.e., *C. sculpturatus* and *I. scapularus*, also have more unique paralogs and unclustered genes than these spiders (**Figure 2C**).

At the family level, the Theraphosidae is the most specific family of arachnida with 4,915 specific genes. The Theridiidae is most closely related to the Araneidae (10,128 common gene families) and Nephilidae (11,507 common gene families) (**Figure 2D**). *L. elegans* has 2,166 specific gene families compared with three other species and is larger than the others (*P. tepidariorum*: 1031, *A. bruennichi*: 1365, *T. antipodiana*: 1085). This suggests that *L. elegans* may have more unique characters than the other species (**Figure 2E**).

Relative evolution rate of species

Species in different environments may face different selection pressures, and the relative rate

of evolution can reflect this. The relative evolution rate results showed that *L. elegans* had the fastest evolution rate among these species and suggested that it has experienced strong selection pressure. Interestingly, *A. geniculata* had the slowest evolution rate, suggesting that this original group of arachnida had relatively less selection pressure in their habitats (**Figure S7; Tables S17–S18**).

Positively selected genes

Using five relatively closely related species of orb-web weaving spiders belonging to the Araneoidea, i.e., *L. elegans*, *P. tepidariorum*, *A. bruennichi*, *T. clavipes* and *T. antipodiana*, we identified eight positively selected genes and 348 genes with positively selected sites in *L. elegans* (**Table S19**). In these genes, *lhx9* was the only gene related to gonadal development. The structures of this gene in these species were constructed, and *L. elegans* was the most unique of all species (**Figure S8**). These results indicated that the gonadal development of *L. elegans* may differ from that of the other species. The CaMKI gene belongs to calcium/calmodulin-dependent protein kinase family, and the other gene, CaMKII, is associated with OA signaling, which may affect Ca2+ signaling or adjust intracellular cAMP levels in vivo. In the spider *Cupiennius salei*, CaMKII may also be a downstream modulator of OA signaling in spider VS-3 neurons[55], which is related to cell excitability.

Venom gene analysis

The venoms of *Latrodectus* spp. are famous for their potency and ability to cause extreme and long-lasting pain. Based on genome-wide comparative analysis, we identified four major spider venom family proteins: CRISP family, ICK family, Latrodectin family and Latrotoxin family. The severe symptoms of *Latrodectus* envenomation are largely attributed to

latrotoxins. Consistently, we found that, compared to other spiders, the number of genes in the latrotoxin family of *L. elegans* and the house spider *P. tepidariorum* has undergone lineage-specific expansion. The number of latrotoxin genes found in *P. tepidariorum* and *L. elegans* was 30 and 50, respectively, which are more than that found in other spiders (1–12) (**Figure 3A**). We also identified six toxins from the CRISP family and two from the ICK family in *L. elegans* respectively. The Latrotoxin and CRISP families are both ancient and relatively conserved toxins which exist in all the test species of Arachnida. Toxins from the ICK family seem unique to the Araneoidea (**Figure 3A**). Genic loci of all these venom toxins were mapped on 11 chromosome scaffolds (Chromosomes 2, 3, 5, 7, 8, 9, 10, 11, 12, 13 and scaffold 39). The majority of latrotoxin genes were located on Chromosome 11, and there was a remarkable tandem duplication of latrotoxin genes on Chromosome 11 (**Figure 3B**). Phylogenetic analysis showed that the latrotoxins experienced substantial gene duplication and diversification in the two Theridiidae spiders, including *L. elegans* and *P. tepidariorum*, and that latrotoxins of *L. elegans* in the clade of latest expansion were mostly located on Chromosome 11 (**Figure 3C**). We analyzed the nucleotide substitutions (Ks) of latrotoxin genes in all species of spiders and found that *L. elegans* latrotoxin genes had higher Ka/Ks ratios compared to those in other species, suggesting their rapid evolution (**Figure 3D**).

Spidroin gene analysis

A female cob-weaving spider can have up to seven morphologically differentiated types of silk glands, each of which can produce a silk protein, namely spidroin. The classes of spidroins include major ampullate spidroin (MaSp), minor ampullate spidroin (MiSp), flagelliform spidroin (Flag), aggregate spidroin (AgSp), aciniform spidroin (AcSp),

tubuliform spidroin (TuSp) and pyriform spidroin (PySp). We used the same method to identify the spidroin of *L. elegans* and found six unique annotated genes for *MaSp*, eight for *MiSp*, two for *Flag*, five for *AgSp*, two for *PySp*, two for *AcSp* and one for *TuSp* **(Figure 4A)**. It is notable that *L. elegans* has relatively more *Misp* genes. MiSp is mainly used for inelastic temporary spirals during web building. The diverse Misps in *L. elegans* might provide advantages for its style of cob-web netting. All MiSps were clustered on Chromosome 14, suggesting that they may have diversified via tandem duplication **(Figure 4B)**. Other multi-copy spidroin genes such as *MaSp*, *AgSp*, *PySp* and *AcSp* were also distributed in clusters on Chromosome 11, 5, 6 and 12, respectively, suggesting that tandem duplication is the main type of duplication of spidron. The two *Flag* genes, however, are located on Chromosomes 6 and 7 **(Figure 4B).**

Conclusion

Latrodectus elegans has an extraordinarily neurotoxic venom. We used Illumina short reads, Nanopore long reads and Hi-C data to assemble a 1.57 Gb chromosome-level large genome of L. elegans. This represents the first reference genome of a *Latrodectus* species. The high-quality *L. elegans* assembled genome could be a useful resource for spider research and provides insights into spider gene diversity and speciation. Annotation of the L. elegans genome assembly identified that repetitive sequences account for 506.09 Mb (32.30%) and 20,167 protein-coding genes. The evolution rate of *L. elegans* appears to have been rapid, which suggests that this species has undergone strong selection pressure. Based on genome-wide comparative analysis, we identified 26 spider silk proteins and 39 toxin proteins.

Phylogenetic analysis also showed that the latrotoxins experienced substantial gene duplication and diversification in the two Theridiidae spiders studied (*L. elegans* and *P. tepidariorum*). Analysis of the nucleotide substitutions (Ks) of latrotoxin genes showed that *L. elegans* latrotoxin genes had higher Ka/Ks ratios compared with those in other species, suggesting rapid evolution. Analysis of the *L. elegans* genome reveals the genetic mechanisms of this species and increases understanding of the silk protein genes and toxin genes of spiders. The mechanical properties of black widow spider silk have attracted the attention of scientists. Spider venom and silk have potential applications in the military industry, medicine and in biological insecticides.

Availability of Supporting Data and Materials

All raw sequencing data and the genome assembly of *L. elegans* underlying this article are available at the NCBI and can be accessed with Bioproject ID PRJNA745004.

Additional Files

Table S1. The statistics of sequencing reads on Illumina platform.

Table S2. The statistics of sequencing reads on Nanopore platform.

Table S3. The statistics of Hi-C sequencing reads.

Table S4. The statistics of the polished genome and chromosome-level genome.

Table S5. Statistics of the assembled chromosome-level genome via 3D de novo assembly software.

Table S6. The statistics of RNA sequencing reads on Illumina platform.

Table S7. The statistics of the assembled transcripts by Bridger of 5 organs/tissues.

Table S8. The statistics of the transcripts mapping ratio on the assembled genome.

Table S9. The statistics of the short reads mapping ratio on the assembled genome.

Table S10. The quality evaluation of assembled genome by busco software.

Table S11. Comparison of all the released testudines genomes with our chromosome-level genome.

Table S12. The statistics of the annotated repeat sequences in our assembled genome.

Table S13. The statistics of the annotated repeat sequences in our assembled genome by *de novo* prediction.

Table S14. The functional annotation of the predicted protein-coding genes.

Table S15. Relative evolution rate among these species by LINTRE software.

Table S16. Relative evolution rate among these species by MEGA software.

Table S17. GO enrichment of the expanded gene families in *L. elegans* analyzed by CAFE.

Table S18. KEGG enrichment of the expanded gene families in *L. elegans* analyzed by CAFE.

Table S19. Statistics of positively selected genes of *L. elegans*.

Table S20. GO enrichment of the genes with positively selected sites in *L. elegans*.

Table S21. KEGG enrichment of the genes with positively selected sites in *L. elegans*.

Figure S1. 21-mer analysis of *L. elegans* genome.

Figure S2. Annotation and comparison of the Hox clusters among these 10 species.

Figure S3. Distribution of gene parameters in various species.

Figure S4. Phylogenetic relationship among the 10 species inferred by the amino acid

sequences of the single-copy genes.

Figure S5. Phylogenetic relationship among the 10 species inferred by the nucleotide acid sequences of the single-copy genes.

Figure S6. Phylogenetic relationship among the 10 species inferred by the 4dTV data of the single-copy genes.

Figure S7. Relative evolutionary rate of species.

Figure S8. Gene structure of lhx9 in these species.

Competing interests

The authors declare that they have no competing interests.

Authors' Contributions

H.X. and Y.R. conceived and designed the investigation. K. Z., L.G. and H. H. performed field and laboratory work. Z. W. assembled the genome. H. L. performed the Hi-C scaffold. Y. R., K.S.Z, and L. G. analyzed the data. H. X., K. S. and L. G. contributed materials and reagents. Y. R. and K.S.Z. wrote the paper. H.X and Y.R. revised the manuscript. All the authors read and approved the final manuscript.

Acknowledgements

This work was supported by the National Natural Science Foundation of China (32070411，32000383), a grant from the Natural Science Foundation of Guangdong Province, China (2019A1515011012) and the Laboratory of Lingnan Modern Agriculture Project (NZ2021019).

References

1. Slater GS and Birney E. Automated generation of heuristics for biological sequence comparison. Bmc Bioinformatics. 2005;6 doi:Artn 31 10.1186/1471-2105-6-31.
2. Fan Z, Yuan T, Liu P, Wang LY, Jin JF, Zhang F, et al. A chromosome-level genome of the spider Trichonephila antipodiana reveals the genetic basis of its polyphagy and evidence of an ancient whole-genome duplication event. Gigascience. 2021;10 3 doi:ARTN giab016 10.1093/gigascience/giab016.
3. Wirta HK, Weingartner E, Hamback PA and Roslin T. Extensive niche overlap among the dominant arthropod predators of the High Arctic. Basic Appl Ecol. 2015;16 1:86-92. doi:10.1016/j.baae.2014.11.003.
4. Moulder BC and Reichle DE. Significance of Spider Predation in Energy Dynamics of Forest-Floor Arthropod Communities. Ecol Monogr. 1972;42 4:473-98. doi:Doi 10.2307/1942168.
5. Dimitrov D, Lopardo L, Giribet G, Arnedo MA, Alvarez-Padilla F and Hormiga G. Tangled in a sparse spider web: single origin of orb weavers and their spinning work unravelled by denser taxonomic sampling. P Roy Soc B-Biol Sci. 2012;279 1732:1341-50. doi:10.1098/rspb.2011.2011.
6. Wright S and Goodacre SL. Evidence for antimicrobial activity associated with common house spider silk. BMC Res Notes. 2012;5:326-. doi:10.1186/1756-0500-5-326.

7. Altman GH, Diaz F, Jakuba C, Calabro T, Horan RL, Chen JS, et al. Silk-based biomaterials. Biomaterials. 2003;24 3:401-16. doi:Pii S0142-9612(02)00353-8 Doi 10.1016/S0142-9612(02)00353-8.

8. Kluge JA, Rabotyagova U, Leisk GG and Kaplan DL. Spider silks and their applications. Trends Biotechnol. 2008;26 5:244-51. doi:10.1016/j.tibtech.2008.02.006.

9. King GF and Hardy MC. Spider-Venom Peptides: Structure, Pharmacology, and Potential for Control of Insect Pests. Annu Rev Entomol. 2013;58:475-96. doi:10.1146/annurev-ento-120811-153650.

10. Escoubas P, Diochot S and Corzo G. Structure and pharmacology of spider venom neurotoxins. Biochimie. 2000;82 9-10:893-907. doi:Doi 10.1016/S0300-9084(00)01166-4.

11. Garb JE, Gonzalez A and Gillespie RG. The black widow spider genus Latrodectus (Araneae : Theridiidae): phylogeny, biogeography, and invasion history. Mol Phylogenet Evol. 2004;31 3:1127-42. doi:10.1016/j.ympev.2003.10.012.

12. Jelinek GA. Widow spider envenomation (latrodectism): A worldwide problem. Wild Environ Med. 1997;8 4:226-31. doi:Doi 10.1580/1080-6032(1997)008[0226:Wselaw]2.3.Co;2.

13. Wang XC, Tang XC, Xu DH and Yu DAM. Molecular basis and mechanism underlying the insecticidal activity of venoms and toxins from Latrodectus spiders. Pest Manag Sci. 2019;75 2:318-23. doi:10.1002/ps.5206.

14. Yan S and Wang XC. Recent Advances in Research on Widow Spider Venoms and Toxins. Toxins. 2015;7 12:5055-67. doi:10.3390/toxins7124862.

15. Akhunov AA, Golubenko Z, Abdurashidova NA, Mustakimova EC, Ibragimov FA and Mackessy S. Comparative biochemistry of the physiologically active components of venom,

hemolymph, and eggs of the karakurt spider (Latrodectus tredecimguttatus). Chem Nat Compd+. 2001;37 6:562-5. doi:Doi 10.1023/A:1014829218721.
16. D.C. B and F.E. R. A poison from the eggs and spiderlings of the black widow spider. 1971.
17. C. BD, E. RF and A. D. Preliminary studies on the toxicity of black widow spider eggs. 1971;9 4:393-402.
18. Gendreau KL, Haney RA, Schwager EE, Wierschin T, Stanke M, Richards S, et al. House spider genome uncovers evolutionary shifts in the diversity and expression of black widow venom proteins associated with extreme toxicity. Bmc Genomics. 2017;18 doi:ARTN 178 10.1186/s12864-017-3551-7.
19. Argintean S, Chen J, Kim M and Moore AMF. Resilient silk captures prey in black widow cobwebs. Appl Phys a-Mater. 2006;82 2:235-41. doi:10.1007/s00339-005-3430-y.
20. Berger CA, Brewer MS, Kono N, Nakamura H, Arakawa K, Kennedy SR, et al. Shifts in morphology, gene expression, and selection underlie web loss in Hawaiian Tetragnatha spiders. Bmc Ecol Evol. 2021;21 1 doi:ARTN 48 10.1186/s12862-021-01779-9.
21. Stellwagen SD and Renberg RL. Toward Spider Glue: Long Read Scaffolding for Extreme Length and Repetitious Silk Family Genes AgSp1 and AgSp2 with Insights into Functional Adaptation. G3-Genes Genom Genet. 2019;9 6:1909-19. doi:10.1534/g3.119.400065.
22. Blasingame E, Tuton-Blasingame T, Larkin L, Falick AM, Zhao L, Fong J, et al. Pyriform Spidroin 1, a Novel Member of the Silk Gene Family That Anchors Dragline Silk Fibers in Attachment Discs of the Black Widow Spider, Latrodectus hesperus. J Biol Chem. 2009;284 42:29097-108. doi:10.1074/jbc.M109.021378.
23. Correa-Garhwal SM, Chaw RC, Clarke TH, Ayoub NA and Hayashi CY. Silk gene expression

of theridiid spiders: implications for male-specific silk use. Zoology. 2017;122:107-14. doi:10.1016/j.zool.2017.04.003.

24. Sheffer MM, Hoppe A, Krehenwinkel H, Uhl G, Kuss AW, Jensen L, et al. Chromosome-level reference genome of the European wasp spider Argiope bruennichi: a resource for studies on range expansion and evolutionary adaptation. Gigascience. 2021;10 1 doi:ARTN giaa148 10.1093/gigascience/giaa148.

25. Jain M, Olsen HE, Paten B and Akeson M. The Oxford Nanopore MinION: delivery of nanopore sequencing to the genomics community (vol 17, 239, 2016). Genome Biol. 2016;17 doi:ARTN 256 10.1186/s13059-016-1122-x.

26. Eagen KP. Principles of Chromosome Architecture Revealed by Hi-C. Trends Biochem Sci. 2018;43 6:469-78. doi:10.1016/j.tibs.2018.03.006.

27. Vurture GW, Sedlazeck FJ, Nattestad M, Underwood CJ, Fang H, Gurtowski J, et al. GenomeScope: fast reference-free genome profiling from short reads. Bioinformatics. 2017;33 14:2202-4. doi:10.1093/bioinformatics/btx153 %J Bioinformatics.

28. Hu J: https://github.com/Nextomics/NextDenovo (2020).

29. Dudchenko O, Batra SS, Omer AD, Nyquist SK, Hoeger M, Durand NC, et al. De novo assembly of the Aedes aegypti genome using Hi-C yields chromosome-length scaffolds. Science. 2017;356 6333:92-5. doi:10.1126/science.aal3327.

30. Simao FA, Waterhouse RM, Ioannidis P, Kriventseva EV and Zdobnov EM. BUSCO: assessing genome assembly and annotation completeness with single-copy orthologs. Bioinformatics. 2015;31 19:3210-2. doi:10.1093/bioinformatics/btv351.

31. Li H and Durbin R. Fast and accurate short read alignment with Burrows-Wheeler transform.

Bioinformatics. 2009;25 14:1754-60. doi:10.1093/bioinformatics/btp324.

32. Chang Z, Li GJ, Liu JT, Zhang Y, Ashby C, Liu DL, et al. Bridger: a new framework for de novo transcriptome assembly using RNA-seq data. Genome Biol. 2015;16 doi:ARTN 30 10.1186/s13059-015-0596-2.

33. Benson G. Tandem repeats finder: a program to analyze DNA sequences. Nucleic Acids Res. 1999;27 2:573-80. doi:DOI 10.1093/nar/27.2.573.

34. Bedell JA, Korf I and Gish W. MaskerAid: a performance enhancement to RepeatMasker. Bioinformatics. 2000;16 11:1040-1. doi:DOI 10.1093/bioinformatics/16.11.1040.

35. RepeatModeler: http://www.repeatmasker.org/RepeatModeler/. Accessed December 27 2019.

36. Yang ZH. PAML: a program package for phylogenetic analysis by maximum likelihood. Comput Appl Biosci. 1997;13 5:555-6.

37. Stanke M and Waack S. Gene prediction with a hidden Markov model and a new intron submodel. Bioinformatics. 2003;19:Ii215-Ii25. doi:10.1093/bioinformatics/btg1080.

38. Birney E and Durbin R. Using GeneWise in the Drosophila annotation experiment. Genome Res. 2000;10 4:547-8. doi:DOI 10.1101/gr.10.4.547.

39. Kent WJ. BLAT - The BLAST-like alignment tool. Genome Res. 2002;12 4:656-64. doi:10.1101/gr.229202.

40. Haas BJ, Delcher AL, Mount SM, Wortman JR, Smith RK, Hannick LI, et al. Improving the Arabidopsis genome annotation using maximal transcript alignment assemblies. Nucleic Acids Res. 2003;31 19:5654-66. doi:10.1093/nar/gkg770.

41. Haas BJ, Salzberg SL, Zhu W, Pertea M, Allen JE, Orvis J, et al. Automated eukaryotic gene structure annotation using EVidenceModeler and the program to assemble spliced alignments.

Genome Biol. 2008;9 1 doi:ARTN R7 10.1186/gb-2008-9-1-r7.

42. Zdobnov EM and Apweiler R. InterProScan - an integration platform for the signature-recognition methods in InterPro. Bioinformatics. 2001;17 9:847-8. doi:DOI 10.1093/bioinformatics/17.9.847.

43. Kanehisa M, Goto S, Kawashima S and Nakaya A. The KEGG databases at GenomeNet. Nucleic Acids Res. 2002;30 1:42-6. doi:DOI 10.1093/nar/30.1.42.

44. Altschul SF, Gish W, Miller W, Myers EW and Lipman DJ. Basic Local Alignment Search Tool. J Mol Biol. 1990;215 3:403-10. doi:DOI 10.1006/jmbi.1990.9999.

45. Li L, Stoeckert CJ and Roos DS. OrthoMCL: Identification of ortholog groups for eukaryotic genomes. Genome Res. 2003;13 9:2178-89. doi:10.1101/gr.1224503.

46. Stamatakis A. RAxML version 8: a tool for phylogenetic analysis and post-analysis of large phylogenies. Bioinformatics. 2014;30 9:1312-3. doi:10.1093/bioinformatics/btu033.

47. Takezaki N, Rzhetsky A and Nei M. Phylogenetic Test of the Molecular Clock and Linearized Trees. Mol Biol Evol. 1995;12 5:823-33.

48. Kumar S, Tamura K and Nei M. Mega - Molecular Evolutionary Genetics Analysis Software for Microcomputers. Comput Appl Biosci. 1994;10 2:189-91.

49. De Bie T, Cristianini N, Demuth JP and Hahn MW. CAFE: a computational tool for the study of gene family evolution. Bioinformatics. 2006;22 10:1269-71. doi:10.1093/bioinformatics/btl097.

50. Beissbarth T and Speed TP. GOstat: find statistically overrepresented Gene Ontologies within a group of genes. Bioinformatics. 2004;20 9:1464-5. doi:10.1093/bioinformatics/bth088.

51. Huang DW, Sherman BT and Lempicki RA. Bioinformatics enrichment tools: paths toward

the comprehensive functional analysis of large gene lists. Nucleic Acids Res. 2009;37 1:1-13. doi:10.1093/nar/gkn923.

52. Wood DLA, Miljenovic T, Cai SZ, Raven RJ, Kaas Q, Escoubas P, et al. ArachnoServer: a database of protein toxins from spiders. Bmc Genomics. 2009;10 doi:Artn 375 10.1186/1471-2164-10-375.

53. Wang D, Zhang Y, Zhang Z, Zhu J and Yu J. KaKs_Calculator 2.0: A Toolkit Incorporating Gamma-Series Methods and Sliding Window Strategies. Genomics, Proteomics & Bioinformatics. 2010;8 1:77-80. doi:https://doi.org/10.1016/S1672-0229(10)60008-3.

54. Zhang Z, Xiao JF, Wu JY, Zhang HY, Liu GM, Wang XM, et al. ParaAT: A parallel tool for constructing multiple protein-coding DNA alignments. Biochem Bioph Res Co. 2012;419 4:779-81. doi:10.1016/j.bbrc.2012.02.101.

55. Torkkeli PH, Panek I and Meisner S. Ca2+/calmodulin-dependent protein kinase II mediates the octopamine-induced increase in sensitivity in spider VS-3 mechanosensory neurons. Eur J Neurosci. 2011;33 7:1186-96. doi:10.1111/j.1460-9568.2011.07624.x.

Figures

Figure 1. Genome assembly and comparative analysis of the *L. elegans*. A. Heatmap of chromosome interactions in *L. elegans*. **B.** Circos plot of distribution of the genomic elements in *L. elegans*. From the outer ring to the inner ring are the distributions of protein-coding genes, tandem repeats (TRs), long tandem repeats (LTRs), short/long interspersed nuclear elements (SINEs/LINEs), DNA elements and GC content, respectively. **C.** Genomic synteny between *S. dumicola* and *L. elegans*. **D.** Genomic synteny between *P. tepidariorum* and *L. elegans*. **E.** Genomic synteny between *T. clavipes* and *L. elegans*.

Figure 2. Comparative genomics of *L. elegans* and related species. A. Phylogenetic relationships among these species. The red dot at the node represents a fossil record that was used for the calibration of the divergence time. The blue number in each node represents its divergence time for species. The red and green numbers in each node/species represent the expanded/contracted gene families, respectively. **B.** Comparison of the insertion history of transposable elements among species. The x-axis represents the inferred insertion time (unit: million years ago) of transposable elements in the genome. The y-axis represents the total/each length of the transposable element in each species. **C.** Statistics of all orthologous/paralogous gene numbers in these species. **D.** A Venn diagram displaying the overlap in orthologous genes in different families of Arachnida. **E.** A Venn diagram displaying the overlap in orthologous genes in *L. elegans* and other three spiders.

Figure 3. Toxin genes in the Arachnida species. A. Different toxin gene numbers in these

species. **B.** The distribution of toxin genes in *L. elegans*. **C.** The phylogenetic relationship of latrotoxins genes in these species. **D.** Latrotoxin gene Ka/Ks value of these species. The latrotoxin gene in *I. scapularus* was used as the reference.

Figure 4. Spidron genes in the Arachnida species. A. Different spidron gene numbers in three spider species. **B.** The chromosome distribution of spidron genes in *L. elegans*.

Figure 1. Genome assembly and comparative analysis of the L. elegans.

Click here to access/download;Figure;Figure 1.pdf

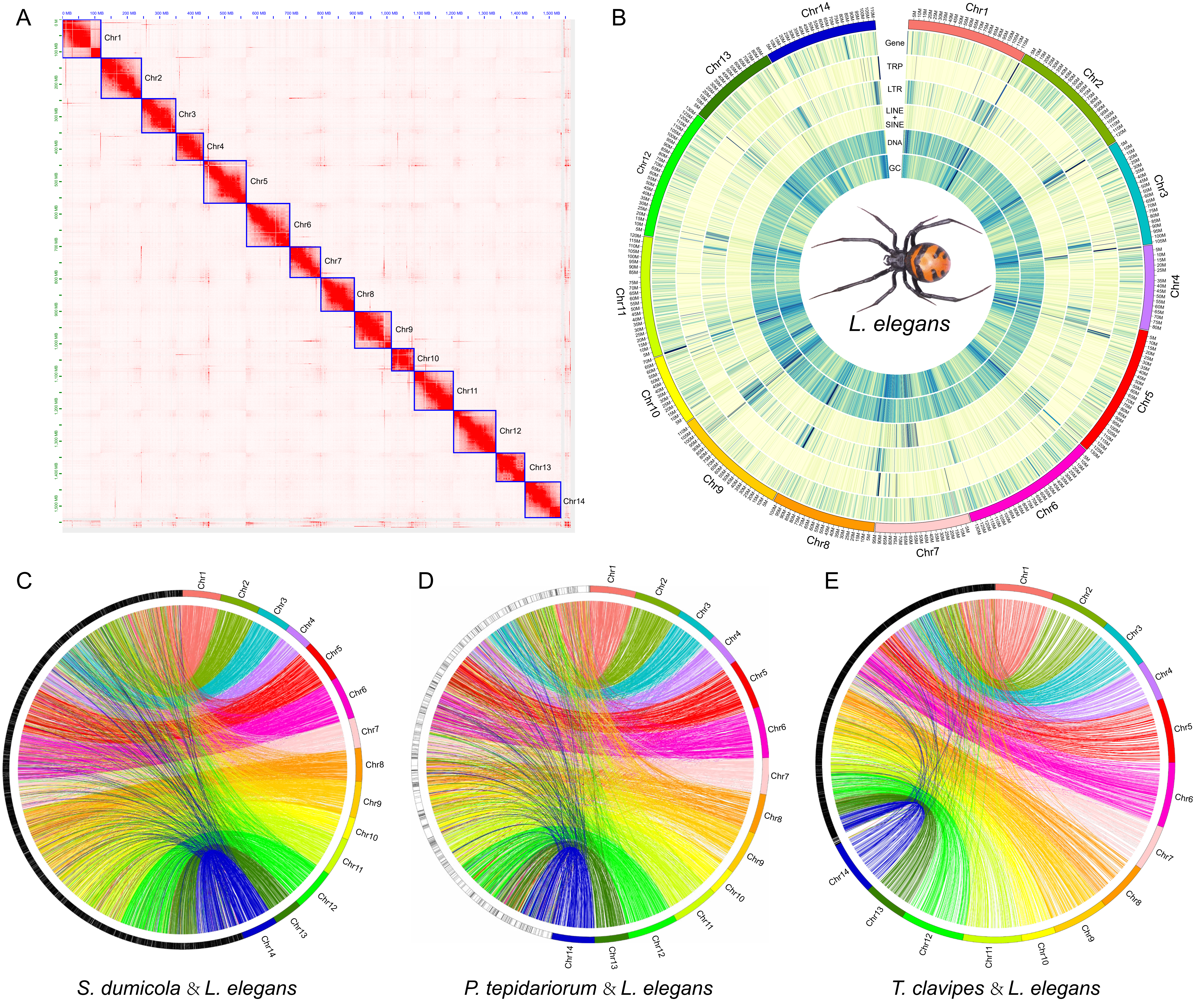

Figure 2. Comparative genomics of L. elegans and related species.

Click here to access/download;Figure;Figure 2.pdf

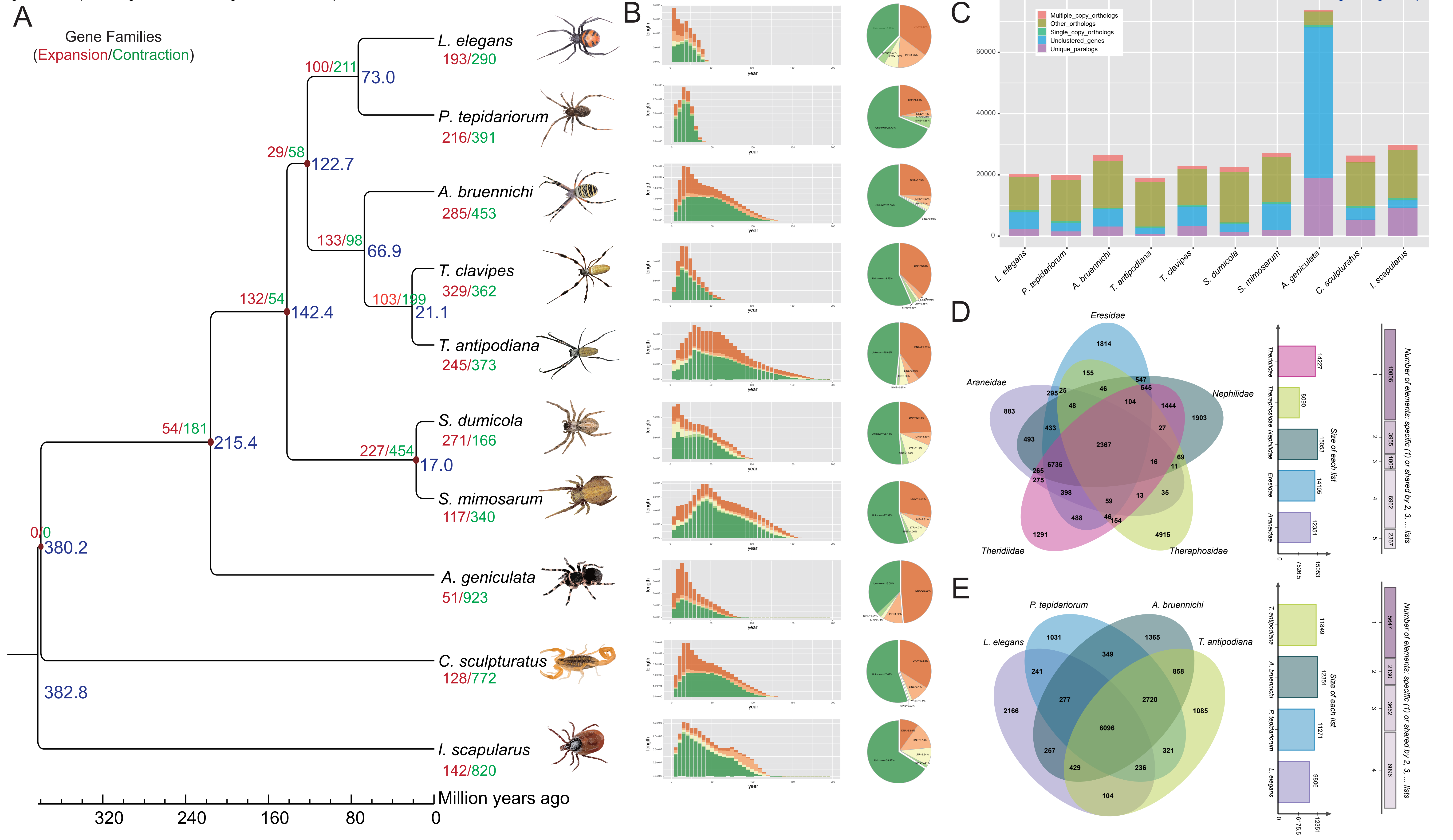

Figure 3. Toxin genes in the Arachnida species.

Click here to access/download;Figure;Figure 3.pdf

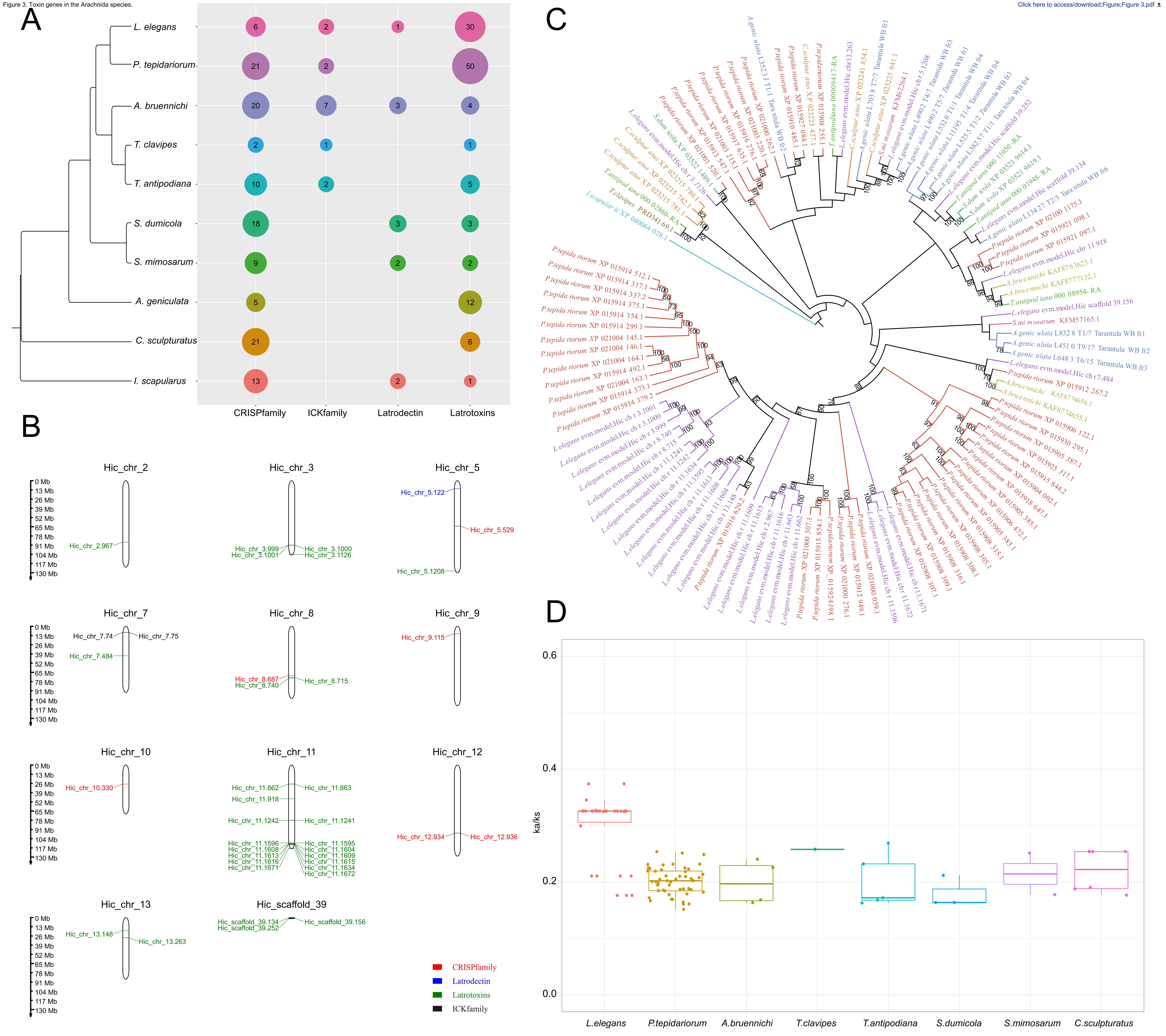

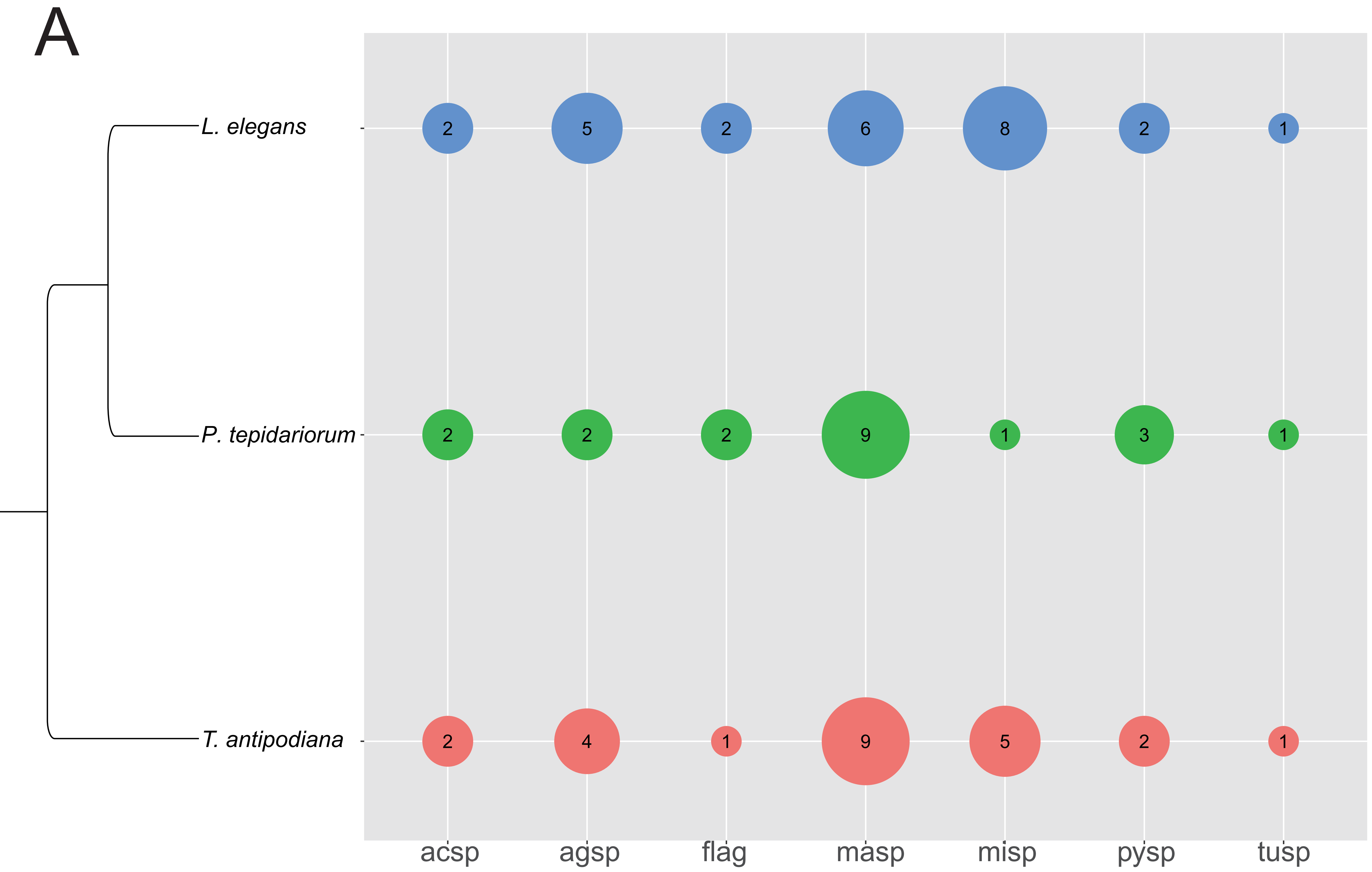
A
L. elegans
P. tepidariorum
T. antipodiana
2
5
2
6
8
2
1
2
2
2
9
1
3
1
2
4
1
9
5
2
1
acsp
agsp
flag
masp
misp
pysp
tusp

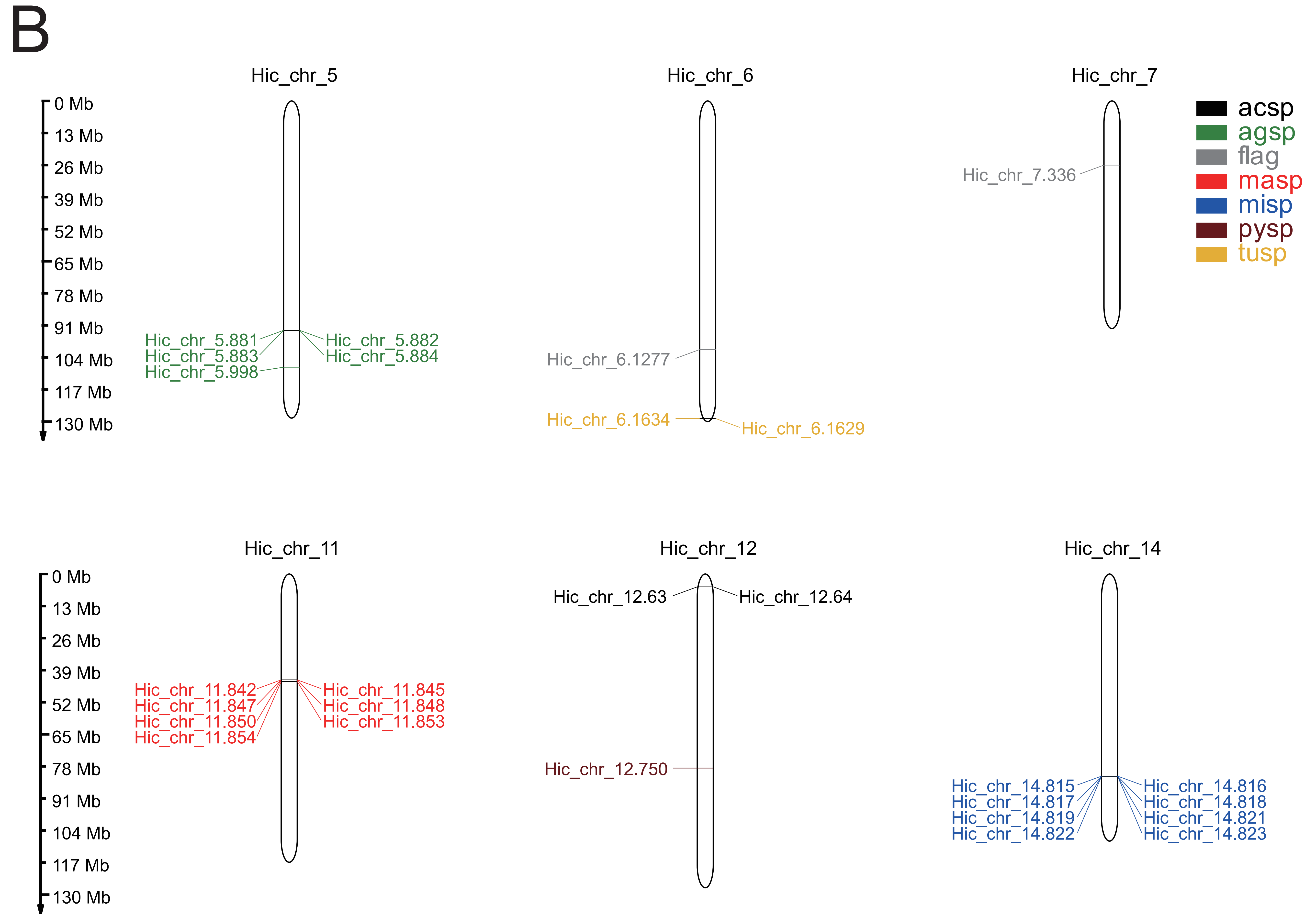
B
Hic_chr_5
Hic_chr_6
Hic_chr_7
0 Mb
13 Mb
26 Mb
39 Mb
52 Mb
65 Mb
78 Mb
91 Mb
104 Mb
117 Mb
130 Mb
acsp
agsp
flag
masp
misp
pysp
tusp
Hic_chr_5.881
Hic_chr_5.883
Hic_chr_5.998
Hic_chr_5.882
Hic_chr_5.884
Hic_chr_6.1277
Hic_chr_6.1634
Hic_chr_6.1629
Hic_chr_7.336
Hic_chr_11
Hic_chr_12
Hic_chr_14
Hic_chr_11.842
Hic_chr_11.847
Hic_chr_11.850
Hic_chr_11.854
Hic_chr_11.845
Hic_chr_11.848
Hic_chr_11.853
Hic_chr_12.63
Hic_chr_12.64
Hic_chr_12.750
Hic_chr_14.815
Hic_chr_14.817
Hic_chr_14.819
Hic_chr_14.822
Hic_chr_14.816
Hic_chr_14.818
Hic_chr_14.821
Hic_chr_14.823

Click here to access/download
Supplementary Material
spiderGonomeMSSupplementary tables & figures_20201008 - new.docx
