# Supplementary material for: Chromosome‐level genome assembly of the black widow spider Latrodectus elegans illuminates composition and evolution of venom and silk proteins
Source: Gigascience. 2022 May 25;11:giac049. doi: 10.1093/gigascience/giac049 (PMC9154082; doi:10.1093/gigascience/giac049)
Supplement: giac049_GIGA-D-21-00338_Revision_1 [file giac049_giga-d-21-00338_revision_1.pdf]

GigaScience

Chromosome-level genome assembly of the black widow spider Latrodectus elegans illuminates composition and evolution of venom and silk proteins

--Manuscript Draft--

**Manuscript Number:**	GIGA-D-21-00338R1
**Full Title:**	Chromosome-level genome assembly of the black widow spider Latrodectus elegans illuminates composition and evolution of venom and silk proteins
**Article Type:**	Data Note
**Funding Information:**	National Natural Science Foundation of China (32070411 , 32000383) — Prof. Hui Xiang<br>Natural Science Foundation of Guangdong Province (2019A1515011012) — Prof. Hui Xiang<br>Laboratory of Lingnan Modern Agriculture Project (NZ2021019) — Prof. Hui Xiang
**Abstract:**	Background: Black widow spider has both extraordinarily neurotoxic venom and three-dimensional cob-webs composing of diverse types of silk. However, high-quality reference genome for black widow spider was still unavailable, which hindered deep understanding and application of the valuable biomass.<br>Findings: We assembled the L. elegans genome and the genome size is 1.57 Gb with contig N50 of 4.34Mb and scaffold N50 of 114.31 Mb. Hi-C scaffolding assigned 98.08% of the genome to 14 pseudo-chromosomes, and with BUSCO (Benchmarking Universal Single-Copy Orthologs) completeness analysis revealed that 98.4% of the core eukaryotic genes were completely present in this genome. Annotation of this genome identified that repetitive sequences account for 506.09 Mb (32.30%) and 20,167 protein-coding genes and specifically, we identified 55 toxin genes and 26 spidroins and provide preliminary analysis their composition and evolution.<br>Conclusions: We present the first chromosome-level genome assembly of a black widow spider and provided substantial toxin and spidroins gene resources. These high-qualified genomic data add valuable resources from a representative spider group and contribute to deep exploration on spider genome evolution, especially in terms of the important issues on diversification of venom and web-weaving pattern. The sequence data are also first-hand templates for further application of the spider biomass.
**Corresponding Author:**	Hui Xiang, PH.D<br>South China Normal University<br>Guangzhou, CHINA
**Corresponding Author Secondary Information:**	
**Corresponding Author's Institution:**	South China Normal University
**Corresponding Author's Secondary Institution:**	
**First Author:**	Zhongkai Wang
**First Author Secondary Information:**	
**Order of Authors:**	Zhongkai Wang
	Kesen Zhu
	Haorong Li
	Lei Gao
	Huanying Huang
	Yandong Ren

Powered by Editorial Manager® and ProduXion Manager® from Aries Systems Corporation

	Hui Xiang
**Order of Authors Secondary Information:**	
**Response to Reviewers:**	Dear Editor,<br>Thank you and the three reviewers for your time and constructive comments on our manuscript “ Chromosome-level genome assembly of the black widow spider Latrodectus elegans illuminates composition and evolution of venom and silk proteins" (GIGA-D-21-00338). These comments will greatly help improving our manuscript. Now we carefully revised our manuscript according to the reviewers’ comment and polish the grammar in our revised manuscript. We wish that the updated manuscript could fit the reviewers’ requirement. Meantime, we made the point-by-point responses to each of the comment from the editor and the reviewers, as follows: (We also attached a response letter file in our submission materal)<br><br><br>Editor:<br>Please also take a moment to check our website at https://www.editorialmanager.com/giga/ for any additional comments that were saved as attachments.<br>R: we checked the website and found no additional comments. Thank you for your kindly reminding.<br>In addition, please register any new software application in the bio.tools and SciCrunch.org databases to receive RRID (Research Resource Identification Initiative ID) and biotoolsID identifiers, and include these in your manuscript. This will facilitate tracking, reproducibility and re-use of your tool.<br>R: Thank you for your reminding. We checked all the softwares used in our manuscript and confirmed that there was no any new software used. Meantime, the electronic notebook-style methods document of these softwares were upload to the GigaDB dataset. We affirm that our tools are easy to track and reproducible.<br>Please ensure you describe additional experiments that were carried out and include a detailed rebuttal of any criticisms or requested revisions that you disagreed with.<br>Please also ensure that your revised manuscript conforms to the journal style, which can be found in the Instructions for Authors on the journal homepage. If the data and code has been modified in the revision process please be sure to update the public versions of this too.<br>R: We have ensured all the above items.<br>Besides, we suggest you find a copy editing company or friendly native English speaker to polish the grammar.<br>R: We have made our manuscript polished by a copy editing company (Letpub).<br><br>Reviewer reports:<br>Reviewer #1: The manuscript described the genome of an orb-weaver spider Latrodectus elegans with a combination of Nanopore, Illumina and Hi-C sequencing and the comparison of latrotoxin genes and spidroin genes among spiders. The authors reported the assembly, quality and analysis of the genome in details and provided enormous information, in my opinion, useful to the research community. However, I suggest a major revision before the work is published. The main concern is on the toxin identification.<br>R: Thank you very much for your overall positive comments to our manuscript and important suggestion.<br>I have one major concern related to the analysis of venom toxin genes. In Fig. 3 and related text, no ICK family gene was identified in five species including spider, scorpion and tick where ICK toxins have been reported and even functionally studied. In the other five spider species, fewer ICK genes were found (1 to 7). ICK family was considered an abundant toxin family in Arachnida and their homology is mainly dependent on the disulfide-bond instead of the primary sequence. The toxin genes were identified via blastp, which heavily relies on the sequence similarity. Similar concerns may apply to the other toxin families. I would suggest the authors redo the toxin gene analysis.<br>R: Thanks for your important comments and we are sorry for our carelessness in spider toxin identification. We investigated more on these toxins and learned that, as the reviewer stated, ICK toxins are a kind of small peptide and their homology is mainly dependent on the disulfide-bond instead of the primary sequence, unlike those proteins from CRISP family or Latrotoxin family. We then re-identified the ICK toxins through the

following approaches. Firstly, we retrieved the peptides less than 200 amino acid and have at least six cysteines constituting the NCNCNCCNCNC (N refers to any other amino acid) motif. Secondly, we used the online tool ClanTox (http://www.clantox.cs.huji.ac.il/index.php) to evaluate the toxin potential of the above candidate ICK toxins. Thirdly, we used SignalP (v5.0) to predict signal peptide of them and those without signal peptide was removed out. After further manual check, we finally identified the ICK toxins. As to Latrodectin family genes, there are rather few genes in ArachnoServer 3.0 database. We then retrieved the known sequences of Latrodectin from NCBI, then we applied the Blastp with e value cutoff of 10-5 to search the candidate homolog proteins. Then the homolog was further verified by hmmer (v3.3). In brief, all above-found sequences were merged together and applied Clustalw2 to perform the multiple sequence alignment which were then transformed into stockholm format using online toolset (https://novopro.cn/tools/format-conversion.html). The aligned sequences were used to construct the HMM profile using hmmbuild, a binary software of hmmer (v3.3). Finally, hmmsearch was applied for the peptide of each species, to find the potential domains of all species using key parameters "—domtblout and e-value of 10-5".
Based on the updated methods, we did identify more ICK toxins. We updated the related methods and the results in the revised manuscript.

Minor errors:
1. Line 36, "remarkably more MiSp". It is grossly overwritten.
R: Thanks for reminding. We removed "remarkably" to downplay the statement.
2. Line 345-346, any reference?
R: We added a reference "Bhere KV, Haney RA, Ayoub NA and Garb J E. Gene structure, regulatory control, and evolution of black widow venom latrotoxins. FEBS Lett 2014; 588(21): 3891-3897." therein. Thanks for reminding.
3. Line 368, "We used the same method". What is the method? Same to what? There is not description in the above sentences.
R: Sorry for this rather poor writing. We removed these statements since we have described the method of spidroin genes in the Methods section. We modified the statements to "We identified six unique annotated genes for MaSp, eight for MiSp, two for Flag, five for AgSp, two for PySp, two for AcSp and one for TuSp in L. elegans".
4. Many typo are present in the main text. The manuscript could be improved by carefully revising the text and reformatting the reference list.
R: We are sorry for our carelessness. We carefully revised the main test and had let a copy editing company (Letpub) polish the grammar.

Reviewer #2: In this manuscript, the authors present a high-quality genome assembly of the species Latrodectus elegans, based on nanopore reads and HI-C scaffolding. In addition, they present the most relevant characteristics of the genome and its functional annotation, as well as a phylogenetic analysis that includes other arachnids, estimates of the evolutionary rate in different lineages, and focused on the chromosomal organization and evolution of some gene families of interest, such as toxins and spidroins.

I think the work is very interesting. This new genome provides new data that will be very useful to advance our understanding of the diversification of spiders and the evolution of some of the most relevant aspects of their biology. Nevertheless, I have some important concerns about different aspects of the ms, which in my opinion must be properly addressed before considering the manuscript suitable for publication in Gigascience.
R: Thank you very much for your interest of our manuscript and the instructive comments.

First of all, I think it is necessary to improve the language. I am not a native English speaker, but I think there are many sentences that are not well punctuated and some expressions are not well constructed. I highly recommend a thorough review of the entire text.
R: We are very sorry for our poor writing. We made a carefully revision and modification in writing. In addition, we had let a copy editing company polish our manuscript, in order to further improve it.
Apart from the language itself, there are poorly constructed sentences from the point of view of biological concepts. Phrases such as "Systematic identification of complete

Powered by Editorial Manager® and ProduXion Manager® from Aries Systems Corporation

	toxin gene sequence", in line 68, or "..will aid the identification and understanding of the complete spider toxin and spider silk genes..line 76", or "..to detect the genome integrity..line 117", or "transposon elements..line 121"..or "connected into one super gene..line 169", or "all merged values were plotted and grouped by species...line 210", or "the chromosome distribution of spidroin were plotted...line 229", or "..genes that had homolog genes in the public database..line 264", or "the original group of arachnida..line 327"..etc..<br>R: Line 68: we modified it to: “Systematic identification and analysis of all the spider toxin genes with full length sequences”.<br>Line 76: we modified it to: “…will provide important resources for deciphering the spider toxin and spidroin genes”.<br>Line 117: we modified it to: “..to evaluate the genome integrity.”<br>Line 121: we modified it to: “transposable elements”.<br>Line 169: we modified the sentence to: “All the well-aligned single-copy orthologous genes in each species were concatenated to one super gene for each species.”<br>Line 210: we modified it to: “all the Ka/Ks values were plotted and grouped by species.” We also made other modifications in the context.<br>Line 229: we modified it to: “The chromosome distribution of the spidroins were then plotted ...”.<br>Line 264: we modified it to: “... genes having homologous hits in the public databases.”<br>Line 327: we modified it to “... It is the only species from the relatively ancient Mygalomorphae. The result suggests that relatively ancient spider group may have relatively less selection pressure in their habitats. However, due to its low genome quality, this result is pending further verification....”.<br>In addition, we carefully checked the whole manuscript and made intensive revisions of it.<br><br>Regarding specific contents, there are some things that need to be revised, rewritten or expanded. Here are some examples:<br>In line 137 a sentence begins with "the protein sequences of ...." and then, after the comma, continues with "... the longest transcript were chosen for further analyzes. Something is wrong here ...<br>R: We modified the statement to: “We chose the longest isoform of each gene to obtain the non-redundant protein sequences of each gene set.” We also made modifications in the context.<br>In line 143, the phrase "All the remaining genes were aligned using blastn" is not understood, what do the authors mean?<br>R: We modified the related statements to: “We chose the longest isoform of each gene to obtain the non-redundant protein sequences of each gene set. The protein sequences were used as query to search for orthologous regions in the L. elegans genome by tblastn with an e-value of 1e-5. The results were subjected to GeneWise software (v2.4.1) (Birney and Durbin 2000) to predict gene structure.”<br>Various lines. The names and identifiers of the species used in the different analyzes are unnecessarily repeated in several paragraphs.<br>R: Thanks for reminding. We carefully checked the manuscript and removed the repeated names and identifiers of the species.<br>line 150. Please, replace "homologous searching" with "sequence similarity-based searches" or similar...<br>R: Thanks. We replaced "homologous searching" with" homology-based searches".<br>Line 183. "Using ..... gene pairwise relationships". What do the authors mean by this phrase? What pairwise relationships do they use to determine the evolution of families in CAFE?<br>R: We are sorry for this vague description. However, in order to limiting genomic analysis and make the manuscript meet the requirement of “Date note” in Gigascience, we removed unnecessary genomic analysis, including analysis on gene family expansion and contraction. So this sentence was removed.<br>line 190. "To identify the potential positively selected genes (PSGs) and the genes with positively selected sites ..." I do not understand the difference between these two classes of genes.<br>R: Sorry that we didn’t describe it clear. We used different models in Codeml tool of PAML package (v4.8) to detect potential positively selected genes (PSGs) and the genes with positively selected sites. PSGs were those identified by two ratio branch model. Those not identified as PSGs, were then subjected to branch site model to detect whether they have positive selected sites. We re-wrote the method of positive

Powered by Editorial Manager® and ProduXion Manager® from Aries Systems Corporation

selection analysis: “Single-copy orthologs from the five relatively closely related species of orb-web weaving spiders, i.e., L. elegans, P. tepidariorum, A. bruennichi, T. clavipes and T. antipodiana. As well as their species tree were used to identify the potential positively selected genes (PSGs) and the genes with positively selected sites in L. elegans, using branch model and branch site model in the Codeml tool of the PAML package (v4.8) (Yang 1997) respectively. Briefly, the rate ratio (ω) of nonsynonymous to synonymous nucleotide substitutions was estimated. One ratio model was used to detect average ω across the species tree (ω0). For each gene, two ratio branch model was used to detect the ω of the appointed branch (L. elegans) to test the (ω1) and ω of all other branches (ω_background). A likelihood ratio test was performed to compare the fit of the two ratio models with the one ratio model to determine whether the gene is positively selected in the appointed branch (ω1>ω0; ω1>ω_background; ω1>1; P-value<0.05). If the gene is not positively selected, then the branch site model was used to detect amino acid sites likely to be is positively selected in the appointed branch using Bayes Empirical Bayes (BEB) analysis (Yang et al., 2005). ”
Line 229. It should say "distribution of spidroins" ...
R: Thanks and we revised it.
In material and methods, for example, much information is lacking on how or with what software some things have been done. For example, the exact location where the sample was obtained is missing, now only the province is specified (line 87). Authors do not specify where the sequencing has been done (lines 91 and 92). The software used to filter the nanopore reads is missing (lines 96 and 97). Are the Perl scripts used written specifically for the project (in house-scripts) or are part of other software (line 96)?. It is not very clear for me what the Kimura value means (line 130), is it the corrected evolutionary distance using a kimura model? and, if true, which model, 2P or 3P?). Is the r8s program really implemented in the PAML package? (line 132).
R: Line 87: the spider is collected in Binzhou county, Dali City, Yunnan province.
Lines 91 and 92: we sent the live sample to Beijing Biomarker Technologies. Co. LTD for sequencing.
Lines 96 and 97: the Perl script used for filter the Nanopore reads is written specifically for this project (in house-script). We added it in our electronic notebook-style methods document and upload to the GigaDB dataset.
Line130: Yes, Kimura value is the corrected evolutionary distance using a kimura model. We used the parseRM (https://github.com/ 4ureliek/Parsing-RepeatMasker-Outputs) to conduct the kimura-distance based analysis on TE insertion time. We checked the pipeline and confirmed that 2P model is used therein. The principle is from the reference: Chalopin, D., Naville, M., Plard, F., Galiana, D., and Volff, J.-N. (2015). Comparative analysis of transposable elements highlights mobilome diversity and evolution in vertebrates. Genome Biol. Evol. 7, 567–580.
Line 132: We are sorry for this mistake. Yes, r8s is an individual program. Given that we re-wrote the description of TE analysis (The insertion time of each TE sequence was estimated using a Kimura distance based analysis (Chalopin, Naville et al. 2015) using parseRM (https://github.com/ 4ureliek/Parsing-RepeatMasker-Outputs)), this sentence was removed.
We added updated and revised information to the revised manuscript.

Other concerns:
The paragraphs titled Relative evolution calculation and Gene family expansion and contraction in the methods section are poorly written, it is hard to understand what they are doing.
R: Sorry for the poor descriptions. However, in order to limiting genomic analysis and make the manuscript meet the requirement of “Date note” in Gigascience, we removed unnecessary genomic analysis, including analysis on gene family expansion and contraction. So this sentence was removed.
Lines 183-184. "to identify gene families significantly expanded or contracted"
R: Thanks. However, in order to limiting genomic analysis and make the manuscript meet the requirement of “Date note” in Gigascience, we removed unnecessary genomic analysis, including analysis on gene family expansion and contraction. So this sentence was removed.
In the paragraph titled Positive Selection Analysis in the methods section, please specify what specific evolutionary models have been used.
R: Sorry for the vagueness. We re-wrote these contents and clearly described the models used.

Powered by Editorial Manager® and ProduXion Manager® from Aries Systems Corporation

	Why are different compilations of RAxML (AVX or PHREADS) used in different parts of the job?<br>R: In our manuscript, two different compilations were used because they were run on different clusters, which were "raxmlHPC-PTHREADS-AVX" and "raxmlHPC-PTHREADS". These are in fact of no difference in performance for both are PTHREADS version, and the AVX complication means the processors support AVX vector instructions which is faster in operation. To satisfy the needs of different part of the work, we changed our processors and the corresponding raxml complications.<br>Were the 14 scaffolds obtained in the assembly expected?<br>R: Yes, they were. The female black widow spiders are reported to have 28 chromosomes.<br>Is there information on karyotypes of this species?<br>R: Although there is no karyotype of this species, there is evidence that female black widow spiders have 28 chromosomes. Thanks for your reminding and we added the reference (Zhao YN, Ayoub A, Hayashi CY. Chromosome mapping of dragline silk genes in the genomes of widow spiders (Araneae, Theridiidae). PLoS One 2010; 5(9): e12804.), and the related statement to the revised manuscript.<br>What software has been used to make the graphs of the syntenic relationships between assemblies? That information is not in methods.<br>R: Sorry for this problem, softwares Last (v1066) and Circos (v0.69-6) were used. To perform the whole genome synteny analysis, we implemented the last software (v1066) to perform the whole genome alignment using L. elegans assembly as database, in which the "lastal" command was first used to obtain MAF format alignment files and the "maf-swap" command was then used to sort the alignment and select the best one-to-one blocks. After that, Circos (v0.69-6) was used to plot the syntenic relationship graph. Thank you for your reminding, we have added it in the method part of the manuscript.<br>Line 277. CDS has not been defined previously. Where does the acronym 4dTV come from?<br>R: Sorry for the miss, we have added the definition of CDS in the manuscript. A nucleotide site is classified as nondegenerate, twofold degenerate, or fourfold degenerate (4dTV), depending on how often nucleotide substitutions will result in amino acid replacement, we have cited the literature in the manuscript.<br>Line 279. "High boostrap values" ... not results, results are not high.<br>R: Sorry for this poor description. We have corrected it in the manuscript.<br>To what extent is the phylogeny obtained in this work different from those already known? What is the contribution of this part of the work to the knowledge of the diversification of these species?<br>R: The phylogeny obtained in this work is consistent to previously reported pattern. We added information in the species in our study to spider tree of life, especially estimating the divergent time between it and the relative Theridiidae spider, P. tepidariorum.<br>In the paragraph titled "Gene family expansion and contraction of L. elegans" of results and discussion (see comment on lines 183-184 for a more correct title), the p-value <0.05, is a value corrected by the multiple testing the many families, or does it mean that the p value of each family is <0.05? Consider this problem if not. In fact, much information is lacking on how the turnover rate analysis has been done in this study. Please, expand the method section where this aspect is discussed.<br>R: Thanks for your comments. In order to limiting genomic analysis and make the manuscript meet the requirement of "Date note" in Gigascience, we removed unnecessary genomic analysis, including analysis on gene family expansion and contraction, and highlighted analyses on toxin and spidroin genes.<br>Lines 304-307. Please explain the reason for this statement or provide references.<br>R: Thanks for your comments. In order to limiting genomic analysis and make the manuscript meet the requirement of "Date note" in Gigascience, we removed unnecessary genomic analysis, including analysis on gene family expansion and contraction. So this content was removed.<br>Line 311. A. geniculata is not an ancient spider! Perhaps the authors mean that it belongs to an ancient lineage.<br>R: We are very sorry for this mistake. Yes, we could not say A. geniculata is an ancient spider. This species belongs to Theraphosidae of the ancient Mygalomorphae, a relatively ancient lineage of Araneae. However, in order to limiting genomic analysis and make the manuscript meet the requirement of "Date note" in Gigascience, we removed unnecessary genomic analysis, including general analysis on homologs. So this sentence was removed.

Lines 310-311. "This means that most of the genes have orthologous genes ..." How does this phrase match the fact that the phylogeny has only been done with 156 single-copy genes? Are all the other families with other orthology relationships?
R: In the area of evolutionary genomics, homology genes are clustered into two parts, including ortholog and paralog. Especially, the former one, ortholog genes, are clustered mainly into single copy and multi copy parts. In the analysis of OrthoMCL, a classical gene family clustering software, five types are plotted, including single copy orthologs, multiple copy orthologs, other orthologs, unique paralogs, and unclustered genes. Considering the genome quality of A. geniculate, it is not figured into this description. That means most of the genes have orthologous genes, but only 156 genes are clustered into single-copy orthologs.
However, in order to limiting genomic analysis and make the manuscript meet the requirement of “Date note” in Gigascience, we removed unnecessary genomic analysis, including general analysis on homologs. So this sentence was removed.
Line 320-321. Could it be the result of differences in the quality of annotations and assemblies rather than unique characters of this species?
R: Obviously, we could not exclude the possibilities. These could be caused by a lot of reasons, like sequencing error, assembly error, and even the computational errors of the servers or the clusters. However, based on the big data analysis, and the rapid development of sequencing and software technology, especially, our fully comparative genomics and evolutionary genomics analysis, we have sufficient confidence in the quality of our assembly and annotation.
However, in order to limiting genomic analysis and make the manuscript meet the requirement of “Date note” in Gigascience, we removed unnecessary genomic analysis, including general analysis on homologs. So this sentence was removed.
Line 327. ".this original group of arachnida ..", what does original mean? please review.
R: Sorry for the mistake. We revised the statements to: “Interestingly, A. geniculata had the slowest evolution rate. It is the only species from the relatively ancient Mygalomorphae. The result suggests that relatively ancient spider group may have relatively less selection pressure in their habitats”.
Line 332. "...eight positively selected genes and 384 genes with positively selected sites ..". In both cases genes are positively selected. Please better explain the difference and specify which models have been used.
R: Sorry for the vagueness description. We re-wrote the method and clearly indicate how to distinguish positively selected genes (PSGs) and genes with positively selected sites. We added the information on model use in PAML therein.
line 343. "..we identified four major spider venom family proteins ..". This sentence is badly constructed. Perhaps the authors meant ... "We identified proteins / copies / members of (the) four major venom gene / protein families" ...
R: Sorry for the poor writing. We revised to: “We identified members of the four major venom gene families”.
line 348. Please, indicate the p-value of these analyzes.
R: Thanks. However, considering to another review’s comments, we need to limited unnecessary genomic analysis. We hence removed analysis on gene family expansion and contraction. Accordingly, we removed this sentence.

line 360. "We analyzed the nucleotide substitutions (Ks) .... and found that L. elegans laxotroxin genes had higher (Ka / Ks)". I don't understand the first (Ks).
R: We are sorry for this misleading typo. It is the Ka/Ks rather than Ks. We revised the sentence to: “We analyzed the ratio of nonsynonymous to synonymous nucleotide substitution rate (Ka /Ks ratio) of each laxotroxin gene pair and found that L. elegans laxotroxin genes had generally higher Ka/Ks ratios compared to those in other species,…” .
line 371. "relatively more ..." is not a particularly scientific expression.
R: We are sorry for this unprofessional expression. We removed “relatively”.
Conclusions: I believe that many of the conclusions of this ms are not justified or are not supported by the results. They are more the result of speculation and interpretation. They should focus more on the description of the genome and its characteristics. The last sentences should move to the introduction.
R: Sorry for our poor writing. We updated the conclusion:
“Using the approach combining data of Illumina short reads, Nanopore long reads and Hi-C, we assembled and annotated the first chromosome-level 1.57 Gb large genome of a black widow spider, L. elegans. In this study, we confirmed phylogenetic position of this species in the spider tree of life. In addition, by analysis on hox gene family, we

Powered by Editorial Manager® and ProduXion Manager® from Aries Systems Corporation

again verified it high quality. Specifically, we focused on toxin and spidroin genes, which contribute to the distinctive features of black widow and cob web-weaving spiders and provided substantial information in terms of their composition and numbers and preliminarily demonstrate the evolution pattern of one important toxin gene family, latrotoxins. The important venom toxins contribute greatly to black widow spider's toxicity and they showed fast evolution. Generally, the genome resource data will help for deep exploration of spider genome evolution, specially on diversification of veom and web-forming. The sequence data are also first-hand templates for further application of the spider biomass."

Reviewer #3: Chromosome-level genome assembly of the black widow spider Latrodectus elegans illuminates composition and evolution of venom and silk proteins

This manuscript described the sequencing and assembly of a black widow spider, and a number of analyses comparing the obtained reference genome sequence to other spider genomes.
I realize that the manuscript is submitted as 'Data note', but a number of genome analyses are performed, and it is not very clear to me what the overall questions are that the authors want to address.
R：Thanks for your reminding. The species used in our study is a kind of black widow spider, with well-known of its venom. It is also featured with its distinctive web, which is three-dimensional and called cob-web. We hence provided more information on the venom toxins and spidroins of this species, in order to highlight the importance of the genome resource. We noticed that a recent data note published note on Gigascience showed similar kind of information (such as: Sheffer MM, Hoppe A, Krehenwinkel H, Uhl G, Kuss AW, Jensen L, Jensen C, Gillespie RG, Hoff KJ and Prost S. Chromosome-level reference genome of the European wasp spider Argiope bruennichi: a resource for studies on range expansion and evolutionary adaptation. Gigascience 2021;10: 1-12.). According to your comment, we limited unnecessary genomic analysis. For instances, we removed analysis on gene family expansion and contraction, as well as general analysis on homologs. We also made modifications of our manuscript and made it meet the format requirement of "Date Note".
The conclusion in the abstract is not a conclusion; just says 'this data is valuable', but not how/why.
R: We modified the whole content of the abstract. Specifically, the revised conclusion therein was: "We present the first chromosome-level genome assembly of a black widow spider and provided substantial toxin and spidroins gene resources. These high-qualified genomic data add valuable resources from a distinctive spider group and contribute to deep exploration on spider genome evolution, especially in terms of the important issues on diversification of venom and web-weaving pattern. The sequence data are also first-hand templates for further application of the spider biomass."
Line 47: what does it mean 'due to their adaptability'? It sounds like they special with regard to adaptation. Is this so?
R: Sorry for the misleading statement. Mechanism underlying wide distribution of spiders is a big issue and could not simply be attributed to adaptability and diverse behavior. We cautiously removed this statement.
Line 73: the authors say 'full length spidroin sequences have been found' but no references. Here are some options:
-https://www.nature.com/articles/ncomms4765
-https://www.nature.com/articles/ng.3852
-https://academic.oup.com/gigascience/article/10/1/giaa148/6067174?login=true
R: We had cited two references which were shown in the end of the sentence. Thanks for providing the references. Since "https://www.nature.com/articles/ncomms4765": the spidroin sequences in this paper is not full-length, we added the other two papers to our revised manuscript.
Line 309: The A. geniculata genome is of much lower quality than the other genomes. Therefore, the conclusions given in this section should be removed. This should also apply to line 326.
R: Thanks for your reminding. In order to limiting genomic analysis and make the manuscript meet the requirement of "Date note" in Gigascience, we removed unnecessary genomic analysis, including general analysis on homologs. So this content was removed.
I think that you should cite the papers publishing all the spider genome sequences (plus I. scapularis and C. sculpturatus) that you download and analyze.

	Generally, I think that the manuscript uses too few citations. Another example is line 256 where the authors say that the results of their hox gene analysis are similar to others species, but without any references. Actually, I do not think there is a single reference in the result/discussion section, but may have missed it/them!!!<br>R: Thanks for your reminding. We made intensive modifications in the manuscript and added quite a few new references to support our results and discussion. Line 256, we added the references: Schwager EE, Sharma PP, Clarke T, et al. The house spider genome reveals an ancient whole-genome duplication during arachnid evolution. BMC Biol. 2017; 15 (1):62. Published 2017 Jul 31. doi: 10.1186/s12915-017-0399-x; Sheffer MM, Hoppe A, Krehenwinkel H, et al. Chromosome-level reference genome of the European wasp spider Argiope bruennichi: a resource for studies on range expansion and evolutionary adaptation. Gigascience. 2021; 10 (1): giaa148. doi:10.1093/gigascience/giaa148; A chromosome-level genome of the spider Trichonephila antipodiana reveals the genetic basis of its polyphagy and evidence of an ancient whole-genome duplication event.<br>The figures are very busy with a lot of tiny text, and I have a hard time seeing that they read when fitted into a paper.<br>R: Sorry. We have enlarged the text to make the figures more readable.
Additional Information:	
Question	Response
Are you submitting this manuscript to a special series or article collection?	No
**Experimental design and statistics**<br><br>Full details of the experimental design and statistical methods used should be given in the Methods section, as detailed in our Minimum Standards Reporting Checklist. Information essential to interpreting the data presented should be made available in the figure legends.<br><br>Have you included all the information requested in your manuscript?	Yes
**Resources**<br><br>A description of all resources used, including antibodies, cell lines, animals and software tools, with enough information to allow them to be uniquely identified, should be included in the Methods section. Authors are strongly encouraged to cite Research Resource Identifiers (RRIDs) for antibodies, model organisms and tools, where possible.<br><br>Have you included the information requested as detailed in our Minimum	Yes

Standards Reporting Checklist?	
**Availability of data and materials**<br><br>All datasets and code on which the conclusions of the paper rely must be either included in your submission or deposited in publicly available repositories (where available and ethically appropriate), referencing such data using a unique identifier in the references and in the "Availability of Data and Materials" section of your manuscript.<br><br>Have you have met the above requirement as detailed in our Minimum Standards Reporting Checklist?	Yes

Manuscript

Click here to access/download;Manuscript;spiderGonomeMSGiga20220422

Chromosome□level genome assembly of the black widow spider *Latrodectus elegans* illuminates composition and evolution of venom and silk proteins

Zhongkai Wang[1, 2, †], Kesen Zhu[1, †], Haorong Li[2, †], Lei Gao[1], Huanying Huang[1], Yandong Ren[2, *], and Hui Xiang[1, *]

1. Guangdong Provincial Key Laboratory of Insect Developmental Biology and Applied Technology, Institute of Insect Science and Technology, School of Life Sciences, South China Normal University, Guangzhou, 510631, PR China.

2. School of Ecology and Environment, Northwestern Polytechnical University, Xian, 710072, PR China.

Zhongkai Wang [0000-0003-0578-5735];

Kesen Zhu [0000-0002-8232-592X];

Haorong Li [0000-0003-4289-2847];

Lei Gao [0000-0001-5708-029X];

Huanying Huang [0000-0002-7286-2772];

Yandong Ren [0000-0003-1595-8994];

Hui Xiang [0000-0002-8695-9006]

† These authors contributed equally to this work.

[*] These authors jointly directed this work. Correspondence should be addressed to H. X. (xiang_shine@foxmail.com), Y. R. (renyandong90@126.com).

Abstract

Background: Black widow spider has both extraordinarily neurotoxic venom and three-dimensional cob-webs composing of diverse types of silk. However, high-quality reference genome for black widow spider was still unavailable, which hindered deep understanding and application of the valuable biomass.

Findings: We assembled the *L. elegans* genome and the genome size is 1.57 Gb with contig N50 of 4.34Mb and scaffold N50 of 114.31 Mb. Hi-C scaffolding assigned 98.08% of the genome to 14 pseudo-chromosomes, and with BUSCO (Benchmarking Universal Single-Copy Orthologs) completeness analysis revealed that 98.4% of the core eukaryotic genes were completely present in this genome. Annotation of this genome identified that repetitive sequences account for 506.09 Mb (32.30%) and 20,167 protein-coding genes and specifically, we identified 55 toxin genes and 26 spidroins and provide preliminary analysis their composition and evolution.

Conclusions: We present the first chromosome-level genome assembly of a black widow spider and provided substantial toxin and spidroins gene resources. These high-qualified genomic data add valuable resources from a representative spider group and contribute to deep exploration on spider genome evolution, especially in terms of the important issues on diversification of venom and web-weaving pattern. The sequence data are also first-hand templates for further application of the spider biomass.

Keywords: Chromosome-level genome, *Latrodectus elegans*, evolutionary rate, venom, spidroin

Data Description

Background

Spiders is a highly diverse and abundant group of predatory arthropods and more than 49,000 spider species have been described to date [1, 2]. They are found in a wide range of habitats such as underground caves, tropical rainforests, deserts, and on glaciers [3-5]. Spiders are of special interest due to their distinctive characteristics such as spider silk and venom. Spider silk has unique mechanical properties and can potentially be used by the military industry, in medicine and in other fields [6-8]. Spider venom has a complex composition and is rich in many biologically active substances. This makes it valuable for possible applications in pharmacological tools, reagents, drug precursors, biological pesticides and other biologically active substances [9, 10]. In-depth studies of the biochemical and physical properties of spider silk and venom may require the identification of the complete sequence of spider genes, but the lack of high-quality genomic data hinders these studies.

Latrodectus spp. are known as black widow spiders. They are featured and of great interest for their extraordinarily neurotoxic venom [11, 12]. Spider venom is a complex mixture of toxins with different biological activities, from small molecular weight compounds to protein and peptide substances. More than 100 different chemical components have been identified in spider venom [13, 14]. Compared with most other venomous animals, black widow spiders not only contain toxins in the venom glands but also in their entire body, including their legs and abdomen. Toxins are also found in spider eggs and newborn offspring. This unique feature makes black widow spider venom components more diverse [15-17]. However, studies on black widow toxins are relatively fragmented, and information on the components of black widow

venom still remains limited. Systematic identification and analysis of all the spider toxin genes with full length sequences is now a top priority [18]. In addition, the black widow spider is distinctive from those spiders that construct classic two-dimensional aerial capture webs. The spider webs of black widow spiders are three-dimensional and are called cob-webs [19-21]. Therefore, genetic deciphering of black widow spider silk provides data and clues for the diversification of spiders. The full lengths of some major silk proteins (spidroins) have been identified, but the systematic analysis on spidrions of cob-weaving spider is still lacking [22-25].

High-quality chromosome-level genomes of *Latrodectus* spp. will provide important resources for deciphering the spider toxin and spidroin genes. Only two spider species (*Trichonephila antipodiana* and *Argiope bruennichi*) have previously been sequenced based on long sequencing reads and assembled to the chromosome level [2, 25]. High-quality chromosome-level spider genome resources remain scarce. In this study, we combined Oxford Nanopore technologies and high-throughput chromosome conformation capture sequencing [26, 27] to generate a high-quality chromosome-level reference genome for *Latrodectus elegans* (NCBI: txid2857379) and systematically analyzed venom proteins and spidrons. These data are a reference for future studies on the range of spider gene diversity.

Methods

Biological material, Genome DNA extraction and Sequencing

Two female adult *L. elegans* spiders were obtained from Binzhou county, Dali City, Yunnan province, China in 2021. The live samples were sent to Beijing Biomarker Technologies.

Co. LTD for sample handling, DNA and RNA extraction and sequencing. Briefly, the spiders were cleansed and grinded in liquid nitrogen, respectively. One spider was used for Hi-C seq and RNA-seq. The other was for genome sequencing including next-generation and Nanopore sequencing. Genomic DNA was extracted using a Blood and Cell Culture DNA Mini Kit (Qiagen) according to the protocol. The short paired-end inserts libraries, including Hi-C and genome sequencing, were constructed using the Illumina platform protocol, and 150-bp paired-end reads were generated using the Illumina NovaSeq platform (Illumina NovaSeq 6000 Sequencing System, RRID:SCR_020150). A genome long read library was constructed and sequenced on the Nanopore oxford platform (Oxford Nanopore Technologies, RRID: SCR_003756). Total RNAs of the whole body were extracted using TRIzol (Invitrogen) according to manufacturer instructions and RNA-seq was generated on the Illumina NovaSeq platform.

Quality control and genome characteristics evaluation

For the Nanopore long reads, reads with mean quality > 7 were retained using an in-house Perl script for further assembly. For the Illumina short reads, the duplicated reads and the adaptors were removed. Reads with more than 10% unknown bases or read pairs with more than 30% low-quality bases were also excluded. Each read was removed 5 bp at both head and tail.

To investigate the genome characteristics of *L. elegans*, all the filtered short-insert reads were used for k-mer analysis. The genome size was estimated by using the formula: $G = K_{number}/K_{depth}$, where K_{number} and K_{depth} represent the total number and peak depth of 21-mer, respectively. The genome size was calculated by GenomeScope (GenomeScope, RRID:SCR_017014) v2.0 [28], with the k value set as 21 and other parameters set as default.

Genome assembly and evaluation

To obtain a high-quality genome, all of the filtered Nanopore long reads were assembled into contigs using Nextdenovo software (v2.4) [29] with core parameters: -d 40 -g 1.74 g. The single-base errors in the genome assembly were corrected using all the filtered Illumina short reads by NextPolish (v1.3.1) with parameters: rerun = 3, -max_depth = 100. The Hi-C sequencing reads were mapped to the polished contig assembly to anchor the contigs into chromosomes using the 3D *de novo* assembly software (v170123) [30].

To evaluate the quality and accuracy of the assembled genome, the following three strategies were used. First, the quality of the assembled genome and gene completeness were assessed using BUSCO software (BUSCO, RRID:SCR_015008) v5.2.2 [31] with the core gene set of the eukaryote and metazoan databases, respectively. Second, all the filtered short reads sequenced using the Illumina platform were mapped to the assembled genome by BWA software (BWA, RRID:SCR_010910) v0.7.12-r1039 [32] to evaluate the genome integrity. Third, the transcripts of *L. elegans* were assembled using Bridger (Bridger, RRID: SCR_017039) (version: r2014-12-01) [33] and then mapped to the assembled genome using BLAT software. To perform the synteny analysis, we implemented the Last software (LAST, RRID:SCR_006119) (v1066) [34] to achieve the whole genome alignment using *L. elegans* assembly as database, in which the "lastal" command was first used to obtain MAF format alignment files and the "maf-swap" command was then used to sort the alignment and select the best one-to-one blocks. After that, Circos (Circos, RRID:SCR_011798) v0.69-6 [35] was used to plot the syntenic relationship graph.

Repetitive sequence annotation

Tandem repeats and transposable elements (TEs) in the assembled genome were both annotated. Tandem Repeat Finder software (v4.09) [36] was used for tandem repeats prediction. The TEs were identified on both protein and DNA levels. On the protein level, the RepeatProteinMask (RM-BLASTX) [37] was used to search TEs using the known protein database. On the DNA level, both *de novo* libraries and Repbase libraries in RepeatMasker (RepeatMasker, RRID: SCR_012954) (open-4.0.7) were used. *De novo* libraries were built by RepeatModeler (RepeatModeler, RRID:SCR_015027) [38], and the consensus sequences were used as RepeatMasker input files. The insertion time of each TE sequence was estimated using a Kimura distance based analysis [39] using parseRM [40].

Protein-coding gene annotation

To obtain proper gene annotation results, all the TEs were masked before gene annotation. *De novo* annotation, homology-based annotation, and RNAseq-based annotation were used in this study. First, Augustus software (Augustus, RRID:SCR_008417) v2.5.5 [41] was used for *de novo* annotation using default parameters. Second, the protein sequences of the gene sets of *Acanthoscurria geniculata* (GCA_000661875.1) [42], *Araneus ventricosus* (GCA_013235015.1) [43], *Argiope bruennichi* (GCA_015342795.1) [25], *Parasteatoda tepidariorum* (GCF_000365465.2) [44], *Stegodyphus dumicola* (GCF_010614865.1) [45], *Stegodyphus mimosarum* (GCA_000611955.2) [42], *Trichonephila antipodiana* (GigaDB) [2] and *Trichonephila clavipes* (GCA_002102615.1) [46] were downloaded from NCBI or GigaDB and used for homology-based predictions, one species at a time. We chose the longest isoform of each gene to obtain the non-redundant protein sequences of each gene set. The protein sequences were used as query to search for orthologous regions in the *L. elegans*

genome by tblastn (TBLASTN, RRID: SCR_011822) with an e-value of 1e-5. The results were subjected to GeneWise software (GeneWise, RRID:SCR_015054) v2.4.1 [47] to predict gene structure. Third, all the filtered RNA-seq reads were assembled into transcripts using Bridger (version: r2014-12-01) [33] and then aligned to the assembled genome using BLAT (BLAT, RRID:SCR_011919) (v34, identity > 90%, coverage > 90%) [48], and the PASA (PASA, RRID:SCR_014656) [49] was then used to link the spliced alignment. For the results generated from the three methods, EvidenceModeler (EVidenceModeler, RRID: SCR_014659) version 1.1.1 [50] was used to integrate them into the final protein-coding gene set.

These genes were functionally annotated by homology-based searches against InterProScan/GO, KEGG, Swissprot, TrEMBL and Cog. InterProScan (InterProScan, RRID:SCR_005829) v4.8 [51] was used to screen proteins against five databases (Pfam, release 27.0, prints, release 42.0, prosite, release 20.97, ProDom, 2006.1, and smart, release 6.2). In addition, the KEGG[52], SwissProt, TrEMBL and Cog were used for annotation by BLAST software (NCBI BLAST, RRID:SCR_004870) v2.3.0 [53].

Orthologous gene identification

The annotated gene sequences of *L. elegans* along with other nine different species, including *Acanthoscurria geniculata* (GCA_000661875.1), *Argiope bruennichi* (GCA_015342795.1), *Parasteatoda tepidariorum* (GCF_000365465.2), *Trichonephila antipodiana* (GigaDB), *Trichonephila clavipes* (GCA_002102615.1), *Centruroides sculpturatus* (GCF_000671375.1), *Stegodyphus dumicola* (GCF_010614865.1), *Stegodyphus mimosarum* (GCA_000611955.2) and *Ixodes scapularis* (GCF_016920785.1), were used to identify the orthologous genes using OrthoMCL software (OrthoMCL, RRID:SCR_007839) v2.0.9 [54] and default parameters.

Phylogenetic analysis and divergence time estimation

The phylogenetic relationships and divergence time between the 10 test species (*L. elegans, A. geniculata*, *A. bruennichi*, *P. tepidariorum*, *T. antipodiana*, *T. clavipes*, *C. sculpturatus*, *S. dumicola*, *S. mimosarum* and *I. scapularis*) were analyzed using the previously identified single-copy genes. All the well-aligned single-copy orthologous genes in each species were concatenated to one super gene for each species. Maximum likelihood-based phylogenetic analysis was conducted using RAxML (RAxML, RRID:SCR_006086) v8.2.10 [55], and the parameters were set as follows: raxmlHPC-PTHREADS -m PROTGAMMAAUTO -N 100 -p 12345 -o *I. scapularus* -# 100. Then, the MCMCtree program in the PAML package (PAML, RRID:SCR_014932) v4.8 [56] was used for divergence time calculation. All of the fossil records were downloaded from the TIMETREE database (TimeTree, RRID: SCR_021162) [57] for result calibration.

Analysis on relative evolution rate

The well aligned concatenated protein sequences of single-copy orthologs of the ten species were used to generate relative evolution rate. Two methods were used, i.e., Tajima's relative rate test and two cluster analyses. Tajima's relative rate test was generated by MEGA software (MEGA Software, RRID:SCR_000667) v10 [58]. We specified *I. scapularus* as the outgroup species and tested the relative evolution rate between *L. elegans* and other species. The Chi-square test was used to test which species has faster evolution rate compared to the other one. Two cluster analysis was generated by LINTRE software via the tpcv model [59]. We also specified *I. scapularus* as the outgroup species and tested the relative evolution rate between *L. elegans* and other species.

Positive selection analysis

Single-copy orthologs from the five relatively closely related species of orb-web weaving spiders, i.e., *L. elegans*, *P. tepidariorum*, *A. bruennichi*, *T. clavipes* and *T. antipodiana*. As well as their species tree were used to identify the potential positively selected genes (PSGs) and the genes with positively selected sites in *L. elegans*, using branch model and branch site model in the Codeml tool of the PAML package (v4.8) [56] respectively. Briefly, the rate ratio (ω) of nonsynonymous to synonymous nucleotide substitutions was estimated. One ratio model was used to detect average ω across the species tree ($\omega0$). For each gene, two ratio branch model was used to detect the ω of the appointed branch (*L. elegans*) to test the ($\omega1$) and ω of all other branches ($\omega\_background$). A likelihood ratio test was performed to compare the fit of the two ratio models with the one ratio model to determine whether the gene is positively selected in the appointed branch ($\omega1>\omega0$; $\omega1>\omega\_background$; $\omega1>1$; P-value<0.05). If the gene is not positively selected, then the branch site model was used to detect amino acid sites likely to be is positively selected in the appointed branch using Bayes Empirical Bayes (BEB) analysis [56].

Toxin gene analysis

To locate the toxin genes in the genome of both *L. elegans* and all other related species, we investigated and downloaded toxin sequences from the ArachnoServer database (v3.0) [60] including four families, namely the CRISP family, the ICK family, the latrodectin family and the latrotoxin family. For CRISP family and latrodectin family, blastp (BLASTP, RRID: SCR_001010) was applied to search the candidate toxin genes against protein sequences of all associated species using '-outfmt 7 -evalue 1e-5' as the key parameters. Clustalw2 was used to perform the multiple sequence alignment to align all the latrotoxin gene sets. Because ICK

family is a group of short peptides with ICK motif, we identified the ICK toxins referring Haney et al.'s approach [61]. Firstly, we retrieved the peptides less than 200 amino acid and have at least six cysteines constituting the NCNCNCCNCNC (N refers to any other amino acid) motif. Secondly, we used the online tool ClanTox [62] to evaluate the toxin potential of the above candidate ICK toxins. Thirdly, we used SignalP (SignalP, RRID: SCR_015644) v5.0 to predict signal peptide of them and those without signal peptide was removed out. After further manual check, we finally identified the ICK toxins. As to latrodectin family genes, since there are rather few genes in ArachnoServer v3.0 database, we then retrieved the known sequences of latrodectin from NCBI. We applied the Blastp with e-value cutoff of 10-5 to search the candidate homolog proteins. Then the homolog was further verified by hmmer (Hmmer, RRID:SCR_005305) v3.3 [63]. In brief, all above-found sequences were merged together and applied Clustalw2 to perform the multiple sequence alignment which were then transformed into stockholm format using online toolset [64]. The aligned sequences were used to construct the HMM profile using hmmbuild, a binary software of hmmer v3.3. Finally, hmmsearch was applied for the peptide of each species, to find the potential domains of all species using key parameters of "--domtblout" and e-value of 10-5.

The toxin gene distribution of *L. elegans* was plotted by MG2C software [65] We then constructed the phylogeny of the latrotoxin genes set using maximum likelihood methods in the RAxML software (v8.2.10) [55], in which raxmlHPC-PTHREADS-AVX was used as the main model with 100 bootstrap replicates, the Latrotoxin gene of *I. scapularis* was specified as

the outgroup, and the key parameters were set as '-n orthology -m PROTGAMMAAUTO -f a -x 12345 -N 100 -p 12345 -o *I. scapularis*_Latrotoxins_XP_040064028.1.' Finally, the phylogenetic tree of the latrotoxin genes was visualized in iTOL software (iTOL, RRID: SCR_018174) [66, 67]. ParaAT [68] was applied to achieve multiple protein-coding DNA alignments using '-m muscle -p proc -f axt' as key parameters. Then we calculated the ratio of nonsynonymous substitutions over synonymous substitutions (Ka/Ks) for each pair of genes separately using KaKs Calculator software (v2.0) [69]. All Ka/Ks values were plotted and grouped by species.

Analysis of spidroins

To annotate the various types of spidroins (MaSp, MiSp, TuSp, AcSp, Flag, AgSp and PySp) of *L. elegans*, we initially downloaded the amino acid sequences of spidroins of the *L. hesperus*, *T. clavipes, A. bruennichi* and *A. ventricosus* from the NCBI database (ABR68855.1, ABR68856.1, ARA91152.1, ARA91182.1, AWK58725.1, AFP57565.1, AFX83557.1, AAY28931.1, ACV41934.1, PRD24320.1, PRD20448.1, PRD23654.1, PRD24510.1, PRD30268.1, PRD24772.1, GFY36469.1, PRD26655.1, PRD23989.1, GFY34959.1, PRD26201.1, PRD35275.1, GFY35027.1, ADK92884.1, AFN54363.1, AGB35874.1, GBM54680.1, GBN00528.1, GBN00527.1, GBN25680.1, AFV31615.1, GBM96188.1, GBN20389.1, GBN20387.1, GBL96802.1, GBL96803.1, GBN70256.1, AUH99620.1, QKE59598.1, QKE59599.1, QBA85221.1, GBN88500.1). Using the seven different types of spider silk genes collected above as queries, we searched the annotated proteins of *L. elegans* by blastp with the cut-off of e-value less than 10^{-5}. The retrieved sequences of *L. elegans* were then manually filtered to remove missense mutation sequences according to the conserved

domains of the spidroins. These spidroin sequences of *L. elegans* were further verified by blastp against the NCBI non-redundant protein database [70]. The chromosome distribution of the spidroin genes were then plotted by MG2C software (v2.1). Sequences were aligned using MEGA software (v10) and further reorganized as categories for further research: complete, internal gap, N-terminal, C-terminal, and repetitive sequence. To identify the spidroins in the other two spider species, i.e., *P. tepidariorum* and *T. antipodiana*, we searched the annotated non-redundant protein sequences of the related species by blastp with the cutoff of e-value as 1e-5, identity as 0.3, and alignment ratio as 0.5.

Results and Discussion

Genome assembly

Firstly, we generated 66.49 Gb Illumina short-insert-size reads with almost 38.20X depth (**Table S1**). The 21-mer analysis showed that the genome size of *L. elegans* is ~1.74 Gb (**Figure S1**). Then, 106.80 G filtered Nanopore reads (N50 is 24.01 Kb, ~61.37-fold of the genome) were obtained (**Table S2**). The Nanopore filtered reads were assembled into contigs and further assembled into chromosomes using Hi-C reads (77.58 Gb, ~44.57-fold of the genome) (**Table S3**). We obtained a 1.57 Gb genome assembly with contig N50 of 4.34Mb and scaffold N50 of 114.31 Mb (**Table S4**). A total of 14 chromosomes were assembled with lengths ranging from 70.40 Mb to 133.92 Mb (**Figure 1A**, **Table S5**). The number of chromosomes is consistent to previously report to female window spiders [71]. To validate the completeness and accuracy of the *L. elegans*' genome, assembled transcripts mapping ratio, short reads mapping ratio and Benchmarking Universal Single-Copy Orthologs (BUSCO, v5.2.2) were used in the analysis.

All the assembled transcripts were aligned to the genome, and 77,191 of 85,772 (90.00%) transcripts can be found in the assembled genome (**Tables S6–S8**). We aligned all the filtered short reads to the assembled genome, and more than 357.88 million reads (99.28%) could be mapped to the genome (**Table S9**). We also found that 251 of 255 (98.4%) and 930 of 954 (97.5%) core eukaryote and metazoan genes were successfully identified in the genome, respectively (**Table S10**), and this assembly quality was comparable with that of the close-related species (**Table S11**). We also identified the Hox genes in all 10 species (*A. geniculata, S. mimosarum, T. clavipes, A. bruennichi, P. tepidariorum, C. sculpturatus, S. dumicola, I. scapularus*, *T. antipodiana* and *L. elegans*). These results showed that *L. elegans* has two Hox gene clusters. Both of these two clusters are complete and continuous, which is comparable to other related species (**Figure S2**) [2, 25, 44]. These results indicate that the integrity and accuracy of the assembled genome are good.

Genome annotation

Both tandem repeats and TEs were annotated in the assembled genome, and a total of ~506.09 Mb repeat sequences were identified that accounted for 32.30% of the assembled genome (**Table S12**). For TEs, there were 9.69% of DNA (151.78 Mb), 4.50% of LINE (70.53 Mb), 2.48% of LTR (38.89 Mb) and 1.09% of SINE (17.15 Mb) in this genome (**Table S13**). For protein-coding genes, 20,167 genes were annotated with 81.03% of the genes having homologous hits in the public databases (**Table S14**). These genes showed high similarity with related species in gene length distribution, CDS (coding sequence) length distribution, exon length distribution and exon number distribution (**Figure S3**). The basic genome statistics of this genome, including gene density, tandem repeat, LTR, LINE, SINE, DNA TEs and GC

content, are shown in **Figure 1B**. We checked the synteny block between *L. elegans* and other species of Arachnida (*S. dumicola, P. tepidariorum* and *T. clavipes*, **Figure 1C–1E**). The results showed that the assembled genome has a good genome synteny relationship with these species.

Phylogenetic relationship of *L. elegans* and other related species

To compare the genomics of *L. elegans* with other spider species, we identified the orthologous/paralogous genes among these species. A total of 28,587 gene families were clustered in these 8 spiders and two outgroup species, and 156 single-copy genes were identified. The phylogenetic relationship of these 10 species was determined using the amino acid and nucleotide acid sequences of CDS, and the fourfold degenerate synonymous site (4dTV) [72] of the single-copy genes was concatenated into a super-gene in each species. Each method showed the same phylogenetic relationship with high bootstrap values (**Figures S4–S6**). This relationship is consistent to the well documented spider tree of life [73]. *L. elegans* is closely related to *P. tepidariorum*, both of which are of Theridiids. A calculation of the estimated divergence time suggested that the two species diverged ~73.0 million years ago (Mya) (**Figure 2A**).

TE insertion history of Arachnida

We checked the types of TEs and the TE insertion time of all 10 species and found that the TE contents are significantly different. In the Theridiidae, including *L. elegans* and *P. tepidariorum*, the TE insertion times are concentrated at 20–35 million years ago. However, the TE insertion times of other species are much older than that of the Theridiidae. Besides, the insertion times of *L. elegans* (~35 million years) and *P. tepidariorum* (~20 million years) are much more recent than the divergence between two species, which suggests that the TE insertion event may have

happened after their divergence (**Figure 2B**).

Relative evolution rate of species

Species in different environments may face different selection pressures, and the relative rate of evolution can reflect this. The relative evolution rate results showed that *L. elegans* had the fastest evolution rate among these species and suggested that it has experienced strong selection pressure. *A. geniculata* had the slowest evolution rate (**Figure S7; Tables S17–S18**). It is the only species from the relatively ancient Mygalomorphae. The result suggests that relatively ancient spider group may have relatively less selection pressure in their habitats. However, due to its low genome quality, this result is pending further verification.

Positively selected genes

Using five relatively closely related species of orb-web weaving spiders belonging to the Araneoidea, i.e., *L. elegans*, *P. tepidariorum*, *A. bruennichi*, *T. clavipes* and *T. antipodiana*, we identified eight positively selected genes and 348 genes with positively selected sites in *L. elegans* (**Table S17**). In these genes, *lhx9* was the only gene related to gonadal development. The structures of this gene in these species were constructed, and *L. elegans* was the most unique of all species (**Figure S8**). These results indicated that the gonadal development of *L. elegans* may differ from that of the other species. The *CaMKI* gene belongs to calcium/calmodulin-dependent protein kinase family, and the other gene, *CaMKII*, is associated with OA signaling, which may affect Ca^{2+} signaling or adjust intracellular cAMP levels in vivo. In the spider *Cupiennius salei, CaMKII* may also be a downstream modulator of OA signaling in spider VS-3 neurons [74], which is related to cell excitability.

Venom gene analysis

The venoms of *Latrodectus* spp. are famous for their potency and ability to cause extreme and long-lasting pain. Based on genome-wide comparative analysis, we identified members of the four major venom gene families: CRISP family, ICK family, latrodectin family and latrotoxin family. The severe symptoms of *Latrodectus* envenomation are largely attributed to latrotoxins [75]. Consistently, we found that, compared to other spiders, the number of genes in the latrotoxin family of *L. elegans* and the house spider *P. tepidariorum* has undergone lineage-specific expansion. The number of latrotoxin genes found in *P. tepidariorum* and *L. elegans* was 30 and 50, respectively, which are more than that found in other spiders (1–12) (**Figure 3A**). We also identified six toxins from the CRISP family and two from the ICK family in *L. elegans* respectively. The latrotoxin and CRISP families are both ancient and relatively conserved toxins which exist in all the test species of Arachnida. Toxins from the ICK family seem unique to the Araneoidea (**Figure 3A**). Genic loci of all these venom toxins were mapped on 11 chromosome scaffolds (Chromosomes 2, 3, 5, 7, 8, 9, 10, 11, 12, 13 and scaffold 39). The majority of latrotoxin genes were located on Chromosome 11, and there was a remarkable tandem duplication of latrotoxin genes on Chromosome 11 (**Figure 3B**). Phylogenetic analysis showed that the latrotoxins experienced substantial gene duplication and diversification in the two Theridiidae spiders, including *L. elegans* and *P. tepidariorum*, and that latrotoxins of *L. elegans* in the clade of latest expansion were mostly located on Chromosome 11 (**Figure 3C**). We analyzed the ratio of nonsynonymous to synonymous nucleotide substitution rate (Ka/Ks ratio) of each latrotoxin gene pair and found that *L. elegans* latrotoxin genes had generally higher Ka/Ks ratios compared to those in other species, suggesting their rapid evolution (**Figure 3D**).

Spidroin gene analysis

A female cob-weaving spider can have up to seven morphologically differentiated types of silk glands, each of which can produce a silk protein, namely spidroin. The classes of spidroins include major ampullate spidroin (*MaSp*), minor ampullate spidroin (*MiSp*), flagelliform spidroin (*Flag*), aggregate spidroin (*AgSp*), aciniform spidroin (*AcSp*), tubuliform spidroin (*TuSp*) and pyriform spidroin (*PySp*). We identified six unique annotated genes for *MaSp*, eight for *MiSp*, two for *Flag*, five for *AgSp*, two for *PySp*, two for *AcSp* and one for *TuSp* in *L. elegans* **(Figure 4A)**. It is notable that *L. elegans* has relatively more *Misp* genes. MiSp is mainly used for inelastic temporary spirals during web building. However in cob web spiders, *Misp* contributes to prey-wrapping in cobweb weavers [76]. The more copies of *Misps* in *L. elegans* genome might evolve to strength the function of prey-wrapping [77]. All *MiSps* were clustered on Chromosome 14, suggesting that they may have diversified via tandem duplication **(Figure 4B)**. Other multi-copy spidroin genes such as *MaSp*, *AgSp*, *PySp* and *AcSp* were also distributed in clusters on Chromosome 11, 5, 6 and 12, respectively, suggesting that tandem duplication is the main type of duplication of spidroins. The two *Flag* genes, however, are located on Chromosomes 6 and 7 **(Figure 4B).**

Conclusion

Using the approach combining data of Illumina short reads, Nanopore long reads and Hi-C reads, we assembled and annotated the first chromosome-level 1.57 Gb large genome of a black widow spider, *L. elegans*. In this study, we confirmed phylogenetic position of this species in the spider tree of life. In addition, by analysis on Hox gene family, we again verified it high quality. Specifically, we focused on toxin and spidroin

genes, which contribute to the distinctive features of black widow and cob web-weaving spiders and provided substantial information in terms of their composition and numbers and preliminarily demonstrate the evolution pattern of one important toxin gene family, latrotoxins. The important venom toxins contribute greatly to black widow spider's toxicity and they showed fast evolution. Generally, the genome resource data will help for deep exploration of spider genome evolution, especially on diversification of venom and web-forming. The sequence data are also first-hand templates for further application of the spider biomass.

Data availability

All raw sequencing data and the genome assembly of *L. elegans* underlying this article are available at the NCBI and can be accessed with Bioproject ID PRJNA745004. All supporting data and materials are available in the *GigaScience* GigaDB database [78] .

Additional Files

Table S1. The statistics of sequencing reads on Illumina platform.

Table S2. The statistics of sequencing reads on Nanopore platform.

Table S3. The statistics of Hi-C sequencing reads.

Table S4. The statistics of the polished genome and chromosome-level genome.

Table S5. Statistics of the assembled chromosome-level genome via 3D *de novo* assembly software.

Table S6. The statistics of RNA sequencing reads on Illumina platform.

Table S7. The statistics of the assembled transcripts by Bridger of 5 organs/tissues.

Table S8. The statistics of the transcripts mapping ratio on the assembled genome.

Table S9. The statistics of the short reads mapping ratio on the assembled genome.

Table S10. The quality evaluation of assembled genome by BUSCO software.

Table S11. Comparison of the related genomes with our chromosome-level genome.

Table S12. The statistics of the annotated repeat sequences in our assembled genome.

Table S13. The statistics of the annotated repeat sequences in our assembled genome by *de novo* prediction.

Table S14. The functional annotation of the predicted protein-coding genes.

Table S15. Relative evolution rate among these species by LINTRE software.

Table S16. Relative evolution rate among these species by MEGA software.

Table S17. Statistics of positively selected genes of *L. elegans*.

Figure S1. 21-mer analysis of *L. elegans* genome.

Figure S2. Annotation and comparison of the Hox clusters among these 10 species.

Figure S3. Distribution of gene parameters in various species.

Figure S4. Phylogenetic relationship among the 10 species inferred by the amino acid sequences of the single-copy genes.

Figure S5. Phylogenetic relationship among the 10 species inferred by the nucleotide acid sequences of the single-copy genes.

Figure S6. Phylogenetic relationship among the 10 species inferred by the *4dTV* data of the single-copy genes.

Figure S7. Relative evolutionary rate of species.

Figure S8. Gene structure of *lhx9* in these species.

Competing interests

The authors declare that they have no competing interests.

Authors' Contributions

H.X. and Y.R. conceived and designed the investigation. K. Z., L.G. and H. H. performed field and laboratory work. Z. W. assembled the genome. H. L. performed the Hi-C scaffold. Y. R., K.S.Z, and L. G. analyzed the data. H. X., K. S. and L. G. contributed materials and reagents. Y. R. and K.S.Z. wrote the paper. H.X and Y.R. revised the manuscript. All the authors read and approved the final manuscript.

Acknowledgements

This work was supported by the National Natural Science Foundation of China (32070411 , 32000383), a grant from the Natural Science Foundation of Guangdong Province, China (2019A1515011012) and the Laboratory of Lingnan Modern Agriculture Project (NZ2021019).

References:

1. Slater GS and Birney E. Automated generation of heuristics for biological sequence comparison. Bmc Bioinformatics. 2005;6 1:1-11.
2. Fan Z, Yuan T, Liu P, Wang LY, Jin JF, Zhang F, et al. A chromosome-level genome of the spider Trichonephila antipodiana reveals the genetic basis of its polyphagy and evidence of an ancient whole-genome duplication event. Gigascience. 2021;10 3 doi:ARTN giab016 10.1093/gigascience/giab016.
3. Wirta HK, Weingartner E, Hamback PA and Roslin T. Extensive niche overlap among the dominant arthropod predators of the High Arctic. Basic Appl Ecol. 2015;16 1:86-92. doi:10.1016/j.baae.2014.11.003.
4. Moulder BC and Reichle DE. Significance of Spider Predation in Energy Dynamics of Forest-Floor Arthropod Communities. Ecol Monogr. 1972;42 4:473-98. doi:Doi 10.2307/1942168.
5. Dimitrov D, Lopardo L, Giribet G, Arnedo MA, Alvarez-Padilla F and Hormiga G. Tangled in a sparse spider web: single origin of orb weavers and their spinning work unravelled by denser taxonomic sampling. P Roy Soc B-Biol Sci. 2012;279 1732:1341-50. doi:10.1098/rspb.2011.2011.
6. Wright S and Goodacre SL. Evidence for antimicrobial activity associated with common house spider silk. BMC Res Notes. 2012;5:326-. doi:10.1186/1756-0500-5-326.
7. Altman GH, Diaz F, Jakuba C, Calabro T, Horan RL, Chen JS, et al. Silk-based biomaterials. Biomaterials. 2003;24 3:401-16. doi:Pii S0142-9612(02)00353-8 Doi 10.1016/S0142-9612(02)00353-8.
8. Kluge JA, Rabotyagova U, Leisk GG and Kaplan DL. Spider silks and their applications. Trends Biotechnol. 2008;26 5:244-51. doi:10.1016/j.tibtech.2008.02.006.
9. King GF and Hardy MC. Spider-Venom Peptides: Structure, Pharmacology, and Potential for Control of Insect Pests. Annu Rev Entomol. 2013;58:475-96. doi:10.1146/annurev-ento-120811-153650.
10. Escoubas P, Diochot S and Corzo G. Structure and pharmacology of spider venom neurotoxins. Biochimie. 2000;82 9-10:893-907. doi:Doi 10.1016/S0300-9084(00)01166-4.
11. Garb JE, Gonzalez A and Gillespie RG. The black widow spider genus Latrodectus (Araneae : Theridiidae): phylogeny, biogeography, and invasion history. Mol Phylogenet Evol. 2004;31 3:1127-42. doi:10.1016/j.ympev.2003.10.012.
12. Jelinek GA. Widow spider envenomation (latrodectism): A worldwide problem. Wild Environ Med. 1997;8 4:226-31. doi:Doi 10.1580/1080-6032(1997)008[0226:Wselaw]2.3.Co;2.
13. Wang XC, Tang XC, Xu DH and Yu DAM. Molecular basis and mechanism underlying the insecticidal activity of venoms and toxins from Latrodectus spiders. Pest Manag Sci. 2019;75 2:318-23. doi:10.1002/ps.5206.
14. Yan S and Wang XC. Recent Advances in Research on Widow Spider Venoms and Toxins. Toxins. 2015;7 12:5055-67. doi:10.3390/toxins7124862.
15. Akhunov AA, Golubenko Z, Abdurashidova NA, Mustakimova EC, Ibragimov FA and Mackessy S. Comparative biochemistry of the physiologically active components of venom, hemolymph, and eggs of the karakurt spider (Latrodectus tredecimguttatus). Chem Nat Compd+.

2001;37 6:562-5. doi:Doi 10.1023/A:1014829218721.

16. D.C. B and F.E. R. A poison from the eggs and spiderlings of the black widow spider. 1971.
17. C. BD, E. RF and A. D. Preliminary studies on the toxicity of black widow spider eggs. 1971;9 4:393-402.
18. Gendreau KL, Haney RA, Schwager EE, Wierschin T, Stanke M, Richards S, et al. House spider genome uncovers evolutionary shifts in the diversity and expression of black widow venom proteins associated with extreme toxicity. Bmc Genomics. 2017;18 doi:ARTN 178 10.1186/s12864-017-3551-7.
19. Argintean S, Chen J, Kim M and Moore AMF. Resilient silk captures prey in black widow cobwebs. Appl Phys a-Mater. 2006;82 2:235-41. doi:10.1007/s00339-005-3430-y.
20. Berger CA, Brewer MS, Kono N, Nakamura H, Arakawa K, Kennedy SR, et al. Shifts in morphology, gene expression, and selection underlie web loss in Hawaiian Tetragnatha spiders. Bmc Ecol Evol. 2021;21 1 doi:ARTN 48 10.1186/s12862-021-01779-9.
21. Stellwagen SD and Renberg RL. Toward Spider Glue: Long Read Scaffolding for Extreme Length and Repetitious Silk Family Genes AgSp1 and AgSp2 with Insights into Functional Adaptation. G3-Genes Genom Genet. 2019;9 6:1909-19. doi:10.1534/g3.119.400065.
22. Blasingame E, Tuton-Blasingame T, Larkin L, Falick AM, Zhao L, Fong J, et al. Pyriform Spidroin 1, a Novel Member of the Silk Gene Family That Anchors Dragline Silk Fibers in Attachment Discs of the Black Widow Spider, Latrodectus hesperus. J Biol Chem. 2009;284 42:29097-108. doi:10.1074/jbc.M109.021378.
23. Correa-Garhwal SM, Chaw RC, Clarke TH, Ayoub NA and Hayashi CY. Silk gene expression of theridiid spiders: implications for male-specific silk use. Zoology. 2017;122:107-14. doi:10.1016/j.zool.2017.04.003.
24. Babb PL, Lahens NF, Correa-Garhwal SM, Nicholson DN, Kim EJ, Hogenesch JB, et al. The Nephila clavipes genome highlights the diversity of spider silk genes and their complex expression. Nature genetics. 2017;49 6:895-903. doi:10.1038/ng.3852.
25. Sheffer MM, Hoppe A, Krehenwinkel H, Uhl G, Kuss AW, Jensen L, et al. Chromosome-level reference genome of the European wasp spider Argiope bruennichi: a resource for studies on range expansion and evolutionary adaptation. Gigascience. 2021;10 1 doi:10.1093/gigascience/giaa148.
26. Jain M, Olsen HE, Paten B and Akeson M. The Oxford Nanopore MinION: delivery of nanopore sequencing to the genomics community (vol 17, 239, 2016). Genome Biol. 2016;17 doi:ARTN 256 10.1186/s13059-016-1122-x.
27. Eagen KP. Principles of Chromosome Architecture Revealed by Hi-C. Trends Biochem Sci. 2018;43 6:469-78. doi:10.1016/j.tibs.2018.03.006.
28. Vurture GW, Sedlazeck FJ, Nattestad M, Underwood CJ, Fang H, Gurtowski J, et al. GenomeScope: fast reference-free genome profiling from short reads. Bioinformatics. 2017;33 14:2202-4. doi:10.1093/bioinformatics/btx153 %J Bioinformatics.
29. Hu J: https://github.com/Nextomics/NextDenovo (2020). Accessed February 24 2021.
30. Dudchenko O, Batra SS, Omer AD, Nyquist SK, Hoeger M, Durand NC, et al. De novo assembly of the Aedes aegypti genome using Hi-C yields chromosome-length scaffolds. Science (New York, NY). 2017;356 6333:92-5. doi:10.1126/science.aal3327.
31. Simao FA, Waterhouse RM, Ioannidis P, Kriventseva EV and Zdobnov EM. BUSCO: assessing genome assembly and annotation completeness with single-copy orthologs. Bioinformatics.

2015;31 19:3210-2. doi:10.1093/bioinformatics/btv351.

32. Li H and Durbin R. Fast and accurate short read alignment with Burrows-Wheeler transform. Bioinformatics. 2009;25 14:1754-60. doi:10.1093/bioinformatics/btp324.

33. Chang Z, Li GJ, Liu JT, Zhang Y, Ashby C, Liu DL, et al. Bridger: a new framework for de novo transcriptome assembly using RNA-seq data. Genome Biol. 2015;16 doi:ARTN 30 10.1186/s13059-015-0596-2.

34. Kielbasa SM, Wan R, Sato K, Horton P and Frith MC. Adaptive seeds tame genomic sequence comparison. Genome Res. 2011;21 3:487-93. doi:10.1101/gr.113985.110.

35. Krzywinski M, Schein J, Birol I, Connors J, Gascoyne R, Horsman D, et al. Circos: An information aesthetic for comparative genomics. Genome Res. 2009;19 9:1639-45. doi:10.1101/gr.092759.109.

36. Benson G. Tandem repeats finder: a program to analyze DNA sequences. Nucleic Acids Research. 1999;27 2:573-80.

37. Bedell JA, Korf I and Gish W. MaskerAid: a performance enhancement to RepeatMasker. Bioinformatics. 2000;16 11:1040-1. doi:DOI 10.1093/bioinformatics/16.11.1040.

38. RepeatModeler: http://www.repeatmasker.org/RepeatModeler/. Accessed December 27 2019.

39. Chalopin D, Naville M, Plard F, Galiana D and Volff JN. Comparative analysis of transposable elements highlights mobilome diversity and evolution in vertebrates. Genome Biol Evol. 2015;7 2:567-80. doi:10.1093/gbe/evv005.

40. Kapusta A: Parsing-RepeatMasker-Outputs. https://github.com/4ureliek/Parsing-RepeatMasker-Outputs. Accessed June 10 2019.

41. Stanke M and Waack S. Gene prediction with a hidden Markov model and a new intron submodel. Bioinformatics. 2003;19:Ii215-Ii25. doi:10.1093/bioinformatics/btg1080.

42. Sanggaard KW, Bechsgaard JS, Fang XD, Duan JJ, Dyrlund TF, Gupta V, et al. Spider genomes provide insight into composition and evolution of venom and silk (vol 5, 3765, 2014). Nat Commun. 2014;5 doi:ARTN 4590 10.1038/ncomms5590.

43. Kono N, Nakamura H, Ohtoshi R, Moran DAPE, Shinohara A, Yoshida Y, et al. Orb-weaving spider Araneus ventricosus genome elucidates the spidroin gene catalogue. Sci Rep-Uk. 2019;9 doi:ARTN 8380 10.1038/s41598-019-44775-2.

44. Schwager EE, Sharma PP, Clarke T, Leite DJ, Wierschin T, Pechmann M, et al. The house spider genome reveals an ancient whole-genome duplication during arachnid evolution. Bmc Biol. 2017;15 doi:ARTN 62 10.1186/s12915-017-0399-x.

45. Liu SL, Aagaard A, Bechsgaard J and Bilde T. DNA Methylation Patterns in the Social Spider, Stegodyphus dumicola. Genes-Basel. 2019;10 2 doi:ARTN 137 10.3390/genes10020137.

46. Babb PL, Lahens NF, Correa-Garhwal SM, Nicholson DN, Kim EJ, Hogenesch JB, et al. The Nephila clavipes genome highlights the diversity of spider silk genes and their complex expression. Nature genetics. 2017;49 6:895-+. doi:10.1038/ng.3852.

47. Birney E and Durbin R. Using GeneWise in the Drosophila annotation experiment. Genome Res. 2000;10 4:547-8. doi:DOI 10.1101/gr.10.4.547.

48. Kent WJ. BLAT - The BLAST-like alignment tool. Genome Res. 2002;12 4:656-64. doi:10.1101/gr.229202.

49. Haas BJ, Delcher AL, Mount SM, Wortman JR, Smith RK, Hannick LI, et al. Improving the Arabidopsis genome annotation using maximal transcript alignment assemblies. Nucleic Acids Research. 2003;31 19:5654-66. doi:10.1093/nar/gkg770.

50. Haas BJ, Salzberg SL, Zhu W, Pertea M, Allen JE, Orvis J, et al. Automated eukaryotic gene structure annotation using EVidenceModeler and the program to assemble spliced alignments. Genome Biol. 2008;9 1 doi:ARTN R7 10.1186/gb-2008-9-1-r7.
51. Zdobnov EM and Apweiler R. InterProScan - an integration platform for the signature-recognition methods in InterPro. Bioinformatics. 2001;17 9:847-8. doi:DOI 10.1093/bioinformatics/17.9.847.
52. Kanehisa M, Goto S, Kawashima S and Nakaya A. The KEGG databases at GenomeNet. Nucleic Acids Research. 2002;30 1:42-6. doi:DOI 10.1093/nar/30.1.42.
53. Altschul SF, Gish W, Miller W, Myers EW and Lipman DJ. Basic local alignment search tool. Journal of molecular biology. 1990;215 3:403-10. doi:10.1016/s0022-2836(05)80360-2.
54. Li L, Stoeckert CJ and Roos DS. OrthoMCL: Identification of ortholog groups for eukaryotic genomes. Genome Res. 2003;13 9:2178-89. doi:10.1101/gr.1224503.
55. Stamatakis A. RAxML version 8: a tool for phylogenetic analysis and post-analysis of large phylogenies. Bioinformatics. 2014;30 9:1312-3. doi:10.1093/bioinformatics/btu033.
56. Yang ZH. PAML: a program package for phylogenetic analysis by maximum likelihood. Comput Appl Biosci. 1997;13 5:555-6.
57. Kumar S, Stecher G, Suleski M and Hedges SB. TimeTree: A Resource for Timelines, Timetrees, and Divergence Times. Mol Biol Evol. 2017;34 7:1812-9. doi:10.1093/molbev/msx116.
58. Kumar S, Tamura K and Nei M. Mega - Molecular Evolutionary Genetics Analysis Software for Microcomputers. Comput Appl Biosci. 1994;10 2:189-91.
59. Takezaki N, Rzhetsky A and Nei M. Phylogenetic Test of the Molecular Clock and Linearized Trees. Mol Biol Evol. 1995;12 5:823-33.
60. Wood DLA, Miljenovic T, Cai SZ, Raven RJ, Kaas Q, Escoubas P, et al. ArachnoServer: a database of protein toxins from spiders. Bmc Genomics. 2009;10 doi:Artn 375 10.1186/1471-2164-10-375.
61. Haney RA, Ayoub NA, Clarke TH, Hayashi CY and Garb JE. Dramatic expansion of the black widow toxin arsenal uncovered by multi-tissue transcriptomics and venom proteomics. Bmc Genomics. 2014;15 doi:Artn 366 10.1186/1471-2164-15-366.
62. ClanTox. http://www.clantox.cs.huji.ac.il/index.php. Accessed December 1 2021.
63. Mistry J, Finn RD, Eddy SR, Bateman A and Punta M. Challenges in homology search: HMMER3 and convergent evolution of coiled-coil regions. Nucleic Acids Research. 2013;41 12 doi:ARTN e121 10.1093/nar/gkt263.
64. Stockholm. https://novopro.cn/tools/format-conversion.html. Accessed December 1 2021.
65. MG2C. http://mg2c.iask.in/mg2c_v2.1/. Accessed December 1 2021.
66. Letunic I and Bork P. Interactive Tree Of Life (iTOL) v5: an online tool for phylogenetic tree display and annotation. Nucleic Acids Research. 2021;49 W1:W293-W6. doi:10.1093/nar/gkab301.
67. iTOL. https://itol.embl.de/. Accessed June 1 2021.
68. Zhang Z, Xiao JF, Wu JY, Zhang HY, Liu GM, Wang XM, et al. ParaAT: A parallel tool for constructing multiple protein-coding DNA alignments. Biochem Bioph Res Co. 2012;419 4:779-81. doi:10.1016/j.bbrc.2012.02.101.
69. Wang D, Zhang Y, Zhang Z, Zhu J and Yu J. KaKs_Calculator 2.0: A Toolkit Incorporating Gamma-Series Methods and Sliding Window Strategies. Genomics, Proteomics & Bioinformatics. 2010;8 1:77-80. doi:https://doi.org/10.1016/S1672-0229(10)60008-3.

70. NCBI database. ftp://ftp.ncbi.nih.gov/blast/db. Accessed June 1 2021.
71. Zhao Y, Ayoub NA and Hayashi CY. Chromosome Mapping of Dragline Silk Genes in the Genomes of Widow Spiders (Araneae, Theridiidae). PLoS ONE. 2010;5 9:e12804-.
72. Li WH, Wu CI and Luo CC. A New Method for Estimating Synonymous and Nonsynonymous Rates of Nucleotide Substitution Considering the Relative Likelihood of Nucleotide and Codon Changes. Mol Biol Evol. 1985;2 2:150-74.
73. Kulkarni S, Kallal RJ, Wood H, Dimitrov D, Giribet G and Hormiga G. Interrogating Genomic-Scale Data to Resolve Recalcitrant Nodes in the Spider Tree of Life. Mol Biol Evol. 2021;38 3:891-903. doi:10.1093/molbev/msaa251.
74. Torkkeli PH, Panek I and Meisner S. Ca2+/calmodulin-dependent protein kinase II mediates the octopamine-induced increase in sensitivity in spider VS-3 mechanosensory neurons. Eur J Neurosci. 2011;33 7:1186-96. doi:10.1111/j.1460-9568.2011.07624.x.
75. Bhere KV, Haney RA, Ayoub NA and Garb JE. Gene structure, regulatory control, and evolution of black widow venom latrotoxins. FEBS Lett. 2014;588 21:3891-7. doi:10.1016/j.febslet.2014.08.034.
76. La Mattina C, Reza R, Hu X, Falick AM, Vasanthavada K, McNary S, et al. Spider minor ampullate silk proteins are constituents of prey wrapping silk in the cob weaver Latrodectus hesperus. Biochemistry. 2008;47 16:4692-700. doi:10.1021/bi800140q.
77. Vienneau-Hathaway JM, Brassfield ER, Lane AK, Collin MA, Correa-Garhwal SM, Clarke TH, et al. Duplication and concerted evolution of MiSp-encoding genes underlie the material properties of minor ampullate silks of cobweb weaving spiders. BMC Evol Biol. 2017;17 1:78. doi:10.1186/s12862-017-0927-x.
78. Wang Z, Zhu K, Li H, Gao L, Huang H, Ren Y, et al. Supporting data for "Chromosome□level genome assembly of the black widow spider Latrodectus elegans illuminates composition and evolution of venom and silk proteins" GigaScience Database. 2022; doi:http://dx.doi.org/10.5524/102210.

Figure legends:

Figure 1. Genome assembly and comparative analysis of the *L. elegans*. A. Heatmap of chromosome interactions in *L. elegans*. **B.** Circos plot of distribution of the genomic elements in *L. elegans*. From the outer ring to the inner ring are the distributions of protein-coding genes, tandem repeats (TRs), long tandem repeats (LTRs), short/long interspersed nuclear elements (SINEs/LINEs), DNA elements and GC content, respectively. **C.** Genomic synteny between *S. dumicola* and *L. elegans*. **D.** Genomic synteny between *P. tepidariorum* and *L. elegans*. **E.** Genomic synteny between *T. clavipes* and *L. elegans*.

Figure 2. Comparative genomics of *L. elegans* and related species. A. Phylogenetic relationships among these species. The red dot at the node represents a fossil record that was used for the calibration of the divergence time. The blue number in each node represents its divergence time for species. The red and green numbers in each node/species represent the expanded/contracted gene families, respectively. **B.** Comparison of the insertion history of transposable elements among species. The x-axis represents the inferred insertion time (unit: million years ago) of transposable elements in the genome. The y-axis represents the total/each length of the transposable element in each species.

Figure 3. Toxin genes in the Arachnida species. A. Different toxin gene numbers in these species. **B.** The distribution of toxin genes in *L. elegans*. **C.** The phylogenetic relationship of latrotoxins genes in these species. **D.** Latrotoxin gene Ka/Ks value of these species. The latrotoxin gene in *I. scapularus* was used as the reference.

Figure 4. Spidron genes in the Arachnida species. A. Different spidron gene numbers in three spider species. **B.** The chromosome distribution of spidron genes in *L. elegans*.

Figure1

Click here to access/download;Figure;Figure 1-new.pdf

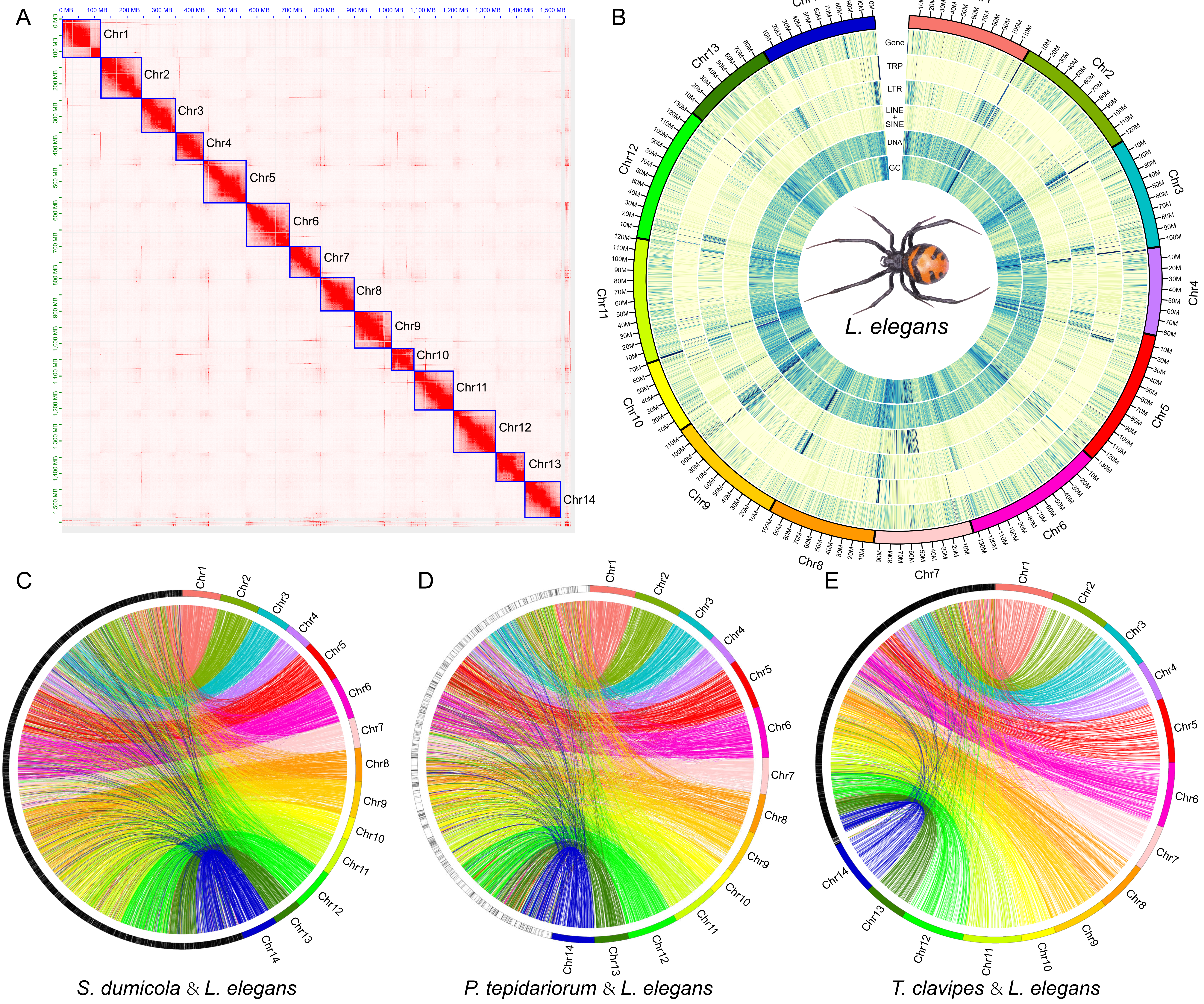

Figure2

Click here to access/download;Figure;Figure 2-new.pdf

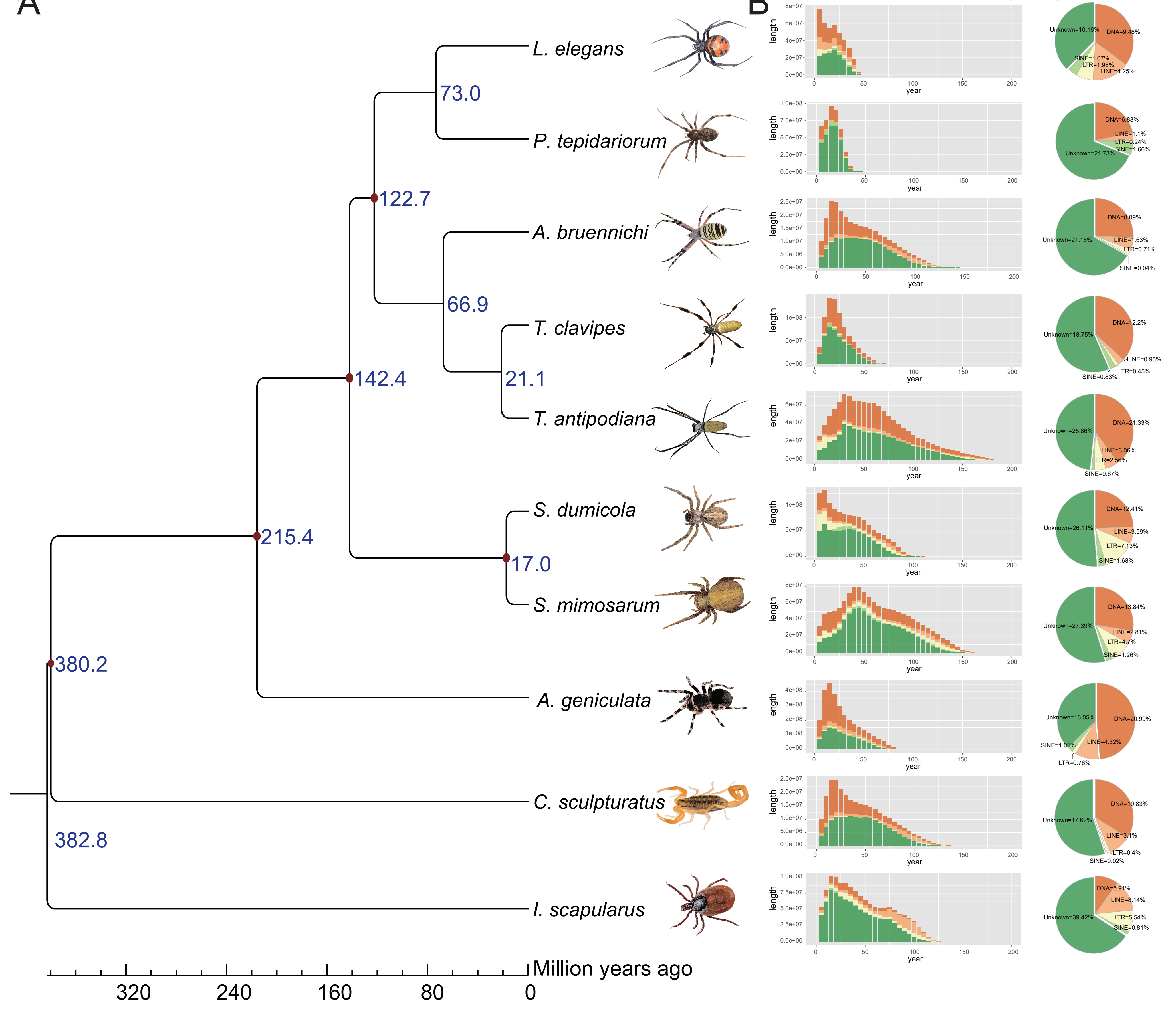

Figure3

Click here to access/download;Figure;Figure 3-new.pdf

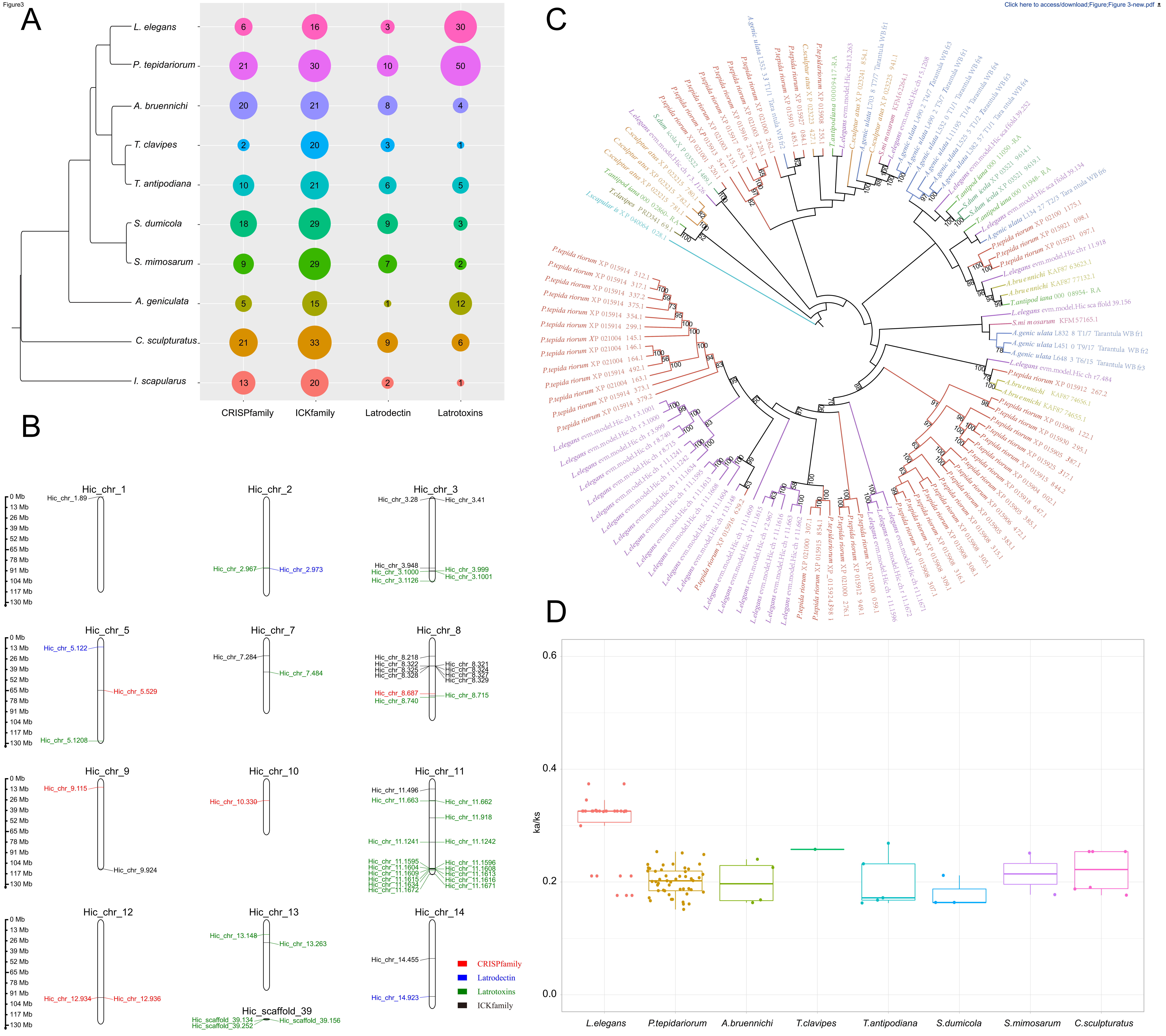

Click here to access/download;Figure;Figure 4.pdf

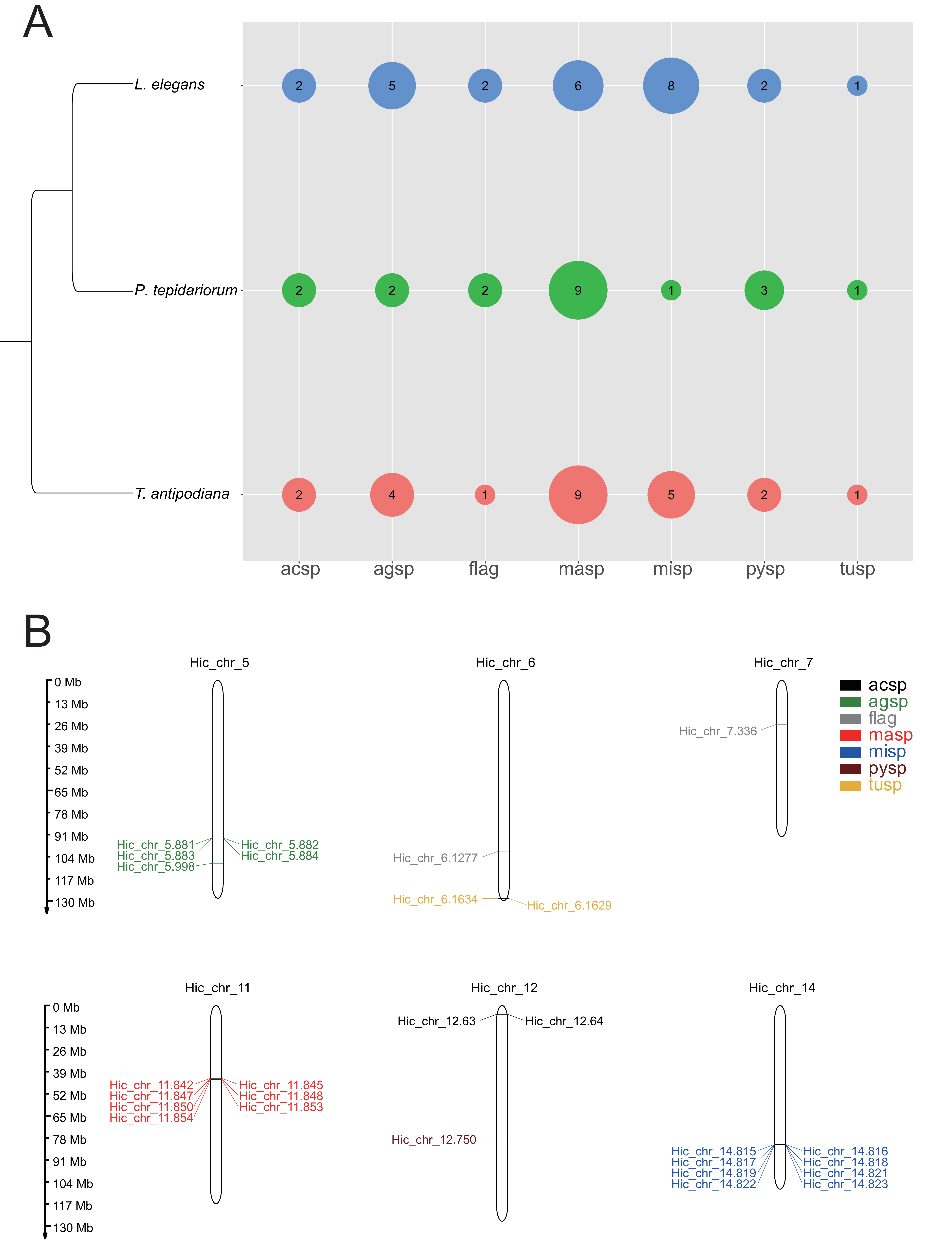

Click here to access/download
Supplementary Material
Supplementary tables & figures-0220-combine.docx

 Click here to access/download;Personal Cover;BlackwidowGigaMS_response letter-0222xh.docx

Dear Editor,
Thank you and the three reviewers for your time and constructive comments on our manuscript " Chromosome-level genome assembly of the black widow spider *Latrodectus elegans* illuminates composition and evolution of venom and silk proteins" (GIGA-D-21-00338). These comments will greatly help improving our manuscript. Now we carefully revised our manuscript according to the reviewers' comment and polish the grammar in our revised manuscript. We wish that the updated manuscript could fit the reviewers' requirement. Meantime, we made the point-by-point responses to each of the comment from the editor and the reviewers, as follows:

Editor:
Please also take a moment to check our website at https://www.editorialmanager.com/giga/ for any additional comments that were saved as attachments.

R: we checked the website and found no additional comments. Thank you for your kindly reminding.

In addition, please register any new software application in the bio.tools and SciCrunch.org databases to receive RRID (Research Resource Identification Initiative ID) and biotoolsID identifiers, and include these in your manuscript. This will facilitate tracking, reproducibility and re-use of your tool.

R: Thank you for your reminding. We checked all the softwares used in our manuscript and confirmed that there was no any new software used. Meantime, the electronic notebook-style methods document of these softwares were upload to the GigaDB dataset. We affirm that our tools are easy to track and reproducible.

Please ensure you describe additional experiments that were carried out and include a detailed rebuttal of any criticisms or requested revisions that you disagreed with. Please also ensure that your revised manuscript conforms to the journal style, which can be found in the Instructions for Authors on the journal homepage. If the data and code has been modified in the revision process please be sure to update the public versions of this too.

R: We have ensured all the above items.

Besides, we suggest you find a copy editing company or friendly native English speaker to polish the grammar.

R: We have made our manuscript polished by a copy editing company (Letpub).

Reviewer reports:
Reviewer #1: The manuscript described the genome of an orb-weaver spider *Latrodectus elegans* with a combination of Nanopore, Illumina and Hi-C sequencing

and the comparison of latrotoxin genes and spidroin genes among spiders. The authors reported the assembly, quality and analysis of the genome in details and provided enormous information, in my opinion, useful to the research community. However, I suggest a major revision before the work is published. The main concern is on the toxin identification.

R: Thank you very much for your overall positive comments to our manuscript and important suggestion.

I have one major concern related to the analysis of venom toxin genes. In Fig. 3 and related text, no ICK family gene was identified in five species including spider, scorpion and tick where ICK toxins have been reported and even functionally studied. In the other five spider species, fewer ICK genes were found (1 to 7). ICK family was considered an abundant toxin family in Arachnida and their homology is mainly dependent on the disulfide-bond instead of the primary sequence. The toxin genes were identified via blastp, which heavily relies on the sequence similarity. Similar concerns may apply to the other toxin families. I would suggest the authors redo the toxin gene analysis.

R: Thanks for your important comments and we are sorry for our carelessness in spider toxin identification. We investigated more on these toxins and learned that, as the reviewer stated, ICK toxins are a kind of small peptide and their homology is mainly dependent on the disulfide-bond instead of the primary sequence, unlike those proteins from CRISP family or Latrotoxin family. We then re-identified the ICK toxins through the following approaches. Firstly, we retrieved the peptides less than 200 amino acid and have at least six cysteines constituting the NCNCNCCNCNC (N refers to any other amino acid) motif. Secondly, we used the online tool ClanTox (http://www.clantox.cs.huji.ac.il/index.php) to evaluate the toxin potential of the above candidate ICK toxins. Thirdly, we used SignalP (v5.0) to predict signal peptide of them and those without signal peptide was removed out. After further manual check, we finally identified the ICK toxins. As to Latrodectin family genes, there are rather few genes in ArachnoServer 3.0 database. We then retrieved the known sequences of Latrodectin from NCBI, then we applied the Blastp with e value cutoff of 10-5 to search the candidate homolog proteins. Then the homolog was further verified by hmmer (v3.3). In brief, all above-found sequences were merged together and applied Clustalw2 to perform the multiple sequence alignment which were then transformed into stockholm format using online toolset (https://novopro.cn/tools/format-conversion.html). The aligned sequences were used to construct the HMM profile using hmmbuild, a binary software of hmmer (v3.3). Finally, hmmsearch was applied for the peptide of each species, to find the potential domains of all species using key parameters "—domtblout and e-value of 10-5".

Based on the updated methods, we did identify more ICK toxins. We updated the related methods and the results in the revised manuscript.

Minor errors:

1. Line 36, "remarkably more MiSp". It is grossly overwritten.

R: Thanks for reminding. We removed “remarkably” to downplay the statement.
2. Line 345-346, any reference?

R: We added a reference “Bhere KV, Haney RA, Ayoub NA and Garb J E. Gene structure, regulatory control, and evolution of black widow venom latrotoxins. FEBS Lett 2014; 588(21): 3891-3897.” therein. Thanks for reminding.
3. Line 368, "We used the same method". What is the method? Same to what? There is not description in the above sentences.

R: Sorry for this rather poor writing. We removed these statements since we have described the method of spidroin genes in the Methods section. We modified the statements to “We identified six unique annotated genes for MaSp, eight for MiSp, two for Flag, five for AgSp, two for PySp, two for AcSp and one for TuSp in *L. elegans*”.
4. Many typo are present in the main text. The manuscript could be improved by carefully revising the text and reformatting the reference list.

R: We are sorry for our carelessness. We carefully revised the main test and had let a copy editing company (Letpub) polish the grammar.

Reviewer #2: In this manuscript, the authors present a high-quality genome assembly of the species *Latrodectus elegans*, based on nanopore reads and HI-C scaffolding. In addition, they present the most relevant characteristics of the genome and its functional annotation, as well as a phylogenetic analysis that includes other arachnids, estimates of the evolutionary rate in different lineages, and focused on the chromosomal organization and evolution of some gene families of interest, such as toxins and spidroins.

I think the work is very interesting. This new genome provides new data that will be very useful to advance our understanding of the diversification of spiders and the evolution of some of the most relevant aspects of their biology. Nevertheless, I have some important concerns about different aspects of the ms, which in my opinion must be properly addressed before considering the manuscript suitable for publication in Gigascience.

R: Thank you very much for your interest of our manuscript and the instructive comments.

First of all, I think it is necessary to improve the language. I am not a native English speaker, but I think there are many sentences that are not well punctuated and some expressions are not well constructed. I highly recommend a thorough review of the entire text.

R: We are very sorry for our poor writing. We made a carefully revision and modification in writing. In addition, we had let a copy editing company polish our manuscript, in order to further improve it.

Apart from the language itself, there are poorly constructed sentences from the point of view of biological concepts. Phrases such as "Systematic identification of complete toxin gene sequence", in line 68, or "..will aid the identification and understanding of the complete spider toxin and spider silk genes..line 76", or "..to detect the genome integrity..line 117", or "transposon elements..line 121"..or "connected into one super gene..line 169", or "all merged values were plotted and grouped by species...line 210", or "the chromosome distribution of spidroin were plotted...line 229", or "..genes that had homolog genes in the public database..line 264", or "the original group of arachnida..line 327"..etc..

R: Line 68: we modified it to: "Systematic identification and analysis of all the spider toxin genes with full length sequences".

Line 76: we modified it to: "…will provide important resources for deciphering the spider toxin and spidroin genes".

Line 117: we modified it to: "..to evaluate the genome integrity."

Line 121: we modified it to: "transposable elements".

Line 169: we modified the sentence to: "All the well-aligned single-copy orthologous genes in each species were concatenated to one super gene for each species."

Line 210: we modified it to: "all the Ka/Ks values were plotted and grouped by species." We also made other modifications in the context.

Line 229: we modified it to: "The chromosome distribution of the spidroins were then plotted ...".

Line 264: we modified it to: "... genes having homologous hits in the public databases."

Line 327: we modified it to "... It is the only species from the relatively ancient Mygalomorphae. The result suggests that relatively ancient spider group may have relatively less selection pressure in their habitats. However, due to its low genome quality, this result is pending further verification....".

In addition, we carefully checked the whole manuscript and made intensive revisions of it.

Regarding specific contents, there are some things that need to be revised, rewritten or expanded. Here are some examples:
In line 137 a sentence begins with "the protein sequences of ...." and then, after the comma, continues with "... the longest transcript were chosen for further analyzes. Something is wrong here ...

R: We modified the statement to: "We chose the longest isoform of each gene to obtain the non-redundant protein sequences of each gene set." We also made modifications in the context.

In line 143, the phrase "All the remaining genes were aligned using blastn" is not understood, what do the authors mean?

R: We modified the related statements to: “We chose the longest isoform of each gene to obtain the non-redundant protein sequences of each gene set. The protein sequences were used as query to search for orthologous regions in the *L. elegans* genome by tblastn with an e-value of 1e-5. The results were subjected to GeneWise software (v2.4.1) (Birney and Durbin 2000) to predict gene structure.”

Various lines. The names and identifiers of the species used in the different analyzes are unnecessarily repeated in several paragraphs.

R: Thanks for reminding. We carefully checked the manuscript and removed the repeated names and identifiers of the species.

line 150. Please, replace "homologous searching" with "sequence similarity-based searches" or similar...

R: Thanks. We replaced "homologous searching" with" homology-based searches".

Line 183. "Using ..... gene pairwise relationships". What do the authors mean by this phrase? What pairwise relationships do they use to determine the evolution of families in CAFE?

R: We are sorry for this vague description. However, in order to limiting genomic analysis and make the manuscript meet the requirement of “Date note” in Gigascience, we removed unnecessary genomic analysis, including analysis on gene family expansion and contraction. So this sentence was removed.

line 190. "To identify the potential positively selected genes (PSGs) and the genes with positively selected sites ..." I do not understand the difference between these two classes of genes.

R: Sorry that we didn’t describe it clear. We used different models in Codeml tool of PAML package (v4.8) to detect potential positively selected genes (PSGs) and the genes with positively selected sites. PSGs were those identified by two ratio branch model. Those not identified as PSGs, were then subjected to branch site model to detect whether they have positive selected sites. We re-wrote the method of positive selection analysis: “Single-copy orthologs from the five relatively closely related species of orb-web weaving spiders, i.e., *L. elegans*, *P. tepidariorum*, *A. bruennichi*, *T. clavipes* and *T. antipodiana*. As well as their species tree were used to identify the potential positively selected genes (PSGs) and the genes with positively selected sites in *L. elegans*, using branch model and branch site model in the Codeml tool of the PAML package (v4.8) (Yang 1997) respectively. Briefly, the rate ratio (ω) of nonsynonymous to synonymous nucleotide substitutions was estimated. One ratio model was used to detect average ω across the species tree (ω0). For each gene, two ratio branch model was used to detect the ω of the appointed branch (*L. elegans*) to test the (ω1) and ω of all other branches (ω_background). A likelihood ratio test was performed to compare the fit of the two ratio models with the one ratio model to determine whether the gene is positively selected in the appointed branch ($\omega1>\omega0$; $\omega1>\omega\_background$; $\omega1>1$; $P\text{-value}<0.05$). If the gene is not positively selected, then the branch site model was used to detect amino acid sites likely to be is positively

selected in the appointed branch using Bayes Empirical Bayes (BEB) analysis (Yang et al., 2005). ”
Line 229. It should say "distribution of spidroins" ...
R: Thanks and we revised it.
In material and methods, for example, much information is lacking on how or with what software some things have been done. For example, the exact location where the sample was obtained is missing, now only the province is specified (line 87). Authors do not specify where the sequencing has been done (lines 91 and 92). The software used to filter the nanopore reads is missing (lines 96 and 97). Are the Perl scripts used written specifically for the project (in house-scripts) or are part of other software (line 96)?. It is not very clear for me what the Kimura value means (line 130), is it the corrected evolutionary distance using a kimura model? and, if true, which model, 2P or 3P?). Is the r8s program really implemented in the PAML package? (line 132).
R: **Line 87:** the spider is collected in Binzhou county, Dali City, Yunnan province.
Lines 91 and 92: we sent the live sample to Beijing Biomarker Technologies. Co. LTD for sequencing.
Lines 96 and 97: the Perl script used for filter the Nanopore reads is written specifically for this project (in house-script). We added it in our electronic notebook-style methods document and upload to the GigaDB dataset.
Line130: Yes, Kimura value is the corrected evolutionary distance using a kimura model. We used the parseRM (https://github.com/ 4ureliek/Parsing-RepeatMasker-Outputs) to conduct the kimura-distance based analysis on TE insertion time. We checked the pipeline and confirmed that 2P model is used therein. The principle is from the reference: Chalopin, D., Naville, M., Plard, F., Galiana, D., and Volff, J.-N. (2015). Comparative analysis of transposable elements highlights mobilome diversity and evolution in vertebrates. Genome Biol. Evol. 7, 567–580.
Line 132: We are sorry for this mistake. Yes, r8s is an individual program. Given that we re-wrote the description of TE analysis (The insertion time of each TE sequence was estimated using a Kimura distance based analysis (Chalopin, Naville et al. 2015) using parseRM (https://github.com/ 4ureliek/Parsing-RepeatMasker-Outputs)), this sentence was removed.
We added updated and revised information to the revised manuscript.

Other concerns:
The paragraphs titled Relative evolution calculation and Gene family expansion and contraction in the methods section are poorly written, it is hard to understand what they are doing.
R: Sorry for the poor descriptions. However, in order to limiting genomic analysis and make the manuscript meet the requirement of “Date note” in Gigascience, we removed unnecessary genomic analysis, including analysis on gene family expansion and contraction. So this sentence was removed.
Lines 183-184. "to identify gene families significantly expanded or contracted"

R: Thanks. However, in order to limiting genomic analysis and make the manuscript meet the requirement of “Date note” in Gigascience, we removed unnecessary genomic analysis, including analysis on gene family expansion and contraction. So this sentence was removed.

In the paragraph titled Positive Selection Analysis in the methods section, please specify what specific evolutionary models have been used.

R: Sorry for the vagueness. We re-wrote these contents and clearly described the models used.

Why are different compilations of RAxML (AVX or PHREADS) used in different parts of the job?

R: In our manuscript, two different compilations were used because they were run on different clusters, which were “raxmlHPC-PTHREADS-AVX” and “raxmlHPC-PTHREADS”. These are in fact of no difference in performance for both are PTHREADS version, and the AVX complication means the processors support AVX vector instructions which is faster in operation. To satisfy the needs of different part of the work, we changed our processors and the corresponding raxml complications.

Were the 14 scaffolds obtained in the assembly expected?

R: Yes, they were. The female black widow spiders are reported to have 28 chromosomes.

Is there information on karyotypes of this species?

R: Although there is no karyotype of this species, there is evidence that female black widow spiders have 28 chromosomes. Thanks for your reminding and we added the reference (Zhao YN, Ayoub A, Hayashi CY. Chromosome mapping of dragline silk genes in the genomes of widow spiders (Araneae, Theridiidae). PLoS One 2010; 5(9): e12804.), and the related statement to the revised manuscript.

What software has been used to make the graphs of the syntenic relationships between assemblies? That information is not in methods.

R: Sorry for this problem, softwares Last (v1066) and Circos (v0.69-6) were used. To perform the whole genome synteny analysis, we implemented the last software (v1066) to perform the whole genome alignment using *L. elegans* assembly as database, in which the “lastal” command was first used to obtain MAF format alignment files and the “maf-swap” command was then used to sort the alignment and select the best one-to-one blocks. After that, Circos (v0.69-6) was used to plot the syntenic relationship graph. Thank you for your reminding, we have added it in the method part of the manuscript.

Line 277. CDS has not been defined previously. Where does the acronym 4dTV come from?

R: Sorry for the miss, we have added the definition of CDS in the manuscript. A nucleotide site is classified as nondegenerate, twofold degenerate, or fourfold degenerate (4dTV), depending on how often nucleotide substitutions will result in amino acid replacement, we have cited the literature in the manuscript.

Line 279. "High boostrap values" ... not results, results are not high.

R: Sorry for this poor description. We have corrected it in the manuscript.

To what extent is the phylogeny obtained in this work different from those already known? What is the contribution of this part of the work to the knowledge of the diversification of these species?
R: The phylogeny obtained in this work is consistent to previously reported pattern. We added information in the species in our study to spider tree of life, especially estimating the divergent time between it and the relative Theridiidae spider, *P. tepidariorum.*
In the paragraph titled "Gene family expansion and contraction of *L. elegans*" of results and discussion (see comment on lines 183-184 for a more correct title), the p-value <0.05, is a value corrected by the multiple testing the many families, or does it mean that the p value of each family is <0.05? Consider this problem if not. In fact, much information is lacking on how the turnover rate analysis has been done in this study. Please, expand the method section where this aspect is discussed.
R: Thanks for your comments. In order to limiting genomic analysis and make the manuscript meet the requirement of “Date note” in Gigascience, we removed unnecessary genomic analysis, including analysis on gene family expansion and contraction, and highlighted analyses on toxin and spidroin genes.
Lines 304-307. Please explain the reason for this statement or provide references.
R: Thanks for your comments. In order to limiting genomic analysis and make the manuscript meet the requirement of “Date note” in Gigascience, we removed unnecessary genomic analysis, including analysis on gene family expansion and contraction. So this content was removed.
Line 311. A. geniculata is not an ancient spider! Perhaps the authors mean that it belongs to an ancient lineage.
R: We are very sorry for this mistake. Yes, we could not say *A. geniculata* is an ancient spider. This species belongs to Theraphosidae of the ancient Mygalomorphae, a relatively ancient lineage of Araneae. However, in order to limiting genomic analysis and make the manuscript meet the requirement of “Date note” in Gigascience, we removed unnecessary genomic analysis, including general analysis on homologs. So this sentence was removed.
Lines 310-311. "This means that most of the genes have orthologous genes ..." How does this phrase match the fact that the phylogeny has only been done with 156 single-copy genes? Are all the other families with other orthology relationships?
R: In the area of evolutionary genomics, homology genes are clustered into two parts, including ortholog and paralog. Especially, the former one, ortholog genes, are clustered mainly into single copy and multi copy parts. In the analysis of OrthoMCL, a classical gene family clustering software, five types are plotted, including single copy orthologs, multiple copy orthologs, other orthologs, unique paralogs, and unclustered genes. Considering the genome quality of *A. geniculate*, it is not figured into this description. That means most of the genes have orthologous genes, but only 156 genes are clustered into single-copy orthologs.

However, in order to limiting genomic analysis and make the manuscript meet the requirement of “Date note” in Gigascience, we removed unnecessary genomic analysis, including general analysis on homologs. So this sentence was removed.

Line 320-321. Could it be the result of differences in the quality of annotations and assemblies rather than unique characters of this species?

R: Obviously, we could not exclude the possibilities. These could be caused by a lot of reasons, like sequencing error, assembly error, and even the computational errors of the servers or the clusters. However, based on the big data analysis, and the rapid development of sequencing and software technology, especially, our fully comparative genomics and evolutionary genomics analysis, we have sufficient confidence in the quality of our assembly and annotation.

However, in order to limiting genomic analysis and make the manuscript meet the requirement of "Date note" in Gigascience, we removed unnecessary genomic analysis, including general analysis on homologs. So this sentence was removed.

Line 327. ".this original group of arachnida ..", what does original mean? please review.

R: Sorry for the mistake. We revised the statements to: "Interestingly, *A. geniculata* had the slowest evolution rate. It is the only species from the relatively ancient Mygalomorphae. The result suggests that relatively ancient spider group may have relatively less selection pressure in their habitats".

Line 332. "...eight positively selected genes and 384 genes with positively selected sites ..". In both cases genes are positively selected. Please better explain the difference and specify which models have been used.

R: Sorry for the vagueness description. We re-wrote the method and clearly indicate how to distinguish positively selected genes (PSGs) and genes with positively selected sites. We added the information on model use in PAML therein.

line 343. "..we identified four major spider venom family proteins ..". This sentence is badly constructed. Perhaps the authors meant ... "We identified proteins / copies / members of (the) four major venom gene / protein families" ...

R: Sorry for the poor writing. We revised to: "We identified members of the four major venom gene families".

line 348. Please, indicate the p-value of these analyzes.

R: Thanks. However, considering to another review's comments, we need to limited unnecessary genomic analysis. We hence removed analysis on gene family expansion and contraction. Accordingly, we removed this sentence.

line 360. "We analyzed the nucleotide substitutions (Ks) .... and found that L. elegans laxotroxin genes had higher (Ka / Ks)". I don't understand the first (Ks).

R: We are sorry for this misleading typo. It is the Ka/Ks rather than Ks. We revised the sentence to: "We analyzed the ratio of nonsynonymous to synonymous nucleotide substitution rate (Ka /Ks ratio) of each laxotroxin gene pair and found that *L. elegans* laxotroxin genes had generally higher Ka/Ks ratios compared to those in other species,…" .

line 371. "relatively more ..." is not a particularly scientific expression.

R: We are sorry for this unprofessional expression. We removed "relatively".

Conclusions: I believe that many of the conclusions of this ms are not justified or are not supported by the results. They are more the result of speculation and interpretation.

They should focus more on the description of the genome and its characteristics. The last sentences should move to the introduction.
R: Sorry for our poor writing. We updated the conclusion:
"Using the approach combining data of Illumina short reads, Nanopore long reads and Hi-C, we assembled and annotated the first chromosome-level 1.57 Gb large genome of a black widow spider, *L. elegans*. In this study, we confirmed phylogenetic position of this species in the spider tree of life. In addition, by analysis on hox gene family, we again verified it high quality. Specifically, we focused on toxin and spidroin genes, which contribute to the distinctive features of black widow and cob web-weaving spiders and provided substantial information in terms of their composition and numbers and preliminarily demonstrate the evolution pattern of one important toxin gene family, latrotoxins. The important venom toxins contribute greatly to black widow spider's toxicity and they showed fast evolution. Generally, the genome resource data will help for deep exploration of spider genome evolution, specially on diversification of veom and web-forming. The sequence data are also first-hand templates for further application of the spider biomass."

Reviewer #3: Chromosome-level genome assembly of the black widow spider *Latrodectus elegans* illuminates composition and evolution of venom and silk proteins

This manuscript described the sequencing and assembly of a black widow spider, and a number of analyses comparing the obtained reference genome sequence to other spider genomes.
I realize that the manuscript is submitted as 'Data note', but a number of genome analyses are performed, and it is not very clear to me what the overall questions are that the authors want to address.
R：Thanks for your reminding. The species used in our study is a kind of black widow spider, with well-known of its venom. It is also featured with its distinctive web, which is three-dimensional and called cob-web. We hence provided more information on the venom toxins and spidroins of this species, in order to highlight the importance of the genome resource. We noticed that a recent data note published note on Gigascience showed similar kind of information (such as: Sheffer MM, Hoppe A, Krehenwinkel H, Uhl G, Kuss AW, Jensen L, Jensen C, Gillespie RG, Hoff KJ and Prost S. Chromosome-level reference genome of the European wasp spider *Argiope bruennichi*: a resource for studies on range expansion and evolutionary adaptation. Gigascience 2021;10: 1-12.). According to your comment, we limited unnecessary genomic analysis. For instances, we removed analysis on gene family expansion and contraction, as well as general analysis on homologs. We also made modifications of our manuscript and made it meet the format requirement of "Date Note".
The conclusion in the abstract is not a conclusion; just says 'this data is valuable', but not how/why.
R: We modified the whole content of the abstract. Specifically, the revised conclusion therein was: "We present the first chromosome-level genome assembly of a black widow spider and provided substantial toxin and spidroins gene resources. These high-qualified genomic data add valuable resources from a distinctive spider group

and contribute to deep exploration on spider genome evolution, especially in terms of the important issues on diversification of venom and web-weaving pattern. The sequence data are also first-hand templates for further application of the spider biomass."

Line 47: what does it mean 'due to their adaptability'? It sounds like they special with regard to adaptation. Is this so?

R: Sorry for the misleading statement. Mechanism underlying wide distribution of spiders is a big issue and could not simply be attributed to adaptability and diverse behavior. We cautiously removed this statement.

Line 73: the authors say 'full length spidroin sequences have been found' but no references. Here are some options:

-https://www.nature.com/articles/ncomms4765

-https://www.nature.com/articles/ng.3852

-https://academic.oup.com/gigascience/article/10/1/giaa148/6067174?login=true

R: We had cited two references which were shown in the end of the sentence. Thanks for providing the references. Since "https://www.nature.com/articles/ncomms4765": the spidroin sequences in this paper is not full-length, we added the other two papers to our revised manuscript.

Line 309: The A. geniculata genome is of much lower quality than the other genomes. Therefore, the conclusions given in this section should be removed. This should also apply to line 326.

R: Thanks for your reminding. In order to limiting genomic analysis and make the manuscript meet the requirement of "Date note" in Gigascience, we removed unnecessary genomic analysis, including general analysis on homologs. So this content was removed.

I think that you should cite the papers publishing all the spider genome sequences (plus I. scapularis and C. sculpturatus) that you download and analyze.

Generally, I think that the manuscript uses too few citations. Another example is line 256 where the authors say that the results of their hox gene analysis are similar to others species, but without any references. Actually, I do not think there is a single reference in the result/discussion section, but may have missed it/them!!!

R: Thanks for your reminding. We made intensive modifications in the manuscript and added quite a few new references to support our results and discussion. Line 256, we added the references: Schwager EE, Sharma PP, Clarke T, et al. The house spider genome reveals an ancient whole-genome duplication during arachnid evolution. BMC Biol. 2017; 15 (1):62. Published 2017 Jul 31. doi: 10.1186/s12915-017-0399-x; Sheffer MM, Hoppe A, Krehenwinkel H, et al. Chromosome-level reference genome of the European wasp spider *Argiope bruennichi*: a resource for studies on range expansion and evolutionary adaptation. Gigascience. 2021; 10 (1): giaa148. doi:10.1093/gigascience/giaa148; A chromosome-level genome of the spider *Trichonephila antipodiana* reveals the genetic basis of its polyphagy and evidence of an ancient whole-genome duplication event.

The figures are very busy with a lot of tiny text, and I have a hard time seeing that they read when fitted into a paper.

R: Sorry. We have enlarged the text to make the figures more readable.
