# Supplementary material for: Chromosome‐level genome assembly of the black widow spider Latrodectus elegans illuminates composition and evolution of venom and silk proteins
Source: Gigascience. 2022 May 25;11:giac049. doi: 10.1093/gigascience/giac049 (PMC9154082; doi:10.1093/gigascience/giac049)
Supplement: giac049_Supplemental_Tables_and_Figures [file giac049_supplemental_tables_and_figures.docx]

**Table S1. The statistics of sequencing reads on Illumina platform.** These data are produced by short insert library, and the results were shown by the raw sequencing reads. The sequencing depth was calculated by the 17-mer estimated genome size, genome is 1,740,446,424bp.

| Term | Read number | Total bases | Sequencing strategy | Sequencing depth (X) |
| --- | --- | --- | --- | --- |
| AG555-H01 | 443,246,170 | 66,486,925,500 | PE150 | 38.20 |
| Total | 443,246,170 | 66,486,925,500 | - | 38.20 |

**Table S2. The statistics of sequencing reads on Nanopore platform.** The reads with quality value Q > 7 were considered. The sequencing depth was calculated by the 17-mer estimated genome size.

| Lib-ID | Mean length | N50 length | Read number | Total base | Sequencing depth (X) |
| --- | --- | --- | --- | --- | --- |
| AG555-H02 | 17,199 | 24,013 | 6,209,788 | 106,803,527,887 | 61.37 |
| Total | 17,199 | 24,013 | 6,209,788 | 106,803,527,887 | 61.37 |

**Table S3. The statistics of Hi-C sequencing reads.** The sequencing depth was calculated by the 17-mer estimated genome size.

| Term | Read pairs | Total bases | Sequencing strategy | Sequencing depth (X) |
| --- | --- | --- | --- | --- |
| AG555-H03 | 519,021,422 | 77,575,233,122 | PE150 | 44.57 |
| Total | 519,021,422 | 77,575,233,122 | - | 44.57 |

**Table S4. The statistics of the polished genome and chromosome-level genome.** These data are produced by short insert library, and the results were shown by the raw sequencing reads. The polished genome was assembled by Nexedenovo and polished by Pilon. The chromosome-level genome is constructed by 3d DNA.

|  | **Polished genome** | | **Chromosome-level genome** | |
| --- | --- | --- | --- | --- |
| Term | Size (bp) | Number | Size (bp) | Number |
| N90 | 1,031,897 | 371 | 84,326,276 | 13 |
| N80 | 1,937,133 | 261 | 95,061,165 | 11 |
| N70 | 2,800,267 | 194 | 103,784,820 | 10 |
| N60 | 3,504,120 | 145 | 111,544,711 | 8 |
| N50 | 4,335,605 | 105 | 114,313,277 | 7 |
| Max length (bp) | 23,342,203 | - | 133,922,626 | - |
| Total size (bp) | 1,566,725,825 | - | 1,566,804,893 | - |
| Total number (>100 bp) | - | 933 | - | 164 |
| Total number (>10 kb) | - | 933 | - | 164 |

**Table S5. Statistics of the assembled chromosome-level genome via 3D *de novo* assembly software.**

| Chromosome ID | Length (bp)/Percentage (%) | Chromosome ID | Length (bp)/Percentage (%) |
| --- | --- | --- | --- |
| HiC_chr_1 | 119,656,895 | HiC_chr_8 | 103,784,820 |
| HiC_chr_2 | 124,379,975 | HiC_chr_9 | 114,313,277 |
| HiC_chr_3 | 106,775,008 | HiC_chr_10 | 70,395,454 |
| HiC_chr_4 | 84,326,276 | HiC_chr_11 | 120,392,845 |
| HiC_chr_5 | 132,398,706 | HiC_chr_12 | 131,035,894 |
| HiC_chr_6 | 133,922,626 | HiC_chr_13 | 88,694,972 |
| HiC_chr_7 | 95,061,165 | HiC_chr_14 | 111,544,711 |
| Total chromosome level scaffold length | | 1,536,682,624 | |
| Total length | | 1,566,804,893 | |
| Chromosome/total (%) | | 98.08% | |

**Table S6. The statistics of RNA sequencing reads on Illumina platform.** These data are produced by short insert library, and the results were shown by the filtered reads.

| Library | Organ/Tissue | Read number | Total bases | Sequencing strategy |
| --- | --- | --- | --- | --- |
| AG555-04 | Whole body | 50,049,380 | 7,475,372,554 | PE150 |
| Total | - | 50,049,380 | 7,475,372,554 | - |

**Table S7. The statistics of the assembled transcripts by Bridger of 5 organs/tissues.**

| Term | Size (bp) | Number |
| --- | --- | --- |
| N90 | 314 | 55,091 |
| N80 | 522 | 36,191 |
| N70 | 840 | 24,669 |
| N60 | 1,257 | 17,315 |
| N50 | 1,695 | 12,137 |
| Max length (bp) | 22,370 | - |
| Total size (bp) | 75,840,687 | - |
| Total number (>1 kb) | - | 21,417 |
| Total number (>10 kb) | - | 85 |

**Table S8. The statistics of the transcripts mapping ratio on the assembled genome.**

| Tissues/Organs | Mapping transcripts number | Total transcripts number | Mapping ratio (%) |
| --- | --- | --- | --- |
| Whole body | 77,191 | 85,772 | 90.00% |

**Table S9.** **The statistics of the short reads mapping ratio on the assembled genome.** PE mapped represent reads being mapped to the genome as read pairs, SE mapped represent reads being mapped to the genome as single reads.

| Terms | Reads number | Mapping ratio (%) |
| --- | --- | --- |
| Total Reads | 360,485,654 | - |
| Mapped Reads | 357,877,609 | 99.28% |
| PE mapped Reads | 347,225,730 | 96.76% |
| SE mapped Reads | 951,873 | 0.27% |

**Table S10.** **The quality evaluation of assembled genome by BUSCO software.**

| Library | Eukaryota | Metazoa |
| --- | --- | --- |
| Complete BUSCOs (C) | 237 | 868 |
| Complete and single-copy BUSCOs (S) | 235 | 858 |
| Complete and duplicated BUSCOs (D) | 2 | 10 |
| Fragmented BUSCOs (F) | 2 | 8 |
| Missing BUSCOs (M) | 16 | 78 |
| Total BUSCO groups searched | 255 | 954 |
| Summarize (%) | 93.0% | 90.9% |

**Table S11. Comparison of the related genomes with our chromosome-level genome**.

| Species | Genome source | Assembly level | Genome size | Genome N50 | Scaffold/Chromosome number | BUSCO (eukaryota_odb10) |
| --- | --- | --- | --- | --- | --- | --- |
| *L. elegans* | This study | Chromosome | 1,566,804,893 | 114,313,277 | 164 | 93.0% |
| *A. geniculata* | NCBI (GCA_000661875.1) | Scaffold | 7,178,402,394 | 20,294 | 4,986,575 | 22.0% |
| *S. mimosarum* | NCBI (GCA_000611955.2) | Scaffold | 2,738,704,917 | 480,636 | 68,653 | 91.0% |
| *T. clavipes* | NCBI (GCA_002102615.1) | Scaffold | 2,439,301,466 | 62,959 | 180,236 | 68.6% |
| *A. bruennichi* | NCBI (GCA_015342795.1) | Chromosome | 1,670,285,661 | 124,235,998 | 2,231 | 91.4% |
| *P. tepidariorum* | NCBI (GCF_000365465.2) | Scaffold | 1,445,396,121 | 4,055,356 | 16,533 | 92.9% |
| *C. sculpturatus* | NCBI (GCF_000671375.1) | Scaffold | 925,474,958 | 537,465 | 8,338 | 92.5% |
| *S. dumicola* | NCBI (GCF_010614865.1) | Scaffold | 2,551,871,595 | 254,130 | 16,531 | 80.0% |
| *I. scapularus* | NCBI (GCF_016920785.1) | Scaffold | 2,296,047,215 | 1,735,392 | 2,977 | 96.5% |
| *T. antipodiana* | GigaDB | Scaffold | 2,294,533,288 | 172,892,172 | 377 | 96.5% |

**Table S12. The statistics of the annotated repeat sequences in our assembled genome.** The type represents that the way or software used in this study.

| Type | Repeat Size | % of genome |
| --- | --- | --- |
| Trf | 61,782,967 | 3.943246 |
| Repeatmasker | 3,814,684 | 0.243469 |
| Proteinmasker | 60,339,137 | 3.851094 |
| De novo | 476,094,387 | 30.386322 |
| Total | 506,088,585 | 32.300677 |

**Table 13. The statistics of the annotated repeat sequences in our assembled genome by de novo prediction.**

|  | Repbase TEs | | TE protiens | | De novo | | Combined TEs | |
| --- | --- | --- | --- | --- | --- | --- | --- | --- |
| Type | Length (bp) | % in genome | Length (bp) | % in genome | Length (bp) | % in genome | Length (bp) | % in genome |
| DNA | 621,915 | 0.039693% | 4,975,289 | 0.317544% | 149,010,813 | 9.510489% | 151,778,650 | 9.687144% |
| LINE | 457,988 | 0.029231% | 30,759,634 | 1.963208% | 67,297,459 | 4.295204% | 70,533,283 | 4.501727% |
| SINE | 418 | 0.000027% | - | - | 17,144,782 | 1.094251% | 17,145,200 | 1.094278% |
| LTR | 2,679,897 | 0.171042% | 24,597,184 | 1.569895% | 31,374,094 | 2.002425% | 38,889,578 | 2.482094% |
| Other | 54,260 | 0.003463% | 10,927 | 0.000697% | 55,734,325 | 3.557196% | 55,746,684 | 3.557985% |
| UnKnown | 42 | 0.000003% | - | - | 159,932,926 | 10.207584% | 159,932,968 | 10.207587% |
| Total | 60,339,137 | 3.851094% | 3,814,401 | 0.243451% | 476,094,387 | 30.386322% | 486,115,304 | 31.025899% |

**Table S14. The functional annotation of the predicted protein-coding genes.**

| Database | Number | Percentage (%) |
| --- | --- | --- |
| InterPro | 10,666 | 52.89% |
| GO | 8,157 | 40.45% |
| KEGG | 10,771 | 53.41% |
| Swissprot | 11,933 | 59.17% |
| TrEMBL | 15,696 | 77.83% |
| Cog | 7,350 | 36.45% |
| Nr (NCBI) | 16,166 | 80.16% |
| Annotated | 16,341 | 81.03% |
| Unanotated | 3,826 | 18.97% |
| Total | 20,167 | - |

**Table S15. Relative evolution rate among these species by LINTRE software.**

| Outgroup | Ingroup1 | Ingroup2 | bA | bB | delta | Z score | CP (%) | Faster |
| --- | --- | --- | --- | --- | --- | --- | --- | --- |
| *I. scapularus* | *L. elegans* | *A. geniculata* | 0.498123 | 0.327810 | 0.170312 | 24.045846 | 99.96% | *L. elegans* |
|  |  | *A. bruennichi* | 0.211800 | 0.158487 | 0.053313 | 10.619045 | 99.96% | *L. elegans* |
|  |  | *P. tepidariorum* | 0.170848 | 0.141527 | 0.029320 | 6.391144 | 99.96% | *L. elegans* |
|  |  | *T. antipodiana* | 0.214634 | 0.154331 | 0.060303 | 12.502131 | 99.96% | *L. elegans* |
|  |  | *T. clavipes* | 0.188508 | 0.173370 | 0.015138 | 2.922411 | 99.64% | *L. elegans* |
|  |  | *C. sculpturatus* | 0.415612 | 0.311170 | 0.104442 | 16.467596 | 99.96% | *L. elegans* |
|  |  | *S. dumicola* | 0.219606 | 0.148210 | 0.071396 | 14.899019 | 99.96% | *L. elegans* |
|  |  | *S. mimosarum* | 0.211946 | 0.135934 | 0.076013 | 16.096112 | 99.96% | *L. elegans* |

**Table S16. Relative evolution rate among these species by MEGA software.**

| Outgroup | Ingroup1 | Ingroup2 | Identical | Ingroup1  specific | Ingroup2  specific | Chi-score | P-value | Faster |
| --- | --- | --- | --- | --- | --- | --- | --- | --- |
| *I. scapularus* | *L. elegans* | *A. geniculata* | 20,831 | 4,211 | 3,076 | 176.78 | <0.00001 | *L. elegans* |
|  |  | *A. bruennichi* | 28,528 | 3,688 | 2,828 | 113.51 | <0.00001 | *L. elegans* |
|  |  | *P. tepidariorum* | 29,753 | 3,066 | 2,585 | 40.94 | <0.00001 | *L. elegans* |
|  |  | *T. antipodiana* | 20,564 | 3,901 | 2,868 | 157.64 | <0.00001 | *L. elegans* |
|  |  | *T. clavipes* | 25,460 | 2,942 | 2,722 | 8.55 | 0.00346 | *L. elegans* |
|  |  | *C. sculpturatus* | 27,483 | 6,899 | 5,083 | 275.23 | <0.00001 | *L. elegans* |
|  |  | *S. dumicola* | 29,615 | 3,874 | 2,662 | 224.75 | <0.00001 | *L. elegans* |
|  |  | *S. mimosarum* | 28,442 | 3,606 | 2,354 | 263.00 | <0.00001 | *L. elegans* |

**Supplementary Table 17. Statistics of positively selected genes of *L. elegans*.**

| Gene name | A_w | B_w | S_w | delta_lnl | *P*-value |
| --- | --- | --- | --- | --- | --- |
| *COAC* | 0.02456 | 0.01616 | 1.04868 | 11.4426539999999 | 0.000717772 |
| *CaMKI* | 0.01191 | 0.01018 | 1.01845 | 10.8422619999992 | 0.000992095 |
| *RAB14* | 0.00186 | 0.00113 | 1.64727 | 4.6634779999999 | 0.030810720 |
| *PXDN* | 0.10280 | 0.08460 | 4.52074 | 15.5317220000006 | 0.000081132 |
| *PXDN* | 0.03566 | 0.00205 | 999.00000 | 5.5580780000000 | 0.018395600 |
| *lhx9* | 0.02088 | 0.01330 | 4.07144 | 3.9083380000000 | 0.048047092 |
| *Fbxo33* | 0.08062 | 0.07060 | 999.00000 | 4.8545519999998 | 0.027573274 |
| *FA43A* | 0.03063 | 0.02136 | 841.48260 | 9.6785579999996 | 0.001864309 |

**
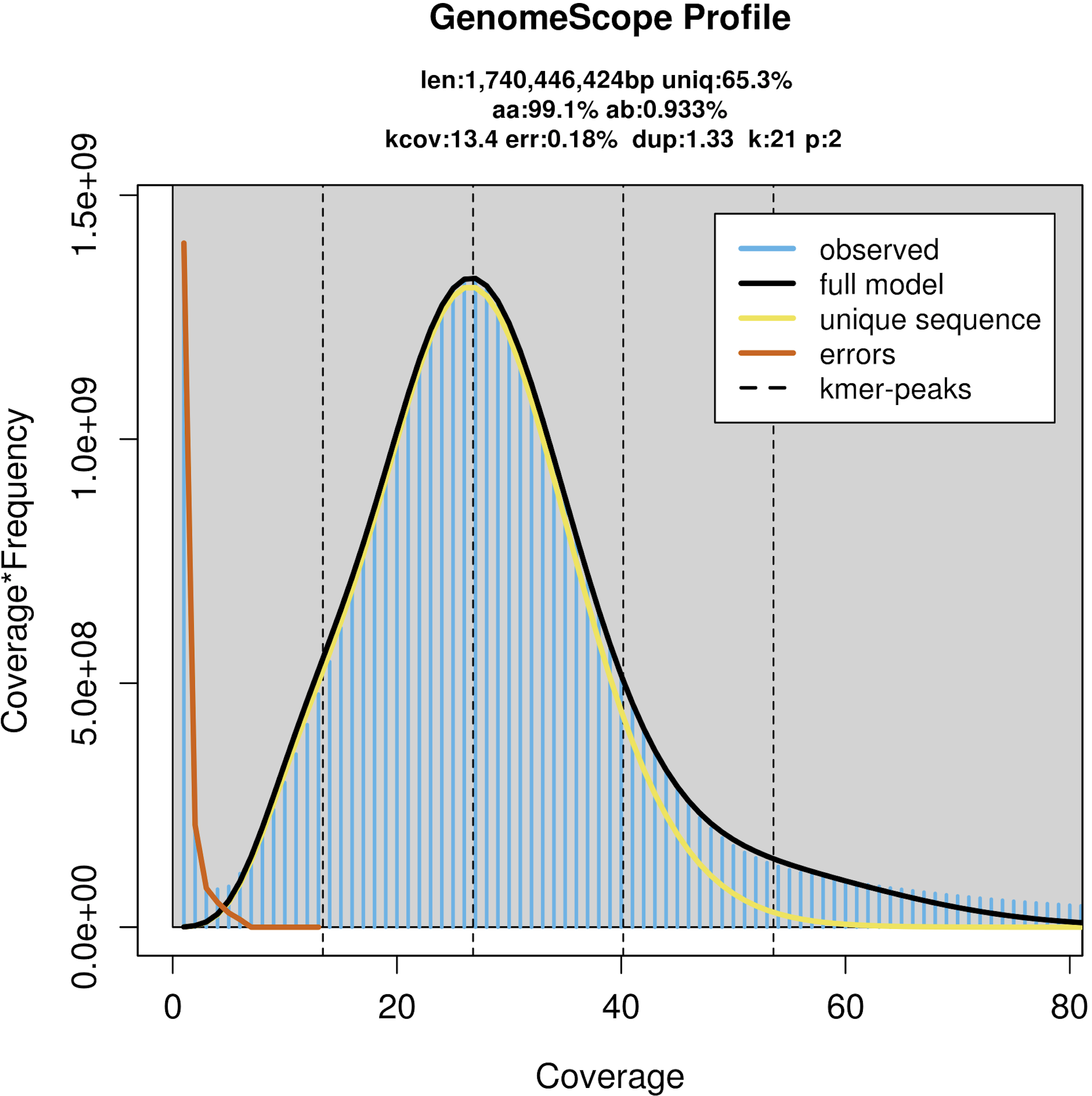
**

**Figure S1.** 21-mer analysis of *L. elegans* genome.

**
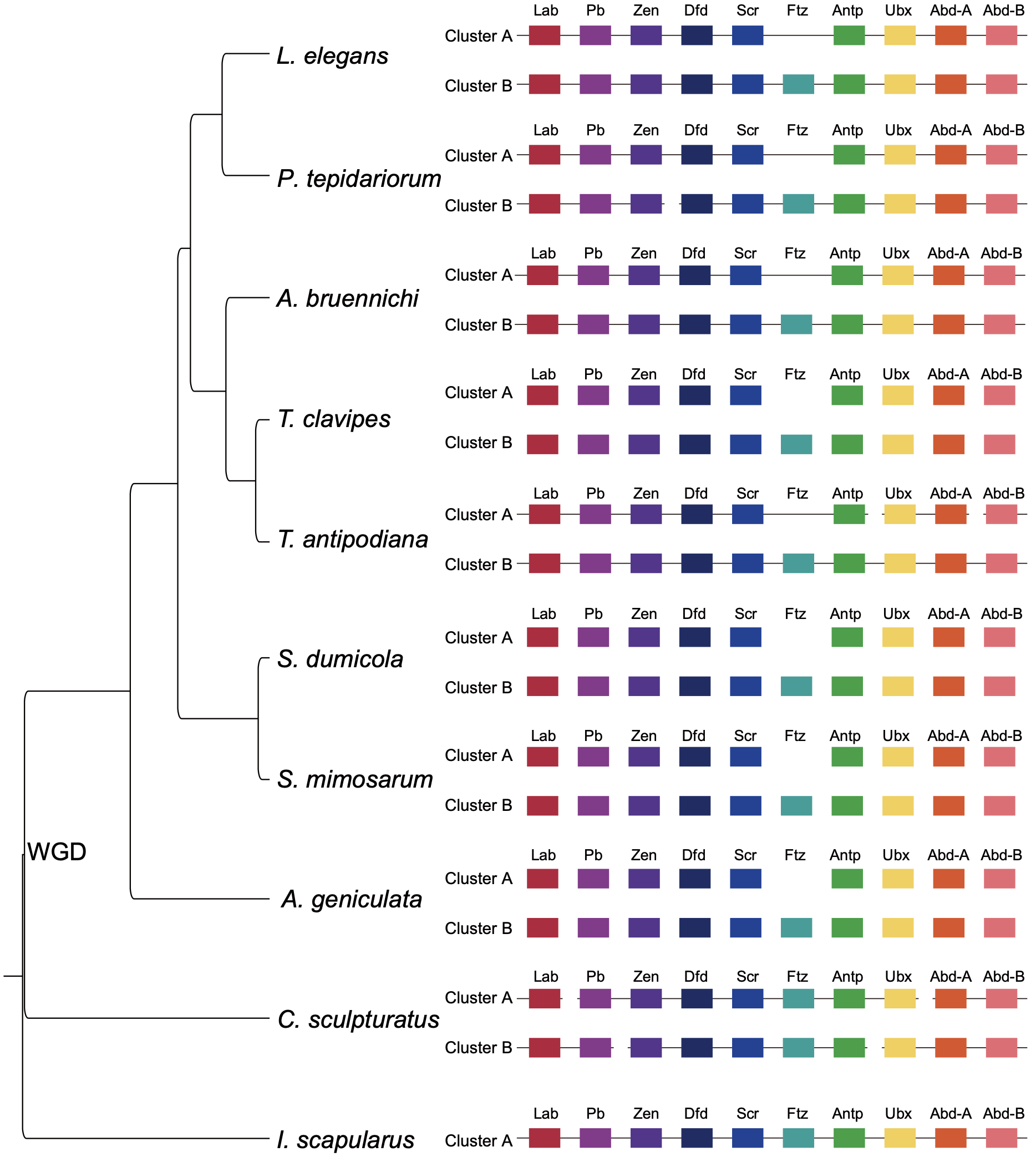
**

**Figure S2.** Annotation and comparison of the *Hox* clusters among these 10 species.

**
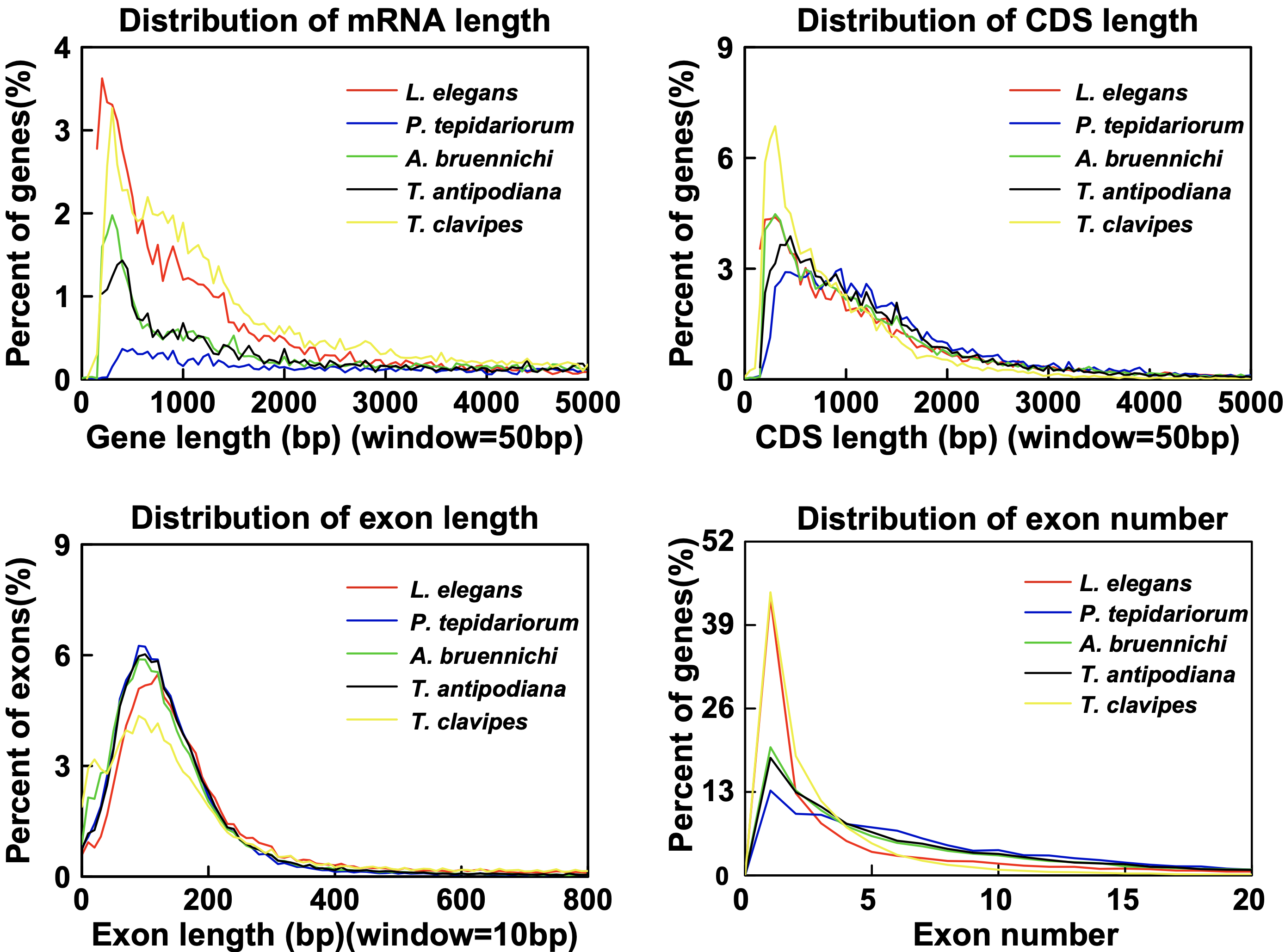
**

**Figure S3.** Distribution of gene parameters in various species.

**
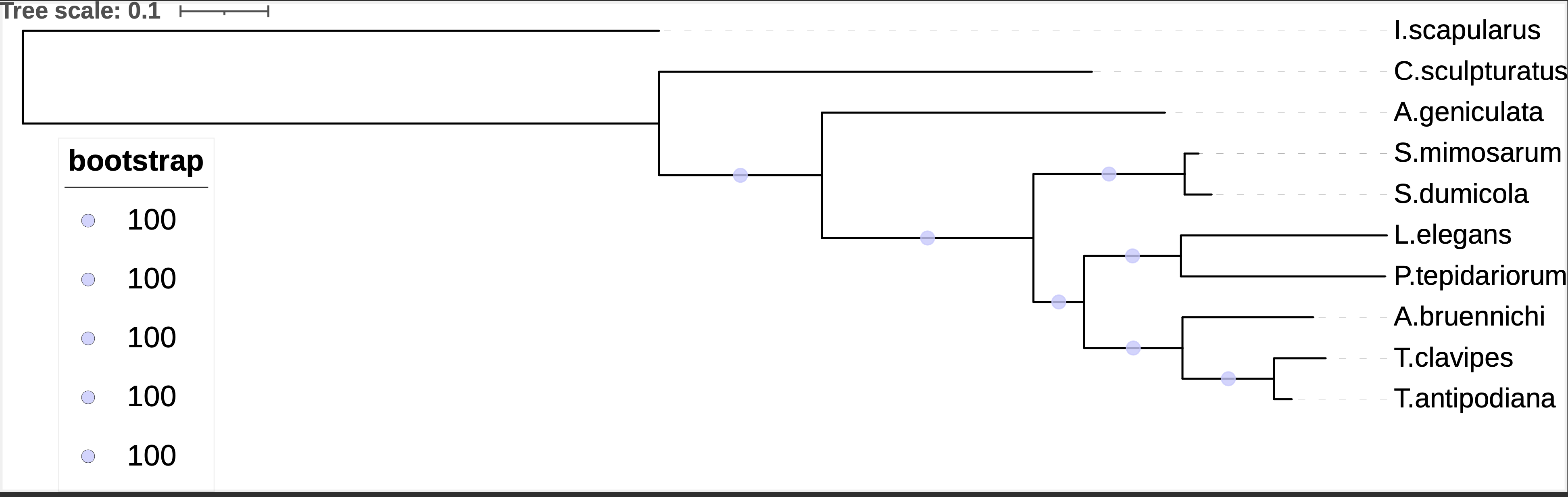
**

**Figure S4.** Phylogenetic relationship among the 10 species inferred by the amino acid sequences of the single-copy genes.

**
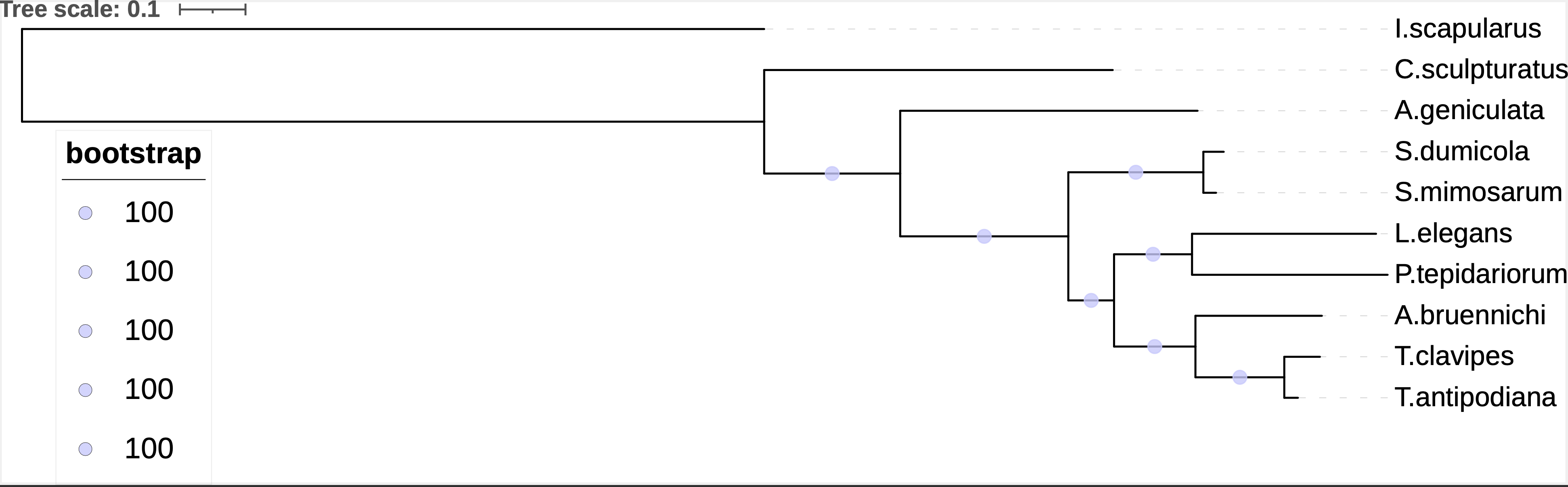
**

**Figure S5.** Phylogenetic relationship among the 10 species inferred by the nucleotide acid sequences of the single-copy genes.

**
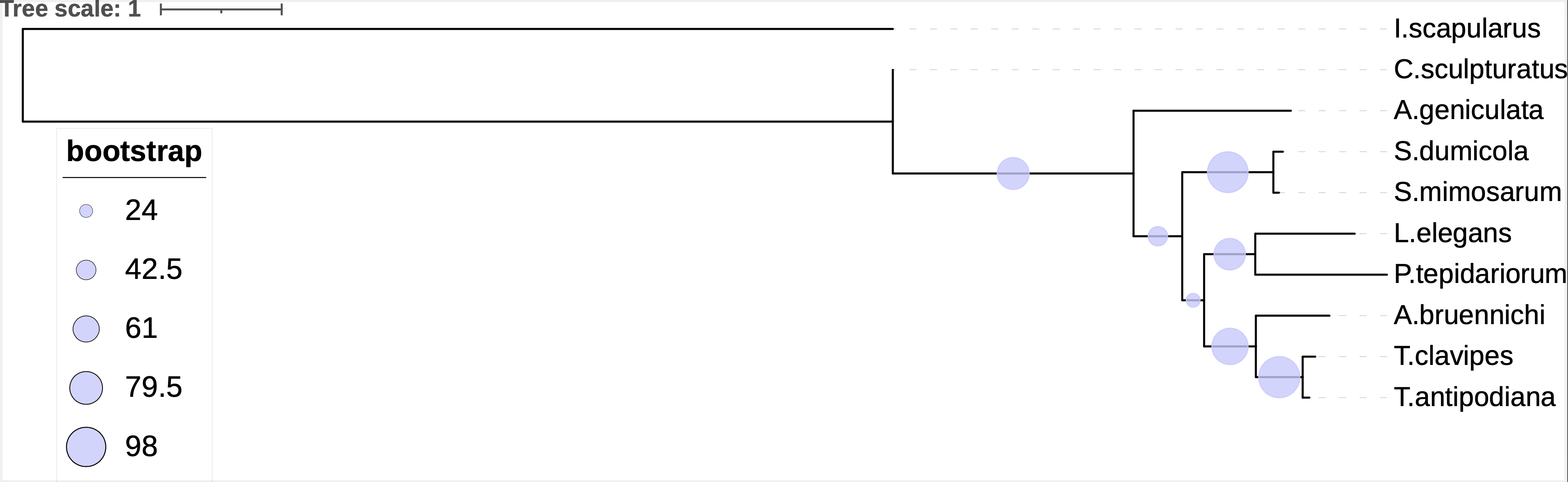
**

**Figure S6.** Phylogenetic relationship among the 10 species inferred by the 4dTV data of the single-copy genes.

**
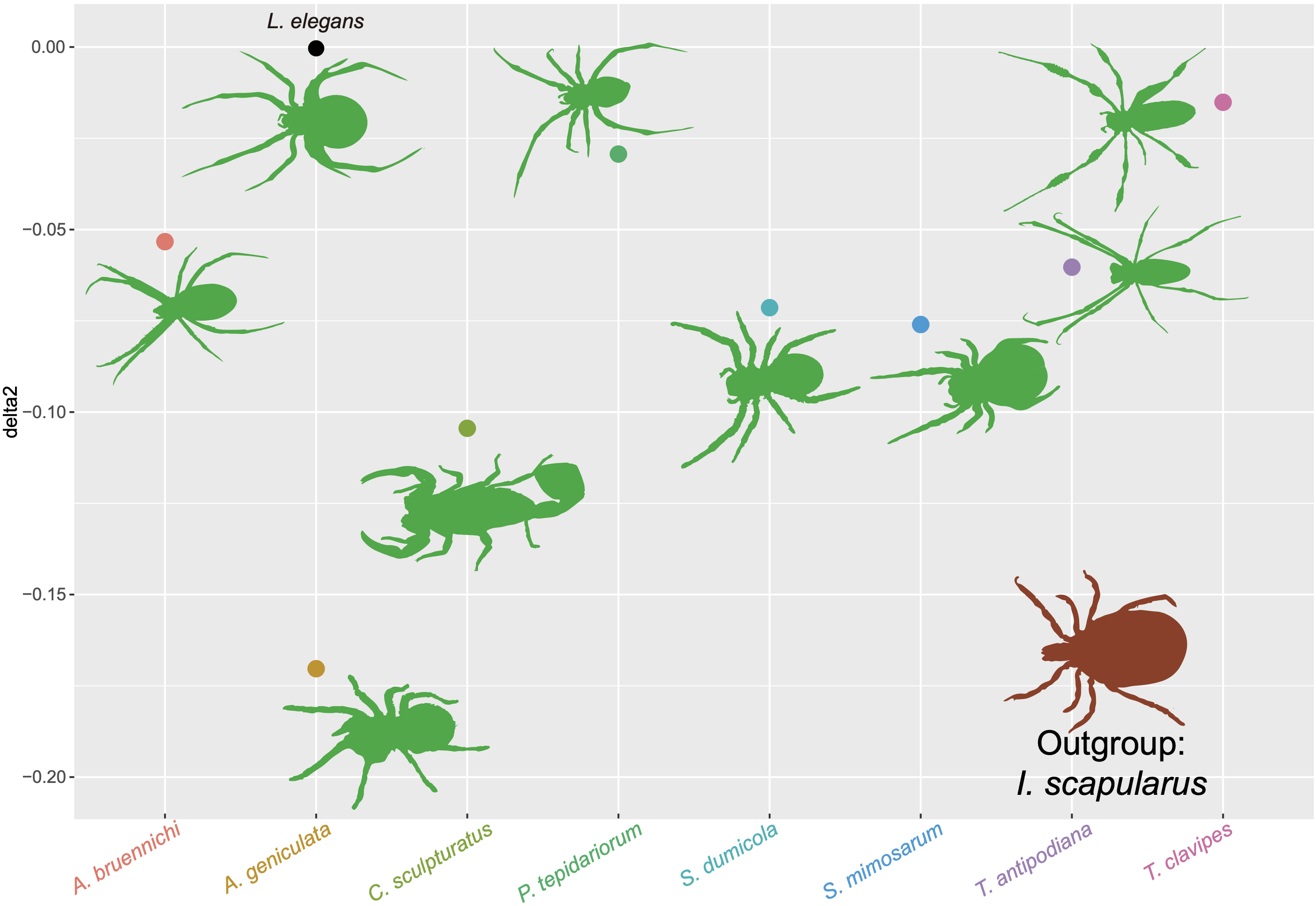
**

**Figure S7.** Relative evolutionary rate of species. The analysis was performed by the single-copy protein-coding genes among these species with *L. elegans* as the reference species and *I. scapularus* as the outgroup species. The y-axis showed the size of relative evolutionary rate of species.

**
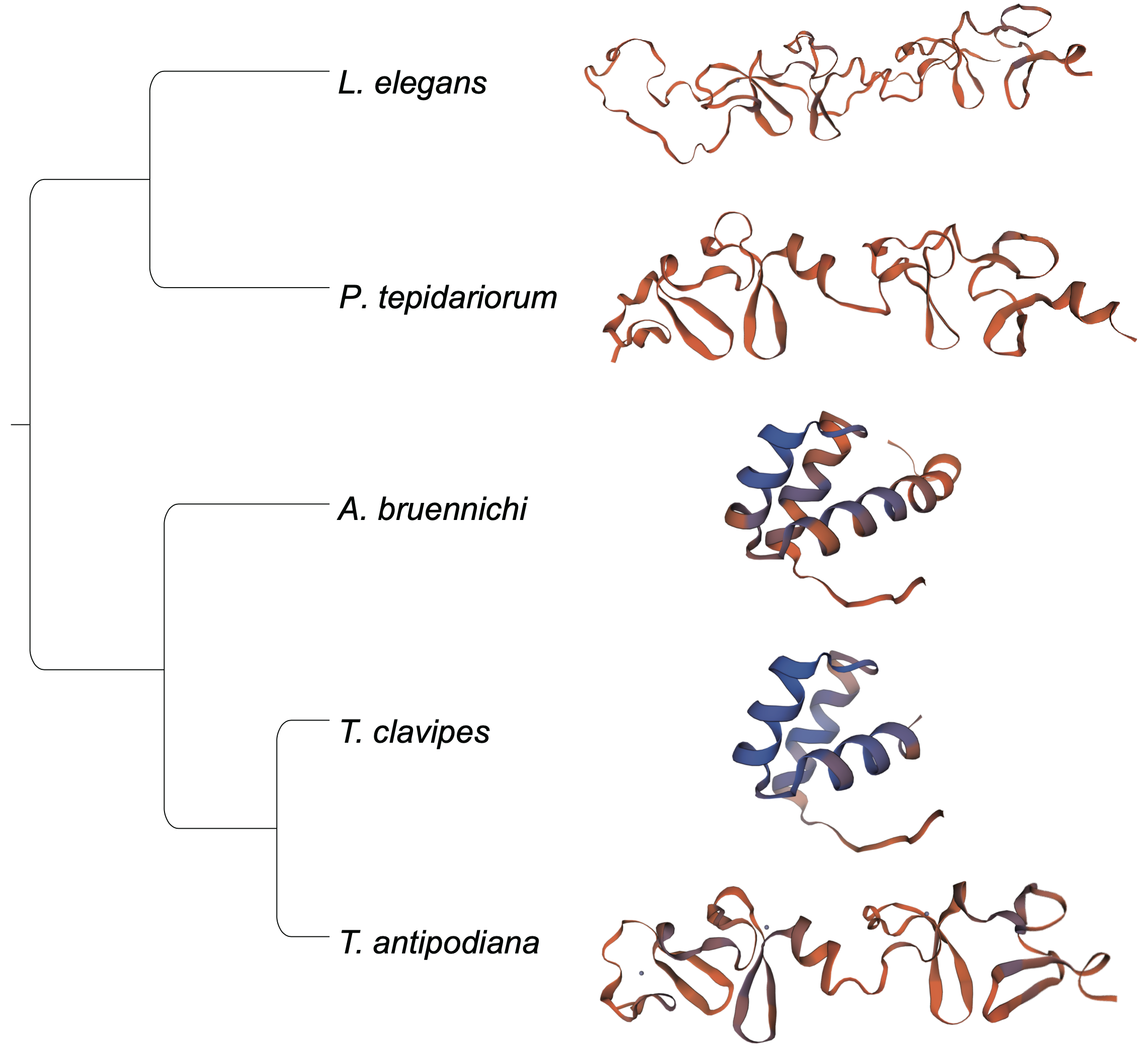
**

**Figure S8.** Gene structure of *lhx9* in these species.
