# Supplementary material for: Chromosome‐level genome assembly of the black widow spider Latrodectus elegans illuminates composition and evolution of venom and silk proteins
Source: Gigascience. 2022 May 25;11:giac049. doi: 10.1093/gigascience/giac049 (PMC9154082; doi:10.1093/gigascience/giac049)
Supplement: giac049_Response_to_Reviewer_Comments_Original_Submission [file giac049_response_to_reviewer_comments_original_submission.pdf]

Author's Response To Reviewer Comments

Close

Dear Editor,
Thank you and the three reviewers for your time and constructive comments on our manuscript " Chromosome-level genome assembly of the black widow spider Latrodectus elegans illuminates composition and evolution of venom and silk proteins" (GIGA-D-21-00338). These comments will greatly help improving our manuscript. Now we carefully revised our manuscript according to the reviewers' comment and polish the grammar in our revised manuscript. We wish that the updated manuscript could fit the reviewers' requirement. Meantime, we made the point-by-point responses to each of the comment from the editor and the reviewers, as follows: (We also attached a response letter file in our submission materal)

Editor:
Please also take a moment to check our website at https://www.editorialmanager.com/giga/ for any additional comments that were saved as attachments.
R: we checked the website and found no additional comments. Thank you for your kindly reminding.
In addition, please register any new software application in the bio.tools and SciCrunch.org databases to receive RRID (Research Resource Identification Initiative ID) and biotoolsID identifiers, and include these in your manuscript. This will facilitate tracking, reproducibility and re-use of your tool.
R: Thank you for your reminding. We checked all the softwares used in our manuscript and confirmed that there was no any new software used. Meantime, the electronic notebook-style methods document of these softwares were upload to the GigaDB dataset. We affirm that our tools are easy to track and reproducible.
Please ensure you describe additional experiments that were carried out and include a detailed rebuttal of any criticisms or requested revisions that you disagreed with. Please also ensure that your revised manuscript conforms to the journal style, which can be found in the Instructions for Authors on the journal homepage. If the data and code has been modified in the revision process please be sure to update the public versions of this too.
R: We have ensured all the above items.
Besides, we suggest you find a copy editing company or friendly native English speaker to polish the grammar.
R: We have made our manuscript polished by a copy editing company (Letpub).

Reviewer reports:
Reviewer #1: The manuscript described the genome of an orb-weaver spider Latrodectus elegans with a combination of Nanopore, Illumina and Hi-C sequencing and the comparison of latrotoxin genes and spidroin genes among spiders. The authors reported the assembly, quality and analysis of the genome in details and provided enormous information, in my opinion, useful to the research community.
However, I suggest a major revision before the work is published. The main concern is on the toxin identification.
R: Thank you very much for your overall positive comments to our manuscript and important suggestion.
I have one major concern related to the analysis of venom toxin genes. In Fig. 3 and related text, no ICK family gene was identified in five species including spider, scorpion and tick where ICK toxins have been reported and even functionally studied. In the other five spider species, fewer ICK genes were found (1 to 7). ICK family was considered an abundant toxin family in Arachnida and their homology is mainly dependent on the disulfide-bond instead of the primary sequence. The toxin genes were identified via blastp, which heavily relies on the sequence similarity. Similar concerns may apply to the other toxin families. I would suggest the authors redo the toxin gene analysis.
R: Thanks for your important comments and we are sorry for our carelessness in spider toxin identification. We investigated more on these toxins and learned that, as the reviewer stated, ICK toxins are a kind of small peptide and their homology is mainly dependent on the disulfide-bond instead of the primary sequence, unlike those proteins from CRISP family or Latrotoxin family. We then re-identified the ICK toxins through the following approaches. Firstly, we retrieved the peptides less than 200 amino

acid and have at least six cysteines constituting the NCNCNCCNCNC (N refers to any other amino acid) motif. Secondly, we used the online tool ClanTox (http://www.clantox.cs.huji.ac.il/index.php) to evaluate the toxin potential of the above candidate ICK toxins. Thirdly, we used SignalP (v5.0) to predict signal peptide of them and those without signal peptide was removed out. After further manual check, we finally identified the ICK toxins. As to Latrodectin family genes, there are rather few genes in ArachnoServer 3.0 database. We then retrieved the known sequences of Latrodectin from NCBI, then we applied the Blastp with e value cutoff of 10-5 to search the candidate homolog proteins. Then the homolog was further verified by hmmer (v3.3). In brief, all above-found sequences were merged together and applied Clustalw2 to perform the multiple sequence alignment which were then transformed into stockholm format using online toolset (https://novopro.cn/tools/format-conversion.html). The aligned sequences were used to construct the HMM profile using hmmbuild, a binary software of hmmer (v3.3). Finally, hmmsearch was applied for the peptide of each species, to find the potential domains of all species using key parameters "—domtblout and e-value of 10-5". Based on the updated methods, we did identify more ICK toxins. We updated the related methods and the results in the revised manuscript.

Minor errors:
1. Line 36, "remarkably more MiSp". It is grossly overwritten.
R: Thanks for reminding. We removed "remarkably" to downplay the statement.
2. Line 345-346, any reference?
R: We added a reference "Bhere KV, Haney RA, Ayoub NA and Garb J E. Gene structure, regulatory control, and evolution of black widow venom latrotoxins. FEBS Lett 2014; 588(21): 3891-3897." therein. Thanks for reminding.
3. Line 368, "We used the same method". What is the method? Same to what? There is not description in the above sentences.
R: Sorry for this rather poor writing. We removed these statements since we have described the method of spidroin genes in the Methods section. We modified the statements to "We identified six unique annotated genes for MaSp, eight for MiSp, two for Flag, five for AgSp, two for PySp, two for AcSp and one for TuSp in L. elegans".
4. Many typo are present in the main text. The manuscript could be improved by carefully revising the text and reformatting the reference list.
R: We are sorry for our carelessness. We carefully revised the main test and had let a copy editing company (Letpub) polish the grammar.

Reviewer #2: In this manuscript, the authors present a high-quality genome assembly of the species Latrodectus elegans, based on nanopore reads and HI-C scaffolding. In addition, they present the most relevant characteristics of the genome and its functional annotation, as well as a phylogenetic analysis that includes other arachnids, estimates of the evolutionary rate in different lineages, and focused on the chromosomal organization and evolution of some gene families of interest, such as toxins and spidroins.

I think the work is very interesting. This new genome provides new data that will be very useful to advance our understanding of the diversification of spiders and the evolution of some of the most relevant aspects of their biology. Nevertheless, I have some important concerns about different aspects of the ms, which in my opinion must be properly addressed before considering the manuscript suitable for publication in Gigascience.
R: Thank you very much for your interest of our manuscript and the instructive comments.

First of all, I think it is necessary to improve the language. I am not a native English speaker, but I think there are many sentences that are not well punctuated and some expressions are not well constructed. I highly recommend a thorough review of the entire text.
R: We are very sorry for our poor writing. We made a carefully revision and modification in writing. In addition, we had let a copy editing company polish our manuscript, in order to further improve it.
Apart from the language itself, there are poorly constructed sentences from the point of view of biological concepts. Phrases such as "Systematic identification of complete toxin gene sequence", in line 68, or "..will aid the identification and understanding of the complete spider toxin and spider silk genes..line 76", or "..to detect the genome integrity..line 117", or "transposon elements..line 121"..or "connected into one super gene..line 169", or "all merged values were plotted and grouped by species...line 210", or "the chromosome distribution of spidroin were plotted...line 229", or "..genes that had homolog genes in the public database..line 264", or "the original group of arachnida..line 327"..etc..
R: Line 68: we modified it to: "Systematic identification and analysis of all the spider toxin genes with full length sequences".

Line 76: we modified it to: "…will provide important resources for deciphering the spider toxin and spidroin genes".
Line 117: we modified it to: "..to evaluate the genome integrity."
Line 121: we modified it to: "transposable elements".
Line 169: we modified the sentence to: "All the well-aligned single-copy orthologous genes in each species were concatenated to one super gene for each species."
Line 210: we modified it to: "all the Ka/Ks values were plotted and grouped by species." We also made other modifications in the context.
Line 229: we modified it to: "The chromosome distribution of the spidroins were then plotted ...".
Line 264: we modified it to: "... genes having homologous hits in the public databases."
Line 327: we modified it to "... It is the only species from the relatively ancient Mygalomorphae. The result suggests that relatively ancient spider group may have relatively less selection pressure in their habitats. However, due to its low genome quality, this result is pending further verification....".
In addition, we carefully checked the whole manuscript and made intensive revisions of it.

Regarding specific contents, there are some things that need to be revised, rewritten or expanded. Here are some examples:
In line 137 a sentence begins with "the protein sequences of ...." and then, after the comma, continues with "... the longest transcript were chosen for further analyzes. Something is wrong here ...
R: We modified the statement to: "We chose the longest isoform of each gene to obtain the non-redundant protein sequences of each gene set." We also made modifications in the context.
In line 143, the phrase "All the remaining genes were aligned using blastn" is not understood, what do the authors mean?
R: We modified the related statements to: "We chose the longest isoform of each gene to obtain the non-redundant protein sequences of each gene set. The protein sequences were used as query to search for orthologous regions in the L. elegans genome by tblastn with an e-value of 1e-5. The results were subjected to GeneWise software (v2.4.1) (Birney and Durbin 2000) to predict gene structure."
Various lines. The names and identifiers of the species used in the different analyzes are unnecessarily repeated in several paragraphs.
R: Thanks for reminding. We carefully checked the manuscript and removed the repeated names and identifiers of the species.
line 150. Please, replace "homologous searching" with "sequence similarity-based searches" or similar...
R: Thanks. We replaced "homologous searching" with" homology-based searches".
Line 183. "Using ..... gene pairwise relationships". What do the authors mean by this phrase? What pairwise relationships do they use to determine the evolution of families in CAFE?
R: We are sorry for this vague description. However, in order to limiting genomic analysis and make the manuscript meet the requirement of "Date note" in Gigascience, we removed unnecessary genomic analysis, including analysis on gene family expansion and contraction. So this sentence was removed.
line 190. "To identify the potential positively selected genes (PSGs) and the genes with positively selected sites ..." I do not understand the difference between these two classes of genes.
R: Sorry that we didn't describe it clear. We used different models in Codeml tool of PAML package (v4.8) to detect potential positively selected genes (PSGs) and the genes with positively selected sites. PSGs were those identified by two ratio branch model. Those not identified as PSGs, were then subjected to branch site model to detect whether they have positive selected sites. We re-wrote the method of positive selection analysis: "Single-copy orthologs from the five relatively closely related species of orb-web weaving spiders, i.e., L. elegans, P. tepidariorum, A. bruennichi, T. clavipes and T. antipodiana. As well as their species tree were used to identify the potential positively selected genes (PSGs) and the genes with positively selected sites in L. elegans, using branch model and branch site model in the Codeml tool of the PAML package (v4.8) (Yang 1997) respectively. Briefly, the rate ratio (ω) of nonsynonymous to synonymous nucleotide substitutions was estimated. One ratio model was used to detect average ω across the species tree (ω0). For each gene, two ratio branch model was used to detect the ω of the appointed branch (L. elegans) to test the (ω1) and ω of all other branches (ω_background). A likelihood ratio test was performed to compare the fit of the two ratio models with the one ratio model to determine whether the gene is positively selected in the appointed branch ($\omega1>\omega0$; $\omega1>\omega\_background$; $\omega1>1$; $P\text{-value}<0.05$). If the gene is not positively selected, then the branch site model was used to detect amino acid sites likely to be is positively selected in the appointed branch using Bayes Empirical Bayes (BEB) analysis (Yang et al., 2005). "
Line 229. It should say "distribution of spidroins" ...
R: Thanks and we revised it.
In material and methods, for example, much information is lacking on how or with what software some things have been done. For example, the exact location where the sample was obtained is missing, now only the province is specified (line 87). Authors do not specify where the sequencing has been done

(lines 91 and 92). The software used to filter the nanopore reads is missing (lines 96 and 97). Are the Perl scripts used written specifically for the project (in house-scripts) or are part of other software (line 96)?. It is not very clear for me what the Kimura value means (line 130), is it the corrected evolutionary distance using a kimura model? and, if true, which model, 2P or 3P?). Is the r8s program really implemented in the PAML package? (line 132).
R: Line 87: the spider is collected in Binzhou county, Dali City, Yunnan province.
Lines 91 and 92: we sent the live sample to Beijing Biomarker Technologies. Co. LTD for sequencing.
Lines 96 and 97: the Perl script used for filter the Nanopore reads is written specifically for this project (in house-script). We added it in our electronic notebook-style methods document and upload to the GigaDB dataset.
Line130: Yes, Kimura value is the corrected evolutionary distance using a kimura model. We used the parseRM (https://github.com/ 4ureliek/Parsing-RepeatMasker-Outputs) to conduct the kimura-distance based analysis on TE insertion time. We checked the pipeline and confirmed that 2P model is used therein. The principle is from the reference: Chalopin, D., Naville, M., Plard, F., Galiana, D., and Volff, J.-N. (2015). Comparative analysis of transposable elements highlights mobilome diversity and evolution in vertebrates. Genome Biol. Evol. 7, 567–580.
Line 132: We are sorry for this mistake. Yes, r8s is an individual program. Given that we re-wrote the description of TE analysis (The insertion time of each TE sequence was estimated using a Kimura distance based analysis (Chalopin, Naville et al. 2015) using parseRM (https://github.com/ 4ureliek/Parsing-RepeatMasker-Outputs)), this sentence was removed.
We added updated and revised information to the revised manuscript.

Other concerns:
The paragraphs titled Relative evolution calculation and Gene family expansion and contraction in the methods section are poorly written, it is hard to understand what they are doing.
R: Sorry for the poor descriptions. However, in order to limiting genomic analysis and make the manuscript meet the requirement of “Date note” in Gigascience, we removed unnecessary genomic analysis, including analysis on gene family expansion and contraction. So this sentence was removed.
Lines 183-184. "to identify gene families significantly expanded or contracted"
R: Thanks. However, in order to limiting genomic analysis and make the manuscript meet the requirement of “Date note” in Gigascience, we removed unnecessary genomic analysis, including analysis on gene family expansion and contraction. So this sentence was removed.
In the paragraph titled Positive Selection Analysis in the methods section, please specify what specific evolutionary models have been used.
R: Sorry for the vagueness. We re-wrote these contents and clearly described the models used.
Why are different compilations of RAxML (AVX or PHREADS) used in different parts of the job?
R: In our manuscript, two different compilations were used because they were run on different clusters, which were “raxmlHPC-PTHREADS-AVX” and “raxmlHPC-PTHREADS”. These are in fact of no difference in performance for both are PTHREADS version, and the AVX complication means the processors support AVX vector instructions which is faster in operation. To satisfy the needs of different part of the work, we changed our processors and the corresponding raxml complications.
Were the 14 scaffolds obtained in the assembly expected?
R: Yes, they were. The female black widow spiders are reported to have 28 chromosomes.
Is there information on karyotypes of this species?
R: Although there is no karyotype of this species, there is evidence that female black widow spiders have 28 chromosomes. Thanks for your reminding and we added the reference (Zhao YN, Ayoub A, Hayashi CY. Chromosome mapping of dragline silk genes in the genomes of widow spiders (Araneae, Theridiidae). PLoS One 2010; 5(9): e12804.), and the related statement to the revised manuscript.
What software has been used to make the graphs of the syntenic relationships between assemblies? That information is not in methods.
R: Sorry for this problem, softwares Last (v1066) and Circos (v0.69-6) were used. To perform the whole genome synteny analysis, we implemented the last software (v1066) to perform the whole genome alignment using L. elegans assembly as database, in which the “lastal” command was first used to obtain MAF format alignment files and the “maf-swap” command was then used to sort the alignment and select the best one-to-one blocks. After that, Circos (v0.69-6) was used to plot the syntenic relationship graph. Thank you for your reminding, we have added it in the method part of the manuscript.
Line 277. CDS has not been defined previously. Where does the acronym 4dTV come from?
R: Sorry for the miss, we have added the definition of CDS in the manuscript. A nucleotide site is classified as nondegenerate, twofold degenerate, or fourfold degenerate (4dTV), depending on how often nucleotide substitutions will result in amino acid replacement, we have cited the literature in the manuscript.

Line 279. "High boostrap values" ... not results, results are not high.
R: Sorry for this poor description. We have corrected it in the manuscript.
To what extent is the phylogeny obtained in this work different from those already known? What is the contribution of this part of the work to the knowledge of the diversification of these species?
R: The phylogeny obtained in this work is consistent to previously reported pattern. We added information in the species in our study to spider tree of life, especially estimating the divergent time between it and the relative Theridiidae spider, P. tepidariorum.
In the paragraph titled "Gene family expansion and contraction of L. elegans" of results and discussion (see comment on lines 183-184 for a more correct title), the p-value <0.05, is a value corrected by the multiple testing the many families, or does it mean that the p value of each family is <0.05? Consider this problem if not. In fact, much information is lacking on how the turnover rate analysis has been done in this study. Please, expand the method section where this aspect is discussed.
R: Thanks for your comments. In order to limiting genomic analysis and make the manuscript meet the requirement of “Date note” in Gigascience, we removed unnecessary genomic analysis, including analysis on gene family expansion and contraction, and highlighted analyses on toxin and spidroin genes.
Lines 304-307. Please explain the reason for this statement or provide references.
R: Thanks for your comments. In order to limiting genomic analysis and make the manuscript meet the requirement of “Date note” in Gigascience, we removed unnecessary genomic analysis, including analysis on gene family expansion and contraction. So this content was removed.
Line 311. A. geniculata is not an ancient spider! Perhaps the authors mean that it belongs to an ancient lineage.
R: We are very sorry for this mistake. Yes, we could not say A. geniculata is an ancient spider. This species belongs to Theraphosidae of the ancient Mygalomorphae, a relatively ancient lineage of Araneae. However, in order to limiting genomic analysis and make the manuscript meet the requirement of “Date note” in Gigascience, we removed unnecessary genomic analysis, including general analysis on homologs. So this sentence was removed.
Lines 310-311. "This means that most of the genes have orthologous genes ..." How does this phrase match the fact that the phylogeny has only been done with 156 single-copy genes? Are all the other families with other orthology relationships?
R: In the area of evolutionary genomics, homology genes are clustered into two parts, including ortholog and paralog. Especially, the former one, ortholog genes, are clustered mainly into single copy and multi copy parts. In the analysis of OrthoMCL, a classical gene family clustering software, five types are plotted, including single copy orthologs, multiple copy orthologs, other orthologs, unique paralogs, and unclustered genes. Considering the genome quality of A. geniculate, it is not figured into this description. That means most of the genes have orthologous genes, but only 156 genes are clustered into single-copy orthologs.
However, in order to limiting genomic analysis and make the manuscript meet the requirement of “Date note” in Gigascience, we removed unnecessary genomic analysis, including general analysis on homologs. So this sentence was removed.
Line 320-321. Could it be the result of differences in the quality of annotations and assemblies rather than unique characters of this species?
R: Obviously, we could not exclude the possibilities. These could be caused by a lot of reasons, like sequencing error, assembly error, and even the computational errors of the servers or the clusters. However, based on the big data analysis, and the rapid development of sequencing and software technology, especially, our fully comparative genomics and evolutionary genomics analysis, we have sufficient confidence in the quality of our assembly and annotation.
However, in order to limiting genomic analysis and make the manuscript meet the requirement of “Date note” in Gigascience, we removed unnecessary genomic analysis, including general analysis on homologs. So this sentence was removed.
Line 327. ".this original group of arachnida ..", what does original mean? please review.
R: Sorry for the mistake. We revised the statements to: “Interestingly, A. geniculata had the slowest evolution rate. It is the only species from the relatively ancient Mygalomorphae. The result suggests that relatively ancient spider group may have relatively less selection pressure in their habitats”.
Line 332. "...eight positively selected genes and 384 genes with positively selected sites ..". In both cases genes are positively selected. Please better explain the difference and specify which models have been used.
R: Sorry for the vagueness description. We re-wrote the method and clearly indicate how to distinguish positively selected genes (PSGs) and genes with positively selected sites. We added the information on model use in PAML therein.
line 343. "..we identified four major spider venom family proteins ..". This sentence is badly constructed. Perhaps the authors meant ... "We identified proteins / copies / members of (the) four major venom

gene / protein families" ...
R: Sorry for the poor writing. We revised to: "We identified members of the four major venom gene families".
line 348. Please, indicate the p-value of these analyzes.
R: Thanks. However, considering to another review's comments, we need to limited unnecessary genomic analysis. We hence removed analysis on gene family expansion and contraction. Accordingly, we removed this sentence.

line 360. "We analyzed the nucleotide substitutions (Ks) .... and found that L. elegans laxotroxin genes had higher (Ka / Ks)". I don't understand the first (Ks).
R: We are sorry for this misleading typo. It is the Ka/Ks rather than Ks. We revised the sentence to: "We analyzed the ratio of nonsynonymous to synonymous nucleotide substitution rate (Ka /Ks ratio) of each laxotroxin gene pair and found that L. elegans laxotroxin genes had generally higher Ka/Ks ratios compared to those in other species,…" .
line 371. "relatively more ..." is not a particularly scientific expression.
R: We are sorry for this unprofessional expression. We removed "relatively".
Conclusions: I believe that many of the conclusions of this ms are not justified or are not supported by the results. They are more the result of speculation and interpretation. They should focus more on the description of the genome and its characteristics. The last sentences should move to the introduction.
R: Sorry for our poor writing. We updated the conclusion:
"Using the approach combining data of Illumina short reads, Nanopore long reads and Hi-C, we assembled and annotated the first chromosome-level 1.57 Gb large genome of a black widow spider, L. elegans. In this study, we confirmed phylogenetic position of this species in the spider tree of life. In addition, by analysis on hox gene family, we again verified it high quality. Specifically, we focused on toxin and spidroin genes, which contribute to the distinctive features of black widow and cob web-weaving spiders and provided substantial information in terms of their composition and numbers and preliminarily demonstrate the evolution pattern of one important toxin gene family, latrotoxins. The important venom toxins contribute greatly to black widow spider's toxicity and they showed fast evolution. Generally, the genome resource data will help for deep exploration of spider genome evolution, specially on diversification of veom and web-forming. The sequence data are also first-hand templates for further application of the spider biomass."

Reviewer #3: Chromosome-level genome assembly of the black widow spider Latrodectus elegans illuminates composition and evolution of venom and silk proteins

This manuscript described the sequencing and assembly of a black widow spider, and a number of analyses comparing the obtained reference genome sequence to other spider genomes.
I realize that the manuscript is submitted as 'Data note', but a number of genome analyses are performed, and it is not very clear to me what the overall questions are that the authors want to address.
R：Thanks for your reminding. The species used in our study is a kind of black widow spider, with well-known of its venom. It is also featured with its distinctive web, which is three-dimensional and called cob-web. We hence provided more information on the venom toxins and spidroins of this species, in order to highlight the importance of the genome resource. We noticed that a recent data note published note on Gigascience showed similar kind of information (such as: Sheffer MM, Hoppe A, Krehenwinkel H, Uhl G, Kuss AW, Jensen L, Jensen C, Gillespie RG, Hoff KJ and Prost S. Chromosome-level reference genome of the European wasp spider Argiope bruennichi: a resource for studies on range expansion and evolutionary adaptation. Gigascience 2021;10: 1-12.). According to your comment, we limited unnecessary genomic analysis. For instances, we removed analysis on gene family expansion and contraction, as well as general analysis on homologs. We also made modifications of our manuscript and made it meet the format requirement of "Date Note".
The conclusion in the abstract is not a conclusion; just says 'this data is valuable', but not how/why.
R: We modified the whole content of the abstract. Specifically, the revised conclusion therein was: "We present the first chromosome-level genome assembly of a black widow spider and provided substantial toxin and spidroins gene resources. These high-qualified genomic data add valuable resources from a distinctive spider group and contribute to deep exploration on spider genome evolution, especially in terms of the important issues on diversification of venom and web-weaving pattern. The sequence data are also first-hand templates for further application of the spider biomass."
Line 47: what does it mean 'due to their adaptability'? It sounds like they special with regard to adaptation. Is this so?
R: Sorry for the misleading statement. Mechanism underlying wide distribution of spiders is a big issue and could not simply be attributed to adaptability and diverse behavior. We cautiously removed this

statement.
Line 73: the authors say 'full length spidroin sequences have been found' but no references. Here are some options:
-https://www.nature.com/articles/ncomms4765
-https://www.nature.com/articles/ng.3852
-https://academic.oup.com/gigascience/article/10/1/giaa148/6067174?login=true
R: We had cited two references which were shown in the end of the sentence. Thanks for providing the references. Since "https://www.nature.com/articles/ncomms4765": the spidroin sequences in this paper is not full-length, we added the other two papers to our revised manuscript.
Line 309: The A. geniculata genome is of much lower quality than the other genomes. Therefore, the conclusions given in this section should be removed. This should also apply to line 326.
R: Thanks for your reminding. In order to limiting genomic analysis and make the manuscript meet the requirement of "Date note" in Gigascience, we removed unnecessary genomic analysis, including general analysis on homologs. So this content was removed.
I think that you should cite the papers publishing all the spider genome sequences (plus I. scapularis and C. sculpturatus) that you download and analyze.
Generally, I think that the manuscript uses too few citations. Another example is line 256 where the authors say that the results of their hox gene analysis are similar to others species, but without any references. Actually, I do not think there is a single reference in the result/discussion section, but may have missed it/them!!!
R: Thanks for your reminding. We made intensive modifications in the manuscript and added quite a few new references to support our results and discussion. Line 256, we added the references: Schwager EE, Sharma PP, Clarke T, et al. The house spider genome reveals an ancient whole-genome duplication during arachnid evolution. BMC Biol. 2017; 15 (1):62. Published 2017 Jul 31. doi: 10.1186/s12915-017-0399-x; Sheffer MM, Hoppe A, Krehenwinkel H, et al. Chromosome-level reference genome of the European wasp spider Argiope bruennichi: a resource for studies on range expansion and evolutionary adaptation. Gigascience. 2021; 10 (1): giaa148. doi:10.1093/gigascience/giaa148; A chromosome-level genome of the spider Trichonephila antipodiana reveals the genetic basis of its polyphagy and evidence of an ancient whole-genome duplication event.
The figures are very busy with a lot of tiny text, and I have a hard time seeing that they read when fitted into a paper.
R: Sorry. We have enlarged the text to make the figures more readable.

Close
