# Supplementary material for: Chromosome‐level genome assembly of the black widow spider Latrodectus elegans illuminates composition and evolution of venom and silk proteins
Source: Gigascience. 2022 May 25;11:giac049. doi: 10.1093/gigascience/giac049 (PMC9154082; doi:10.1093/gigascience/giac049)
Supplement: giac049_Reviewer_1_Report_Original_Submission — Zewen Liu, Ph.D. -- 11/24/2021 Reviewed [file giac049_reviewer_1_report_original_submission.pdf]

Reviewer Report

Title: Chromosomeâ€level genome assembly of the black widow spider Latrodectus elegans illuminates composition and evolution of venom and silk proteins

Version: Original Submission **Date:** 11/24/2021

Reviewer name: Zewen Liu, Ph.D.

Reviewer Comments to Author:

The manuscript described the genome of a orb-weaver spider Latrodectus elegans with a combination of Nanopore, Illumina and Hi-C sequencing and the comparison of latrotoxin genes and spidroin genes among spiders. The authors reported the assembly, quality and analysis of the genome in details and provided enormous information, in my opinion, useful to the research community.
However, I suggest a major revision before the work is published.
The main concern is on the toxin identification.
I have one major concern related to the analysis of venom toxin genes. In Fig. 3 and related text, no ICK family gene was identified in five species including spider, scorpion and tick where ICK toxins have been reported and even functionally studied. In the other five spider species, fewer ICK genes were found (1 to 7). ICK family was considered an abundant toxin family in Arachnida and their homology is mainly dependent on the disulfide-bond instead of the primary sequence. The toxin genes were identified via blastp, which heavily relies on the sequence similarity. Similar concerns may apply to the other toxin families. I would suggest the authors redo the toxin gene analysis.
Minor errors:
1. Line 36, "remarkably more MiSp". It is grossly overwritten.
2. Line 345-346, Any reference?
3. Line 368, "We used the same method". What is the method? Same to what? There is not description in the above sentences.
4. Many typo are present in the main text. The manuscript could be improved by carefully revising the text and reformatting the reference list.

Level of Interest

Please indicate how interesting you found the manuscript: Choose an item.

Quality of Written English

Please indicate the quality of language in the manuscript: Choose an item.

Declaration of Competing Interests

Please complete a declaration of competing interests, considering the following questions:

- Have you in the past five years received reimbursements, fees, funding, or salary from an organisation that may in any way gain or lose financially from the publication of this manuscript, either now or in the future?
- Do you hold any stocks or shares in an organisation that may in any way gain or lose financially from the publication of this manuscript, either now or in the future?
- Do you hold or are you currently applying for any patents relating to the content of the manuscript?
- Have you received reimbursements, fees, funding, or salary from an organization that holds or has applied for patents relating to the content of the manuscript?
- Do you have any other financial competing interests?
- Do you have any non-financial competing interests in relation to this paper?

If you can answer no to all of the above, write 'I declare that I have no competing interests' below. If your reply is yes to any, please give details below.

I declare that I have no competing interests.

I agree to the open peer review policy of the journal. I understand that my name will be included on my report to the authors and, if the manuscript is accepted for publication, my named report including any attachments I upload will be posted on the website along with the authors' responses. I agree for my report to be made available under an Open Access Creative Commons CC-BY license (http://creativecommons.org/licenses/by/4.0/). I understand that any comments which I do not wish to be included in my named report can be included as confidential comments to the editors, which will not be published.

Choose an item.

To further support our reviewers, we have joined with Publons, where you can gain additional credit to further highlight your hard work (see: https://publons.com/journal/530/gigascience). On publication of this paper, your review will be automatically added to Publons, you can then choose whether or not to claim your Publons credit. I understand this statement.

Yes Choose an item.
