# Supplementary material for: Chromosome‐level genome assembly of the black widow spider Latrodectus elegans illuminates composition and evolution of venom and silk proteins
Source: Gigascience. 2022 May 25;11:giac049. doi: 10.1093/gigascience/giac049 (PMC9154082; doi:10.1093/gigascience/giac049)
Supplement: giac049_Reviewer_2_Report_Original_Submission — Alejandro SÃ¡nchez-Gracia -- 12/1/2021 Reviewed [file giac049_reviewer_2_report_original_submission.pdf]

Reviewer Report

Title: Chromosomeâ€level genome assembly of the black widow spider Latrodectus elegans illuminates composition and evolution of venom and silk proteins

Version: Original Submission **Date:** 12/1/2021

Reviewer name: Alejandro SÃ¡nchez-Gracia

Reviewer Comments to Author:

In this manuscript, the authors present a high-quality genome assembly of the species Latrodectus elegans, based on nanopore reads and HI-C scaffolding. In addition, they present the most relevant characteristics of the genome and its functional annotation, as well as a phylogenetic analysis that includes other arachnids, estimates of the evolutionary rate in different lineages, and focused on the chromosomal organization and evolution of some gene families of interest, such as toxins and spidroins. I think the work is very interesting. This new genome provides new data that will be very useful to advance our understanding of the diversification of spiders and the evolution of some of the most relevant aspects of their biology. Nevertheless, I have some important concerns about different aspects of the ms, which in my opinion must be properly addressed before considering the manuscript suitable for publication in Gigascience.

First of all, I think it is necessary to improve the language. I am not a native English speaker, but I think there are many sentences that are not well punctuated and some expressions are not well constructed. I highly recommend a thorough review of the entire text. Apart from the language itself, there are poorly constructed sentences from the point of view of biological concepts. Phrases such as "Systematic identification of complete toxin gene sequence", in line 68, or "..will aid the identification and understanding of the complete spider toxin and spider silk genes..line 76", or "..to detect the genome integrity..line 117", or "transposon elements..line 121"..or "connected into one super gene..line 169", or "all merged values were plotted and grouped by species...line 210", or "the chromosome distribution of spidroin were plotted...line 229", or "..genes that had homolog genes in the public database..line 264", or "the original group of arachnida..line 327"..etc..

Regarding specific contents, there are some things that need to be revised, rewritten or expanded. Here are some examples:

In line 137 a sentence begins with "the protein sequences of ...." and then, after the comma, continues with "... the longest transcript were chosen for further analyzes. Something is wrong here ...

In line 143, the phrase "All the remaining genes were aligned using blastn" is not understood, what do the authors mean?

Various lines. The names and identifiers of the species used in the different analyzes are unnecessarily repeated in several paragraphs.

line 150. Please, replace "homologous searching" with "sequence similarity-based searches" or similar ...

Line 183. "Using ..... gene pairwise relationships". What do the authors mean by this phrase? What pairwise relationships do they use to determine the evolution of families in CAFE?

line 190. "To identify the potential positively selected genes (PSGs) and the genes with positively

selected sites ..." I do not understand the difference between these two classes of genes.
Line 229. It should say "distribution of spidroins" ...
In material and methods, for example, much information is lacking on how or with what software some things have been done. For example, the exact location where the sample was obtained is missing, now only the province is specified (line 87). Authors do not specify where the sequencing has been done (lines 91 and 92). The software used to filter the nanopore reads is missing (lines 96 and 97). Are the Perl scripts used written specifically for the project (in house-scripts) or are part of other software (line 96)?. It is not very clear for me what the Kimura value means (line 130), is it the corrected evolutionary distance using a kimura model? and, if true, which model, 2P or 3P?). Is the r8s program really implemented in the PAML package? (line 132).
Other concerns:
The paragraphs titled Relative evolution calculation and Gene family expansion and contraction in the methods section are poorly written, it is hard to understand what they are doing.
Lines 183-184. "to identify gene families significantly expanded or contracted"
In the paragraph titled Positive Selection Analysis in the methods section, please specify what specific evolutionary models have been used.
Why are different compilations of RAxML (AVX or PHREADS) used in different parts of the job?
Were the 14 scaffolds obtained in the assembly expected?
Is there information on karyotypes of this species?
What software has been used to make the graphs of the syntenic relationships between assemblies? That information is not in methods.
Line 277. CDS has not been defined previously. Where does the acronym 4dTV come from?
Line 279.. "High boostrap values" ... not results, results are not high.
To what extent is the phylogeny obtained in this work different from those already known? What is the contribution of this part of the work to the knowledge of the diversification of these species?
In the paragraph titled "Gene family expansion and contraction of L. elegans" of results and discussion (see comment on lines 183-184 for a more correct title), the p-value <0.05, is a value corrected by the multiple testing the many families, or does it mean that the p value of each family is <0.05? Consider this problem if not. In fact, much information is lacking on how the turnover rate analysis has been done in this study. Please, expand the method section where this aspect is discussed.
Lines 304-307. Please explain the reason for this statement or provide references.
Line 311. A. geniculata is not an ancient spider!...perhaps the authors mean that it belongs to an ancient lineage.
Lines 310-311. "This means that most of the genes have orthologous genes ..." How does this phrase match the fact that the phylogeny has only been done with 156 single-copy genes? Are all the other families with other orthology relationships?
Line 320-321. Could it be the result of differences in the quality of annotations and assemblies rather than unique characters of this species?
Line 327. "..this original group of arachnida ..", what does original mean? please review.
Line 332. "... eight positively selected genes and 384 genes with positively selected sites ..". In both cases genes are positively selected. Please better explain the difference and specify which models have been used.

line 343. "..we identified four major spider venom family proteins ..". This sentence is badly constructed. Perhaps the authors meant ... "We identified proteins / copies / members of (the) four major venom gene / protein families" ...
line 348. Please, indicate the p-value of these analyzes.
line 360. "We analyzed the nucleotide substitutions (Ks) .... and found that L. elegans laxotroxin genes had higher (Ka / Ks)". I don't understand the first (Ks).
line 371. "relatively more ..." is not a particularly scientific expression.
Conclusions: I believe that many of the conclusions of this ms are not justified or are not supported by the results. They are more the result of speculation and interpretation. They should focus more on the description of the genome and its characteristics. The last sentences should move to the introduction.

Level of Interest

Please indicate how interesting you found the manuscript: Choose an item.

Quality of Written English

Please indicate the quality of language in the manuscript: Choose an item.

Declaration of Competing Interests

Please complete a declaration of competing interests, considering the following questions:

- Have you in the past five years received reimbursements, fees, funding, or salary from an organisation that may in any way gain or lose financially from the publication of this manuscript, either now or in the future?
- Do you hold any stocks or shares in an organisation that may in any way gain or lose financially from the publication of this manuscript, either now or in the future?
- Do you hold or are you currently applying for any patents relating to the content of the manuscript?
- Have you received reimbursements, fees, funding, or salary from an organization that holds or has applied for patents relating to the content of the manuscript?
- Do you have any other financial competing interests?
- Do you have any non-financial competing interests in relation to this paper?

If you can answer no to all of the above, write 'I declare that I have no competing interests' below. If your reply is yes to any, please give details below.

I declare that I have no competing interests

I agree to the open peer review policy of the journal. I understand that my name will be included on my report to the authors and, if the manuscript is accepted for publication, my named report including any

attachments I upload will be posted on the website along with the authors' responses. I agree for my report to be made available under an Open Access Creative Commons CC-BY license (http://creativecommons.org/licenses/by/4.0/). I understand that any comments which I do not wish to be included in my named report can be included as confidential comments to the editors, which will not be published.

Choose an item.

To further support our reviewers, we have joined with Publons, where you can gain additional credit to further highlight your hard work (see: https://publons.com/journal/530/gigascience). On publication of this paper, your review will be automatically added to Publons, you can then choose whether or not to claim your Publons credit. I understand this statement.

Yes Choose an item.
