# Supplementary material for: Chromosome‐level genome assembly of the black widow spider Latrodectus elegans illuminates composition and evolution of venom and silk proteins
Source: Gigascience. 2022 May 25;11:giac049. doi: 10.1093/gigascience/giac049 (PMC9154082; doi:10.1093/gigascience/giac049)
Supplement: giac049_Reviewer_3_Report_Original_Submission — Jesper Bechsgaard -- 12/7/2021 Reviewed [file giac049_reviewer_3_report_original_submission.pdf]

Reviewer Report

Title: Chromosomeâ€level genome assembly of the black widow spider Latrodectus elegans illuminates composition and evolution of venom and silk proteins

Version: Original Submission **Date:** 12/7/2021

Reviewer name: Jesper Bechsgaard

Reviewer Comments to Author:

Chromosome-level genome assembly of the black widow spider Latrodectus elegans illuminates composition and evolution of venom and silk proteins
This manuscript described the sequencing and assembly of a black widow spider, and a number of analyses comparing the obtained reference genome sequence to other spider genomes.
I realize that the manuscript is submitted as 'Data note', but a number of genome analyses are performed, and it is not very clear to me what the overall questions are that the authors want to address.
The conclusion in the abstract is not a conclusion; just says 'this data is valuable', but not how/why.
Line 47: what does it mean 'due to their adaptability'? It sounds like they special with regard to adaptation. Is this so?
Line 73: the authors say 'full length spidroin sequences have been found' but no references. Here are some options:
-https://www.nature.com/articles/ncomms4765
-https://www.nature.com/articles/ng.3852
-https://academic.oup.com/gigascience/article/10/1/giaa148/6067174?login=true
Line 309: The A. geniculata genome is of much lower quality than the other genomes. Therefore, the conclusions given in this section should be removed. This should also apply to line 326.
I think that you should cite the papers publishing all the spider genome sequences (plus I. scapularis and C. sculpturatus) that you download and analyze.
Generally, I think that the manuscript uses too few citations. Another example is line 256 where the authors say that the results of their hox gene analysis are similar to others species, but without any references. Actually, I do not think there is a single reference in the result/discussion section, but may have missed it/them!!!
The figures are very busy with a lot of tiny text, and I have a hard time seeing that they read when fitted into a paper.

Level of Interest

Please indicate how interesting you found the manuscript: Choose an item.

Quality of Written English

Please indicate the quality of language in the manuscript: Choose an item.

Declaration of Competing Interests

Please complete a declaration of competing interests, considering the following questions:

- Have you in the past five years received reimbursements, fees, funding, or salary from an organisation that may in any way gain or lose financially from the publication of this manuscript, either now or in the future?
- Do you hold any stocks or shares in an organisation that may in any way gain or lose financially from the publication of this manuscript, either now or in the future?
- Do you hold or are you currently applying for any patents relating to the content of the manuscript?
- Have you received reimbursements, fees, funding, or salary from an organization that holds or has applied for patents relating to the content of the manuscript?
- Do you have any other financial competing interests?
- Do you have any non-financial competing interests in relation to this paper?

If you can answer no to all of the above, write 'I declare that I have no competing interests' below. If your reply is yes to any, please give details below.

'I declare that I have no competing interests

I agree to the open peer review policy of the journal. I understand that my name will be included on my report to the authors and, if the manuscript is accepted for publication, my named report including any attachments I upload will be posted on the website along with the authors' responses. I agree for my report to be made available under an Open Access Creative Commons CC-BY license (http://creativecommons.org/licenses/by/4.0/). I understand that any comments which I do not wish to be included in my named report can be included as confidential comments to the editors, which will not be published.

Choose an item.

To further support our reviewers, we have joined with Publons, where you can gain additional credit to further highlight your hard work (see: https://publons.com/journal/530/gigascience). On publication of this paper, your review will be automatically added to Publons, you can then choose whether or not to claim your Publons credit. I understand this statement.

Yes Choose an item.
